# EpiCarousel: memory- and time-efficient identification of metacells for atlas-level single-cell chromatin accessibility data

**DOI:** 10.1093/bioinformatics/btae191

**Published:** 2024-04-08

**Authors:** Sijie Li, Yuxi Li, Yu Sun, Yaru Li, Xiaoyang Chen, Songming Tang, Shengquan Chen

**Affiliations:** School of Mathematical Sciences and LPMC, Nankai University, Tianjin 300071, China; School of Mathematical Sciences and LPMC, Nankai University, Tianjin 300071, China; Institute of Health Service and Transfusion Medicine, Beijing 100850, China; Institute of Health Service and Transfusion Medicine, Beijing 100850, China; MOE Key Laboratory of Bioinformatics and Bioinformatics Division of BNRIST, Department of Automation, Tsinghua University, Beijing 100084, China; School of Mathematical Sciences and LPMC, Nankai University, Tianjin 300071, China; School of Mathematical Sciences and LPMC, Nankai University, Tianjin 300071, China

## Abstract

**Summary:**

Recent technical advancements in single-cell chromatin accessibility sequencing (scCAS) have brought new insights to the characterization of epigenetic heterogeneity. As single-cell genomics experiments scale up to hundreds of thousands of cells, the demand for computational resources for downstream analysis grows intractably large and exceeds the capabilities of most researchers. Here, we propose EpiCarousel, a tailored Python package based on lazy loading, parallel processing, and community detection for memory- and time-efficient identification of metacells, i.e. the emergence of homogenous cells, in large-scale scCAS data. Through comprehensive experiments on five datasets of various protocols, sample sizes, dimensions, number of cell types, and degrees of cell-type imbalance, EpiCarousel outperformed baseline methods in systematic evaluation of memory usage, computational time, and multiple downstream analyses including cell type identification. Moreover, EpiCarousel executes preprocessing and downstream cell clustering on the atlas-level dataset with 707 043 cells and 1 154 611 peaks within 2 h consuming <75 GB of RAM and provides superior performance for characterizing cell heterogeneity than state-of-the-art methods.

**Availability and implementation:**

The EpiCarousel software is well-documented and freely available at https://github.com/biox-nku/epicarousel. It can be seamlessly interoperated with extensive scCAS analysis toolkits.

## 1 Introduction

Recent innovations in single-cell chromatin accessibility sequencing (scCAS) protocols, such as sci-assay for transposase-accessible chromatin with high-throughput sequencing (sci-ATAC-seq), have enabled the profiling epigenomic heterogeneity in hundreds of thousands of single cells for the interrogation and elucidation of gene regulation. For the analysis of scCAS data, computational pipelines, such as the widely used and comprehensive EpiScanpy package (Danese *et al.*, 2021), have been proposed. Nevertheless, one major issue with such conventional pipelines is that they are memory- and time-consuming when executing the standard analysis workflow on large-scale scCAS datasets.

Several computational methods have been proposed to tackle the increasing scale of single-cell RNA-seq (scRNA-seq) data by aggregating homogenous cells as a “metacell” for downstream analysis. For example, MetaCell partitions scRNA-seq data into statistically equivalent disjoint cell groups called metacells to delve into large data (Baran *et al.*, 2019). Metacell-2 further leverages a divide-and-conquer strategy to infer metacells, thereby enabling the analysis of scRNA-seq data at a scale of millions of cells (Ben-Kiki *et al.*, 2022). SEACells, the state-of-the-art method, utilizes kernel archetypal analysis to infer metacells for scRNA-seq data and can be generalized to scCAS data (Persad *et al.*, 2023).

However, most existing metacell identification methods were specifically designed for scRNA-seq data and limited in the capacity to cover the entire phenotypic landscape (Persad *et al.*, 2023), which is partially due to the unique challenges of scCAS data, including high dimensionality, extreme sparsity, and near-binary nature, compared to scRNA-seq data (Gao *et al.*, 2023). Although SEACells can be utilized for analyzing scCAS data, it is contingent upon memory-intensive preprocessing of fragment files and employs time-consuming kernel archetypal analysis for identifying metacells. Besides, Scarf was developed for memory-efficient analysis of large-scale single-cell genomics data, including scRNA-seq, CITE-seq, and scCAS data (Dhapola *et al.*, 2022), while still demanding a significant amount of time when processing data, clustering, and saving results to a specified file format on atlas-scale scCAS datasets. Therefore, methods specifically developed for scCAS data and capable of striking a balance between effective downstream cell-type identification, memory usage, and computational efficiency are in pressing need.

To fill this gap, we present a Python package named EpiCarousel that allows for memory- and time-efficient identification of metacells for atlas-level scCAS data. EpiCarousel enables efficient large-scale scCAS data loading via lazy loading techniques, identifies metacells based on graph-based community detection methods, and implements parallel computing in chunks to accelerate computation, circumventing the Python global interpreter lock (GIL) to fully utilize CPU resources. We show that EpiCarousel is sufficient to analyze the atlas-level dataset with over 700 thousand cells and 1 million peaks using efficient RAM consumption (<75 GB) within 2 h, enabling users to analyze large-scale datasets on low-cost devices. Besides, the output metacell-by-region matrix can be seamlessly integrated into the widely used scCAS data analysis pipelines, facilitating flexible and in-depth investigation of scCAS data.

## 2 Methods

**Figure 1.**
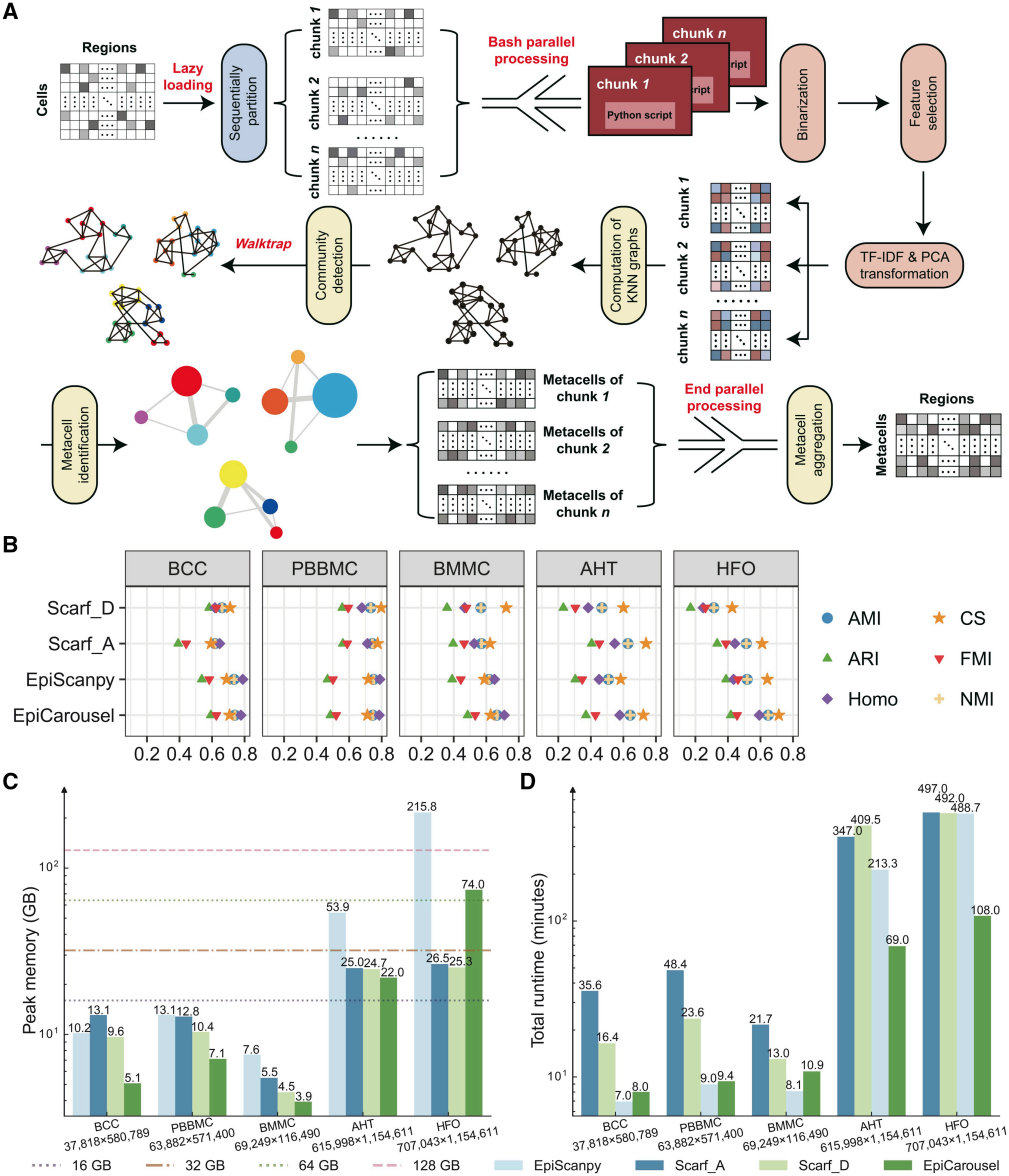
A graphical illustration of EpiCarousel and benchmarking results of various methods. (A) Schematics of EpiCarousel workflow. (B) Clustering performance of different methods across various datasets. (C) Plot of the peak memory usage of each method for benchmarking on various datasets. (D) Plot of total runtime of each method for benchmarking on various datasets. Note that in both (C) and (D), the running processes of Scarf_A and Scarf_D only include the inherent Leiden clustering in Scarf, while EpiScanpy and EpiCarousel utilize four clustering strategies (Dleiden, Dlouvain, Cleiden, and Clouvain). The numbers below the *x*-axis of (C) and (D) represent the number of cells and the number of regions of the corresponding dataset.

Given a scCAS data count matrix stored in the compressed sparse row format, EpiCarousel identifies metacells and generates a metacell-by-region/peak matrix. As shown in Fig. 1A, EpiCarousel first loads the scCAS dataset and partitions it into multiple chunks, then performs data preprocessing and identifies metacells for each chunk in parallel, and finally combines the metacells derived from each chunk to facilitate diverse downstream analyses such as cell clustering, gene regulatory network inference, expression enrichment analysis, partitioned heritability analysis, pathway enrichment analysis, and trajectory inference.

Specifically, EpiCarousel takes an h5ad file, which is a widely used formatted hdf5 file (Wolf *et al.* 2018, Danese *et al.* 2021), as input data. EpiCarousel enables rapid reading of the h5ad file, which may contain massive cells and regions, with minimal memory usage based on lazy loading techniques (Zhang, *et al.* 2024). Let $X\in R^{n\times p}$ denote the raw cell-by-region scCAS data count matrix. EpiCarousel has the following key parameters: (1) the chunk size c, namely the number of cells in each chunk; (2) the number of parallel processes m; and (3) the resolution γ, namely the ratio of the number of cells to that of metacells. Details of the parameters are described in Supplementary Text S1. Then, we sequentially partition X into segments of size c while enabling caching for X to expedite the recurrent access and store these segments as individual h5ad files.

Next, EpiCarousel performs data preprocessing and infers metacells on individual chunks in parallel. We implemented a Bash script to regulate the concurrency level on multi-processor machines, thereby bypassing challenges posed by the GIL of Python that hinder a significant portion of the parallelism potential. For each chunk i, where $i=1,2,\ldots ,\left\lceil n/c\right.\rceil$, we perform data transformation on its cell-by-region matrix $X_{i}\in R^{c\times p}$ to ensure resilience against the inherent sparsity and noise. Initially, we apply binarization to $X_{i}$ as suggested in a recent study (Chen *et al.* 2019), select regions that are accessible in more than 0.01 × c cells (Chen *et al.* 2021, 2022), and perform the term frequency-inverse document frequency (TF-IDF) transformation (Li *et al.* 2021, Chen *et al.* 2023, Tang *et al.* 2024) (Supplementary Text S2). Then, we perform principal component analysis (PCA) to reduce the dimension of cells to 50 (by default), compute the Euclidean distance between cells based on the obtained cell embeddings, construct a graph of k-nearest neighbors, and calculate the connectivity matrix $U_{i}$ among cells as the weighted adjacency matrix.

We identify metacells based on an efficient and reliable community detection algorithm, named *Walktrap* (Pons and Latapy 2005). We assume that the weighted graph $G_{i}$ is connected and consider a discrete diffusion process on $G_{i}$. The transition probability from cell a to cell b at each step is defined as $P_{i_{ab}}=U_{i_{ab}}/d_{i}\left(a\right)$, where $d_{i}\left(a\right)=\sum_{b}U_{i_{ab}}$. The transition matrix $P_{i}$ can be accordingly derived as $P_{i}={V_{i}}^{-1}U_{i}$, where $V_{i_{aa}}=d_{i}\left(a\right),\forall a$ and $V_{i_{ab}}=0$ for a≠b. Let $P_{i_{ab}}^{l}$denote the probability of reaching cell b from cell a by a random walk of length l. Then the distance between cells a and b can be defined as follows:

$$r_{i_{ab}}=\sqrt{\sum_{k=1}^{c}\frac{{\left({P_{i}}_{ak}^{l}-{P_{i}}_{bk}^{l}\right)}^{2}}{d_{i}\left(k\right)}}={\left|\left|{V_{i}}^{-\frac{1}{2}}{P_{i}}_{a\cdot }^{l}-{V_{i}}^{-\frac{1}{2}}{P_{i}}_{b\cdot }^{l}\right|\right|}_{2},$$

where $||\cdot |{|}_{2}$ is the L2 norm of $R^{n}$. We define the probability of reaching cell b from community Y in l steps as ${P_{i}}_{Yb}^{l}=\frac{1}{\left|Y\right|}\sum_{a\in Y}{P_{i}}_{ab}^{l}$. Then the distance between any two communities $Y_{1}$ and $Y_{2}$ can be defined as

$${r_{i}}_{Y_{1}Y_{2}}={\left|\left|{V_{i}}^{-\frac{1}{2}}{P_{i}}_{Y_{1}\cdot }^{l}-{V_{i}}^{-\frac{1}{2}}{P_{i}}_{Y_{2}\cdot }^{l}\right|\right|}_{2}=\sqrt{\sum_{k=1}^{c}\frac{{\left({P_{i}}_{Y_{1}k}^{l}-{P_{i}}_{Y_{2}k}^{l}\right)}^{2}}{d_{i}\left(k\right)}},$$

which can be applied to assess the resemblance among structures. At each step k, for any adjacent communities $Y_{1}$ and $Y_{2}$, let $Y_{3}=Y_{1}\cup Y_{2}$ and merge the two communities that minimize the following expression:

$$\Delta \sigma_{i}\left(Y_{1},Y_{2}\right)=\frac{1}{c}\left(\sum_{a\in Y_{3}}{r_{i}}_{aY_{3}}^{2}-\sum_{a\in Y_{1}}{r_{i}}_{aY_{1}}^{2}-\sum_{a\in Y_{2}}{r_{i}}_{aY_{2}}^{2}\right).$$

The cells residing in the same community are considered to be homogenous and integrated to yield a metacell-by-region matrix by averaging the raw cell-by-region matrices of the cells. Subsequently, metacells of each chunk are saved as h5ad files separately.

Ultimately, EpiCarousel sequentially loads and concatenates the metacells from each chunk to generate a complete metacell-by-region matrix, facilitating seamless integration with conventional scCAS data analysis workflows. Notably, the identifiers of the original cells contained within each metacell are stored in the “obs” field of the output AnnData, which empowers users to effortlessly evaluate the profound impact of metacells on downstream analysis, effectively harnessing annotations from the original scCAS data while consuming minimal memory and time.

The performance of EpiCarousel was evaluated in two aspects. On one hand, we assessed the efficacy of metacells in the essential downstream analyses of clustering and visualization, as suggested in a recent benchmarking study (Chen *et al.* 2019). For clustering, we first binarized the metacell-by-region matrix and performed feature selection. Decimals such as 0.2 will be treated as 1 for subsequent processing. We defined the sparsity of the raw data, denoted as $s_{o}$, as the proportion of non-zero elements in X. Similarly, we defined the sparsity of the obtained metacell matrix as $s_{mc}$ and selected the regions that are accessible in more than $0.01\times s_{mc}/s_{o}$ of the metacells. Next, we performed TF-IDF transformation, PCA, and Louvain and Leiden clustering with the default clustering resolution (denoted as Dlouvain and Dleiden) and the resolution adaptively determined by binary search strategy (denoted as Clouvain and Cleiden; Supplementary Text S3). The obtained clustering label of each metacell is assigned as the clustering result of each cell contained in the metacell. We assessed cell clustering results by six widely used metrics: adjusted mutual information, adjusted Rand index, normalized mutual information, homogeneity score (Homo), completeness score, and Fowlkes-Mallows index (Supplementary Text S4). We also visualized the original cells and the identified metacells via uniform manifold approximation and projection (UMAP). The type of each metacell is annotated as the most dominant cell type among the comprising cells. On the other hand, we evaluated the computational performance by recording the runtime and peak memory usage of all the benchmark experiments, which were conducted on an Ubuntu 22.04 LTS machine with two Intel Xeon Platinum 8375C CPUs and 256GB of RAM.

## 3 Results

We collected five scCAS datasets for extensive evaluation, including the BCC dataset of human basal cell carcinoma tumor microenvironment profiled by scATAC-seq, the PBBMC dataset of human peripheral blood and bone marrow cells profiled by scATAC-seq, the BMMC dataset of human bone marrow mononuclear cells profiled by 10X Multiome, the AHT dataset of 30 adult human tissues profiled by sci-ATAC-seq, and the HFO dataset of 15 human fetal organs profiled by sci-ATAC-seq3. These five datasets were generated by distinct protocols, with varying sizes, dimensions, and numbers of cell types (Supplementary Text S5 and Table S1).

We compared the performance of EpiCarousel with EpiScanpy, a widely used package for scCAS data analysis, Scarf, a memory-efficient analysis Python workflow for large-scale single-cell genomics data, and SEACells, the state-of-the-art method for scCAS metacell identification. To ensure an unbiased and extensive comparison, we conducted benchmark experiments from two perspectives: (i) we followed the tutorials provided by the baseline methods using their default parameter settings, resulting EpiScanpy, Scarf_D, SEACells_D_re75, EpiCarousel and EpiCarousel_re10 (which is identical to EpiCarousel, indicating the metacell resolution of 10); and (ii) we adjusted certain parameters in the baseline methods, such as the number of selected features, the metacell resolution and the number of parallels, for more extensive comparison, resulting Scarf_A (with the batch size adjusted from 1000 to 10 000, the number of threads adjusted from 4 to 8, and the number of selected features adjusted to that of the EpiCarousel-identified metacells), SEACells_D_re10 (with the metacell resolution adjusted from 75 to 10), SEACells_A_re75 (without filtering cells specially when processing fragments), and SEACells_A_re10 (without filtering cells specially and with the metacell resolution adjusted to 10). We also compared the performance of EpiCarousel with the recently launched “sketch” algorithm in Seurat V5 (Hao *et al.* 2024), which aims to enhance the computational scalability of scRNA-seq data.

As illustrated in Fig. 1B and Supplementary Fig. S1A, EpiCarousel achieved the overall best clustering performance across five datasets and six evaluation metrics. The advantage of EpiCarousel becomes increasingly conspicuous as the data size and dimension increase, which is evident in the BMMC, AHT, and HFO datasets. Interestingly, EpiCarousel provided comparable and even superior performance to EpiScanpy, regardless of the clustering strategies, highlighting the capability of our method to identify metacells while preserving cell heterogeneity. Besides, we found that adjusting the parameters of Scarf to be the same as EpiCarousel can improve the performance of Scarf, especially on atlas-level datasets such as AHT and HFO. Note that Scarf only inherently supports Dleiden clustering and cannot provide results of other clustering strategies. For the comparison with SEACells, we failed to collect the fragment data required by SEACells on BMMC, encountered memory errors (exceeded 256GB) on both AHT and HFO when running SEACells, and failed to perform SEACells_D_re10 and SEACells_A_re10 within 96 h on PBBMC. EpiCarousel_re10 and EpiCarousel_re75 consistently outperformed the various settings of SEACells on BCC (Supplementary Fig. S2), as well as on PBBMC (Supplementary Fig. S3). EpiCarousel and SEACells identify a comparable number of metacells when using identical resolutions (Supplementary Table S2). EpiCarousel-identified metacells with higher purity compared to SEACells, while the variance within metacells and the separation between metacells were similar to those identified by SEACells (Supplementary Texts S6, S7 and Figs. S4, S5). We also depicted that EpiCarousel achieves superior clustering performance for large-scale scCAS data compared to the sketch-based analysis (Supplementary Text S8 and Fig. S6A).

Furthermore, we also demonstrated that UMAP visualization of the metacells identified by EpiCarousel can effectively maintain the epigenomic heterogeneity of the massive raw scCAS data (Supplementary Figs. S7 and S8), indicating the broad prospects of the metacells identified by EpiCarousel. We also located metacells on the UMAP visualization alongside individual single cells and evaluated the rationality of metacell assignments identified by EpiCarousel both qualitatively and quantitatively (Supplementary Text S9 and Figs S9–S20). In addition, the metacells identified by EpiCarousel can also play a role in a series of downstream analyses, including identifying new cell types, rare cell types, and sub-cell types (Supplementary Text S10, Figs S21–S26, and Table S3), inferring gene regulatory networks via DeepTFni (Li *et al.* 2022) (Supplementary Text S11 and Figs S27, S28), expression enrichment analysis using SNPsea (Slowikowski *et al.* 2014) (Supplementary Text S11 and Fig.S29), partitioned heritability analysis with LDSC (Finucane *et al.* 2015) (Supplementary Text S11 and Fig. S30A), pathway enrichment analysis through GREAT (McLean *et al.* 2010) analysis(Supplementary Text S11 and Tables S4, S5), and trajectory inference via Slingshot (Street *et al.* 2018) (Supplementary Text S11 and Fig. S30B, C), thus demonstrating that the metacells identified by EpiCarousel can unveil biological insights.

For memory usage and computational efficiency, we observed that EpiCarousel consumed the overall least peak memory (Fig. 1C). Note that all benchmarking experiments included the step of saving results to h5ad files for downstream usage, which explains why Scarf consumed more peak memory on HFO than mentioned in (Dhapola *et al.* 2022). SEACells also occupied significantly more memory than other methods on BCC and PBBMC (Supplementary Fig. S31). We also demonstrated that the memory efficiency of EpiCarousel did not come at the cost of a longer running time. As shown in Fig. 1D, the runtime of EpiCarousel on all five datasets was much lower than that of Scarf, providing significant advantages on the two atlas-level AHT and HFO datasets. EpiCarousel also achieved superior efficiency than SEACells on BCC and PBBMC (Supplementary Fig. S32). Additionally, EpiCarousel requires significantly less peak memory across various datasets compared to the “sketch” algorithm (Supplementary Text S8 and Fig. S6B).

Moreover, we demonstrated that EpiCarousel is robust to noise, chunk size, and resolution. To introduce different levels of noise, we randomly set a certain proportion (ranging from 0% to 90% by a step of 5%) of non-zero elements in the count matrix of the moderate-sized BMMC dataset to zero. In addition, we set the chunk size from 1000 to 20 000 by a step of 1000 and the metacell resolution from 10 to 100 with an increment of 5 for each step. As shown in Supplementary Figs S33–S35, we verified that EpiCarousel consistently outperformed EpiScanpy, across four clustering strategies. Besides, we subsampled the cells in HFO in sequential order and demonstrated the changes in peak memory consumption and runtime of EpiCarousel as the number of cells increased from 10 000 to 100 000 (Supplementary Fig. S1B, C). The results suggest that EpiCarousel also has memory efficiency advantages on moderately large scCAS datasets.

Finally, we examined the potential limitations and effectiveness of our approach to partition the complete dataset into chunks (Supplementary Texts S12, S13 and Figs S36–S49), as well as the feature selection (Supplementary Text S14 and Fig. S50) and clustering methods employed in identifying metacells (Supplementary Text S15, Figs S51–S54, and Table S6) with EpiCarousel.

## 4 Conclusion

We propose EpiCarousel, a memory- and time-efficient metacell identification tool for robustly processing atlas-level scCAS data while preserving epigenomic cell heterogeneity. We anticipate that EpiCarousel, which can be seamlessly integrated with existing workflows, will provide valuable assistance for the investigation of the epigenomic landscape in cells.

## Supplementary information

Supplementary data are available at *Bioinformatics* online.

## Supplementary Material

btae191_Supplementary_Data

## Data Availability

The data underlying this article are available in the article and in its online supplementary material.
