# Supplementary material for: EpiCarousel: memory- and time-efficient identification of metacells for atlas-level single-cell chromatin accessibility data
Source: Bioinformatics. 2024 Apr 8;40(4):btae191. doi: 10.1093/bioinformatics/btae191 (PMC11037479; doi:10.1093/bioinformatics/btae191)
Supplement: btae191_Supplementary_Data [file btae191_supplementary_data.pdf]

Supplementary Information

EpiCarousel: memory- and time-efficient identification of metacells for atlas-level single-cell chromatin accessibility data

Sijie Li[1], Yuxi Li[1], Yu Sun[2], Yaru Li[2], Xiaoyang Chen[3], Songming Tang[1] and Shengquan Chen[1,*]

[1]School of Mathematical Sciences and LPMC, Nankai University, Tianjin 300071, China

[2]Institute of Health Service and Transfusion Medicine, Beijing 100850, China

[3]MOE Key Laboratory of Bioinformatics and Bioinformatics Division of BNRIST, Department of Automation, Tsinghua University, Beijing 100084, China

* To whom correspondence should be addressed.

Contact: chenshengquan@nankai.edu.cn

Contents

Supplementary Texts 6

Text S1. Parameters of EpiCarousel 6

Text S2. TF-IDF transformation 7

Text S3. Clustering methods for evaluation 8

Text S4. Evaluation metrics for clustering 9

Text S5. Data collection and preprocessing 11

Text S6. Evaluation of metacells through purity 12

Text S7. Evaluation of metacells through compactness and separation ... 13

Text S8. Benchmarking against the sketch-based analysis in Seurat V5 .. 15

Text S9. The rationality of metacell assignments by EpiCarousel 17

Text S10. EpiCarousel enables detection of new cell types, rare cell types, and sub-cell types 19

Text S11. EpiCarousel facilitates a series of biological downstream analyses 21

Text S12. Potential limitations and effectiveness of the approach of partitioning the whole dataset 26

Text S13. Benchmarking results of various methods on chunks of datasets 29

Text S14. Discussion on the approach of feature selection and its effectiveness in EpiCarousel 31

Text S15. Motivation and advantages of implementing Walktrap for metacell identification 33

Supplementary Figures 35

Fig. S1 35

Fig. S2 36

Fig. S3 37

Fig. S4 38

Fig. S5 39

Fig. S6 40

Fig. S7 41

Fig. S8 42

Fig. S9 43

Fig. S10 44

Fig. S11 45

Fig. S12 46

Fig. S13 47

Fig. S14 48

Fig. S15 49

Fig. S16 50

Fig. S17 51

Fig. S18 52

Fig. S19 53

Fig. S20 54

Fig. S21 55

Fig. S22 56

Fig. S23 57

Fig. S24 58

Fig. S25 59

Fig. S26 60

Fig. S27 61

Fig. S28 62

Fig. S29 63

Fig. S30 64

Fig. S31 65

Fig. S32 66

Fig. S33 67

Fig. S34 68

Fig. S35 69

Fig. S36 70

Fig. S37 71

Fig. S38 72

Fig. S39 73

Fig. S40 74

Fig. S41 75

Fig. S42 76

Fig. S43 77

Fig. S44 78

Fig. S45 79

Fig. S46 80

Fig. S47 81

Fig. S48 82

Fig. S49 83

Fig. S50 84

Fig. S51 85

Fig. S52 86

Fig. S53 87

Fig. S54 88

Supplementary Tables 89

Table S1. Summary of the 5 scCAS datasets for benchmarking 89

Table S2. The number of metacells identified by each method in the comparison on five datasets 90

Table S3. The number and proportion of cells of each cell type in the BCC dataset 91

Table S4. Identified significant pathways from classical dendritic metacells via the GREAT analysis 92

Table S5. Identified significant pathways from megakaryocyte-erythroid progenitor (MEP) metacells via the GREAT analysis 93

Table S6. The number of metacells identified by various clustering methods on four datasets, respectively 94

Table S6 (continue). The number of metacells identified by various clustering methods on four datasets, respectively 95

References 96

Supplementary Texts

Text S1. Parameters of EpiCarousel

EpiCarousel has the following key parameters: (1) the chunk size c, which denotes the number of cells in each chunk (default: 10,000); (2) the number of parallel processes m (default: 8); (3) preprocessing parameters, including whether to binarize **X**, the criterion for peak selection, methods for data transformation and dimension reduction; and (4) the resolution γ (default: 10), defined as the ratio of the number of original cells to that of metacells.

EpiCarousel also includes the following revisable hyperparameters. The if_bi (default: 1) controls whether to make the count matrix **X** a binary matrix. During preprocessing, we select certain peaks/regions that are accessible in at least $filter\_rate$ (default: 0.01) proportion of cells in total. The dimensionality reduction method $decomposition$ (default: $'pca'$) and the number of components for low-dimensional representation $n\_components$ (default: 50) are also adjustable. The mode of constructing the metacell matrix is denoted as mc_mode, which can be set to $'average'$ (by default) or $'sum'$. For the continuous metacell matrix generated, the parameter if_mc_bi (default:1) determined whether to binary the matrix, i.e.

$$x'_{ij} = \begin{cases} 1, \; x_{ij} > threshold \\ 0, \; else \end{cases},$$

where $threshold$ is set to 0 by default.

Text S2. TF-IDF transformation

Recent search has documented that peaks/regions with low occurrence frequencies tend to be able to distinguish cell types (Chen, et al., 2021). To normalize the count matrix $\mathbf{X} \in \mathbb{R}^{n\times m}$ and amplify the weights of infrequently occurring peaks/regions, we apply term frequency-inverse document frequency (TF-IDF) transformation to **X**. The importance of peak j for cell i can be represented as

$$x'_{ij} = \frac{x_{ij}}{\sum_{j=1}^{m} x_{ij}} log\left(\frac{n}{\sum_{i=1}^{n} x_{ij}}\right),$$

and is subsequently normalized as a recent study for scCAS data (Li, et al., 2021) by:

$$v_{ij} = \frac{x'_{ij}}{\sqrt{\sum_{j=1}^{m} {x'_{ij}}^2}}.$$

Text S3. Clustering methods for evaluation

We implemented two configurations of Louvain (Blondel, et al., 2008) and Leiden (Levine, et al., 2015; Traag, et al., 2019) clustering respectively for evaluation of benchmarking methods. The two clustering approaches with default settings are referred to as Dleiden and Dlouvain, respectively. Considering the impact of the number of identified clusters, we also adopted the binary search to obtain clustering results that closely approximate the number of ground truth cell types. These two settings are denoted as Cleiden and Clouvain, respectively.

Text S4. Evaluation metrics for clustering

We assessed the clustering results for all the benchmarking methods based on the Adjusted Mutual Information (AMI), the Adjusted Rand Index (ARI), the normalized mutual information (NMI), the homogeneity score (Homo), the completeness score (CS), and the Fowlkes-Mallows index (FMI).

Given the high imbalance of cell types and the presence of rare cell types in our benchmark datasets, we utilized the Adjusted Mutual Information (AMI) to evaluate the clustering results, as recommended in (Romano, et al., 2016). In addition, we also employed the Adjusted Rand Index (ARI) for there are some cell types of similar size (Romano, et al., 2016).

Assuming T and P are the true labels and predicted clustering assignments of cells, respectively. Let a_i denote the number of cells belonging to the i-th cell type in the true cell type sets T, b_j represent the number of cells belonging to the j-th clustering label, n denotes the number of cells in the dataset, n_{ij} indicates the count of cells belonging to both the i-th true cell type and the j-th clustering label, $H(\cdot)$ represents the entropy function and E denotes the expectation function. The Mutual Information (MI) provides a measure of agreement between two sets of labels while disregarding label permutations. AMI, a more recently introduced metric, is normalized against chance compared to MI. NMI is commonly employed in research. The calculations for each of them are as follows:

$$MI(P,T) = \sum_{i=1}^{|T|}\sum_{j=1}^{|P|}\frac{|T_i \cap P_j|}{n} log\left(\frac{n|T_i \cap P_j|}{|T_i||P_j|}\right),$$

$$NMI(P,T) = \frac{MI(P,T)}{mean\big(H(T),H(P)\big)},$$

$$AMI = \frac{MI - E[MI]}{mean\big(H(T),H(P)\big) - E[MI]}.$$

The Rand index (RI) quantifies the similarity between the two assignments, disregarding any variations in ordering. ARI is adjusted for RI. It corrects for chance and is calculated as follows:

$$ARI = \frac{\sum_{ij}\binom{n_{ij}}{2} - \left[\sum_i\binom{a_i}{2}\sum_j\binom{b_j}{2}\right]/\binom{n}{2}}{\frac{1}{2}\left[\sum_i\binom{a_i}{2} + \sum_j\binom{b_j}{2}\right] - \left[\sum_i\binom{a_i}{2}\sum_j\binom{b_j}{2}\right]/\binom{n}{2}}.$$

Homogeneity (Homo) stands for whether each cluster consists of cells belonging to a singular true cell type. On the other side, completeness (CS) stands for whether all the cells of a singular

true cell type are assigned to the identical cluster in P. Both of the two metrics indicate better clustering performance if they are higher. Homo and CS are calculated as follows:

$$Homo = 1 - \frac{H(T|P)}{H(T)},$$

$$CS = 1 - \frac{H(P|T)}{H(P)}.$$

The Fowlkes-Mallows index (FMI) measures similarity between two assignments and is calculated as follows:

$$FMI = \frac{TP}{\sqrt{(TP + FP)(TP + FN)}}$$

where TP denotes the number of pair of cells that belong to the same clusters in both T and P, FP denotes the number of pair of cells that belong to the same clusters in T and not in P, FN denotes the number of pair of cells that belong to the same clusters in P and not in T.

Text S5. Data collection and preprocessing

For comprehensive and systematic assessment, we collected five human single-cell chromatin accessibility sequencing (scCAS) datasets that were generated using four different protocols and encompassed varying cell counts, number of peaks/regions, number of distinct cell types, proportions of major cell types, degree of cell type imbalance, and levels of sparsity.

First, we collected scATAC-seq profiles from seven patients diagnosed with basal cell carcinoma (BCC) to investigate the tumor microenvironment (TME). Cell types of scATAC-seq clusters were classified using neighboring genes to cluster-specific *cis*-elements. This dataset containing the BCC TME cells was referred to as BCC (Satpathy, et al., 2019). Aside from BCC, we also collected 63,882 scATAC-seq profiles with 31 cell types of human hematopoietic cells from 16 healthy individuals and referred as PBBMC (Satpathy, et al., 2019). The cell types of PBBMC were annotated identically to BCC. To assess the performance of various methods across different ages, genders, races, and blood types, we collected a multi-lab dataset of human bone marrow mononuclear cells from multiple countries. This dataset, referred to as BMMC, was annotated with cell types and states using marker genes derived from literature, data, and expert curation. To further evaluate the scalability of these methods, we collected two additional datasets and referred as AHT (Zhang, et al., 2021) and HFO (Domcke, et al., 2020), respectively. The AHT dataset contained 615,998 cells of 111 different cell types from 30 adult human donors, while the HFO dataset comprised approximately 800,000 cells from 59 human fetal samples. Note that HFO underwent the same quality control as AHT (Zhang, et al., 2021), which is why the AHT dataset we used differs slightly from the one mentioned in (Domcke, et al., 2020).

A summary of the five collected datasets is presented in Supplementary Table S1. The imbalance degree of each dataset was calculated using the following formula:

$$I = 1 + \frac{1}{logC}\sum_{c=1}^{C}\frac{n_c}{N}log\frac{n_c}{N}.$$

Here, C represents the count of unique cell types in the dataset, n_c denotes the number of cells belonging to the c-th cell type, and N represents the total number of cells in the dataset.

To mitigate the potential bias introduced by the order of cell arrangement in algorithms employing blocking techniques, all experiments were conducted using the aforementioned dataset with a randomized cell order.

Text S6. Evaluation of metacells through purity

Given that the AMI or ARI etc will be affected by the clustering procedure and clustering parameters, we define the purity of a metacell as the proportion of the most represented cell type among the constituent single cells, consistent with the definition of purity in SEACells. Taking the BCC and PBBMC datasets as examples, the results (Fig. S4) demonstrate that EpiCarousel identifies metacells with higher purity compared to SEACells at resolutions of 10 and 75, indicating a greater consistency of cell types within metacells and better characterization of cellular heterogeneity.

Text S7. Evaluation of metacells through compactness and separation

Cell heterogeneity or metacell purity is not the only way to deem a good metacell. We have undertaken several efforts to evaluate the metacells identified.

Firstly, we introduced two metrics, compactness and separation, to assess the variance within metacells and the degree of separation between metacells, respectively, following SEACells. Specifically, for each metacell, the variance in each diffusion component (DC) dimension is calculated across the cells comprising that metacell. The compactness is reported as the average variance across components and for a metacell m, the compactness score $Compactness(m)$ is computed as follows.

$$Compactness(m) = \frac{1}{d}\sum_{i=1}^{d} variance_{cells \in m}(DC_i),$$

where DC_i denotes the ith DC, and d represents the number of DCs, with default settings following SEACells. Lower compactness values imply greater homogeneity among the individual cells constituting the metacell.

We denote the methods for computing diffusion components from the low-dimensional representation of single-cell PCA obtained through the EpiScanpy analysis pipeline as EpiCarousel_re10 and EpiCarousel_re75, corresponding to EpiCarousel resolutions of 10 and 75, respectively. Additionally, we utilized the 'epicarousel.preprocess.ATAC_preprocess()' function from the EpiCarousel pipeline to preprocess the entire scCAS dataset. This involved binarizing the count matrix, feature selection, TF-IDF normalization, and PCA to obtain the low-dimensional representation, X_pca, of single-cell data. From the embedding X_pca, we computed the cell diffusion components. We denote these methods with resolutions 10 and 75 as EpiCarousel_A_re10 and EpiCarousel_A_re75, respectively. In the case of SEACells, we utilize its corresponding preprocessing to obtain the low-dimensional representation X_svd of single-cell data for computing cell DCs. Then we obtained the compactness scores of metacells identified by various settings of EpiCarousel and SEACells on the BCC (Fig, S5A) and PBBMC (Fig. S5B) datasets. The results suggest that, except for a few outliers, metacells identified by EpiCarousel and SEACells exhibit similar low compactness scores, indicating minimal variance within metacells for both approaches.

To investigate how well separated the metacells are from each other, we calculated the separation score as reported in SEACells. The diffusion embedding of each metacell is derived

as the average embedding of the constituent single cells. The separation of a metacell is defined as the Euclidean distance in the diffusion embedding between the metacell and its nearest neighboring metacell. Higher separation indicates a greater distinction between metacells. Taking the BCC and PBBMC datasets as examples, the results (Fig. S5C and S5D) demonstrate that metacells identified by both EpiCarousel and SEACells exhibit similar separation, with EpiCarousel showing higher outliers.

Text S8. Benchmarking against the sketch-based analysis in Seurat V5

We compared EpiCarousel with the ‘sketch’ algorithm in Seurat V5 (Hao, et al., 2024), demonstrating its relative practicality. The sketch-based analysis workflow in Seurat V5 is an approach designed for scRNA-seq data to enhance computational efficiency in analyzing large-scale single-cell datasets. We performed the sketch-based analysis in Seurat V5 on all five scCAS datasets, following its tutorial (https://satijalab.org/seurat/articles/seurat5_sketch_analysis). Additionally, we adjusted certain steps of the tutorial to better accommodate the scCAS data, as this method is designed for scRNA-seq data.

We first created a Seurat object from scCAS datasets in the h5ad format and then selected a subset (‘sketch’) of 5,000 cells by default. Then, we perform preprocessing and clustering on the sketched dataset and extend the clustering assignments to the full datasets. Specifically, we designated the settings configured according to the commands and default parameter settings in the sketch-based analysis tutorial as sketch_RNA_D. Since EpiCarousel defaults to identifying metacells as one-tenth of the number of single cells, for a fair comparison, we also conducted an analysis with the sketched dataset size set to one-tenth of the number of single cells, following the commands in the tutorial, denoted as sketch_RNA_A.

Due to the fact that the sketch-based analysis is designed for scRNA-seq data, the preprocessing steps may not be suitable for scCAS data. Therefore, we replaced the method of normalizing single-cell dataset as outlined in the tutorial with ‘RunTFIDF()’, which is a function of Signac (Stuart, et al., 2021). Signac is a commonly used analysis framework designed for single-cell chromatin data, including scCAS data, and it seamlessly integrates with Seurat. We also replaced the preprocessing of the sketched dataset with ‘FindTopFeatures()’, ‘RunSVD()’, and ‘FindNeighbors()’ in the Signac workflow. In particular, the parameter 'reduction' of ‘FindNeighbors()’ was set to ‘lsi’ and ‘dims’ was set to 2:30. The other two functions were left with their default settings, following the tutorial of Signac (https://stuartlab.org/signac/articles/pbmc_vignette). Then we derived four settings of the sketch-based analysis: sketch_ATAC_D_D, sketch_ATAC_D_A, sketch_ATAC_A_D, and sketch_ATAC_A_A. Among these, the third character from the end, ‘D’, signifies sketching a subset of 5,000 cells by default, while ‘A’ represents sketching a subset of 0.1 times the total number of single cells. Among these, the third character from the end, ‘D’, signifies sketching a subset of 5,000 cells by default, while ‘A’ represents sketching a subset with one-tenth of the total number of single cells. The last character, ‘D’, indicates that ‘FindTopFeatures()’ adopts

the default setting min.cutoff=‘q5’ in Signac, which sets the top 95% most common features as the VariableFeatures for the object, while ‘A’ sets the min.cutoff to 0.01 times the total number of single cells to include features with counts greater than one-thousandth of the number of single cells, following the feature selection method similar to EpiCarousel. To evaluate its performance, we utilized ‘FindClusters()’ according to the sketch-based analysis tutorial to perform Louvain clustering and Leiden clustering with the resolution setting of 1 (which is consistent with the parameters used for evaluating EpiCarousel clustering performance), denoted as Dlouvain and Dleiden, respectively.

We conducted all the experiments on an Ubuntu 22.04 LTS machine with two Intel Xeon Platinum 8375C CPUs and 256GB of RAM. All six configurations of the ‘sketch’ method encountered errors on the HFO dataset. The sketch_RNA_A encountered errors when running Dleiden on the AHT dataset. Both sketch_ATAC_D_D and sketch_ATAC_A_D encountered memory errors (exceeded 256 GB) on the BCC, PBBMC, and AHT datasets. The sketch_ATAC_D_A and sketch_ATAC_A_A encountered memory errors (exceeded 256 GB) on the AHT dataset. The clustering results (Fig. S6A) indicate that directly applying the ‘sketch’ approach to scCAS data yielded poor results, while switching to preprocessing methods suitable for scCAS data significantly improved the clustering performance on the scCAS dataset. EpiCarousel exhibits the advantage of cellular heterogeneity characterization compared to the sketch-based analysis in Seurat V5. Additionally, the peak memory required for each method (Fig. S6B) demonstrates the high computational efficiency of EpiCarousel when analyzing large-scale scCAS data.

Text S9. The rationality of metacell assignments by EpiCarousel

In this section, we investigated whether metacells identified by EpiCarousel could cover the entire phenotypic landscape and analyzed the relationship between the distribution, size, and density of metacells and the density of single cells. We computed two types of cell density. The first type of cell density is calculated following SEACells. Computing the distance to the 150th nearest neighbor for each single cell using diffusion components, and then the cell density is calculated as 1 divided by the distance. We preprocessed the entire scCAS dataset using the 'epicarousel.preprocess.ATAC_preprocess()' function from the EpiCarousel pipeline. This preprocessing involved binarizing count matrix, feature selection, TF-IDF normalization, and PCA to obtain the low-dimensional representation X_pca of single-cell data, from which cell diffusion components were calculated. For SEACells, we used the corresponding preprocessing to obtain the low-dimensional representation X_svd of single cells for calculating diffusion components, following its tutorial. Subsequently, we visualized the preprocessed single-cell data using UMAP (Fig. S9A, Fig. S10A, Fig. S11A, Fig. S15A, Fig. S16A, and Fig. S17A), and the density of single cells on the BCC and PBBMC datasets (Fig. S9B, Fig. S10B, Fig. S11B, Fig. S15B, Fig. S16B, and Fig. S17B). To visualize metacells located on the UMAP visualization with individual single cells, we calculated the coordinates of metacells on the single-cell UMAP plot by averaging the UMAP coordinates of the constituent single cells, following SEACells (https://github.com/dpeerlab/SEACells/blob/main/SEACells/plot.py). We utilized single-cell data preprocessed by the EpiCarousel workflow to compute UMAP coordinates, thus obtaining the coordinates of metacells identified by EpiCarousel on the single-cell UMAP plot (Fig. S9C, Fig. S12A, Fig. S15C, and Fig. S18A). For SEACells, we followed its tutorial to obtain the coordinates of the identified metacells on the single-cell UMAP plot (Fig. S10C, Fig. S11C, Fig. S13A, Fig. S14A, Fig. S16C, Fig. S17C). We visualized the density of metacells using this UMAP low-dimensional representation (Fig. S9F, Fig. S10F, Fig. S11F, Fig. S12C, Fig. S13C, Fig. S14C, Fig. S15F, Fig. S16F, Fig. S17F, and Fig. S18C) where metacells identified by EpiCarousel utilized their low-dimensional representation obtained through PCA for density calculation and metacells identified by SEACells utilized their default singular value decomposition (SVD) for density calculation. We also visualized the size of metacells, namely the number of constituent single cells, using this UMAP low-dimensional representation (Fig. S9G, Fig. S10G, Fig. S11G, Fig. S12D, Fig. S13D, Fig. S14D, Fig. S15G, Fig. S16G, Fig. S17G, and Fig. S18D). Additionally, we examined the distribution of metacell sizes for each metacell identification approach at different resolutions on the BCC (Fig. S19) and PBBMC (Fig. S20) datasets to explore the relationship between metacell size and the

resolution of metacell identification. The results indicate that the size of a metacell is not always equal to the resolution of metacell identification and may be smaller or larger than the resolution.

Another more intuitive method for density calculation is to divide the UMAP plot into 100 equal parts, and the number of cells in each part divided by the total number of cells gives the single-cell density for that region (Fig. S9D, Fig. S10D, Fig. S11D, Fig. S15D, Fig. S16D, and Fig. S17D). Similarly, the number of metacells in each part divided by the total number of metacells gives the metacell density for that region (Fig. S9H, Fig. S10H, Fig. S11H, Fig. S12E, Fig. S13E, Fig. S14E, Fig. S15H, Fig. S16H, Fig. S17H, and Fig. S18E). We denote the density calculated in this way as Density2. The above results indicate that both EpiCarousel and SEACells are capable of identifying metacells covering the entire phenotypic landscape. We demonstrated that EpiCarousel-identified metacells can distinguish between different cell states, including four types of tumor cells in the tumor microenvironment, six rare cell types, and two subtypes of therapy-responsive T cells on the BCC dataset (Text S10). This also confirms that EpiCarousel-identified metacells do not suffer from the problem of neglecting certain cell states by aggregating metacells in densely populated single-cell regions. The distribution of metacells identified by EpiCarousel on the single-cell UMAP is not entirely uniform. Some metacells represent those outlier small clusters of single cells, while some metacells cluster and may overlap on the UMAP plot. The density distribution of metacells identified by EpiCarousel is similar to that of single cells but not identical. This is anticipated and plausible since regions enriched with homogeneous individual cells may yield fewer metacells with larger sizes, while areas with heterogeneous single cells may result in the identification of numerous smaller-sized metacells.

In summary, we have demonstrated the rationality of the metacells identified by EpiCarousel in terms of cell density.

Text S10. EpiCarousel enables detection of new cell types, rare cell types, and sub-cell types

We further demonstrated the capability of EpiCarousel to identify novel cell types and preserve rare cell types and sub-cell types at single-cell resolution in this section.

Firstly, we demonstrate how EpiCarousel facilitates the discovery of novel cell types through data visualization. We took the BCC dataset as an example, which is a scCAS dataset from primary tumor biopsies of patients with basal cell carcinoma (BCC). Within the tumor microenvironment (TME), four distinct tumor cell clusters, namely Tumor 1, Tumor 2, Tumor 3, and Tumor 4, were identified (Fig. S7A) at single-cell level. The UMAP visualizations of metacells identified by EpiCarousel (Fig. S21A and S21B) illustrate the capability of EpiCarousel to maintain the discrimination of tumor cell clusters at metacell-level, distinctly separating the four subgroups: Tumor 1, Tumor 2, Tumor 3, and Tumor 4. This provides novel biological insights into the investigation of new cell types within the BCC TME.

Secondly, we illustrated that EpiCarousel is capable of capturing rare cell types. Taking the BCC dataset as an example, this dataset comprises a total of 20 cell types, with the quantity and proportion of each type shown in Table S3. Assuming cell types with proportions less than 2% are considered rare cell types, the following six cell types: Plasma B, Th1, B, NK1, Endothelial, and Myeloid, are considered rare cell types. We assess the ability of EpiCarousel to find rare cell types from both qualitative and quantitative perspectives. Firstly, we assigned cluster labels obtained from Dlouvain clustering to each metacell identified by EpiCarousel, denoting the corresponding label of each constituent single cell as mc_Dlouvain. We identified the predominant mc_Dlouvain label within mc_Dlouvain for each rare cell type, yielding ‘18’, ‘16’, ‘17’, ‘7’, ‘13’, and ‘15’. To investigate the capability of EpiCarousel in maintaining rare cell types at the single-cell resolution, we grouped the 16 cell types excluding these six rare cell types into one cluster and the clusters other than the aforementioned six mc_Dlouvain labels into another cluster, and depicted the corresponding relationship in a 7 vs. 7 manner. The results (Fig. S22) reveal a strong correspondence between the Plasma B cell type and mc_Dlouvain cluster 18, the B cell type and mc_Dlouvain cluster 17, the Endothelial cell type and mc_Dlouvain cluster 13, and the Myeloid cell type and mc_Dlouvain cluster 15. This suggests that despite reducing data resolution, EpiCarousel remains capable of identifying rare cell types that can be found at the single-cell resolution. Additionally, we explored the correspondence between the six mentioned metacell Dlouvain clustering labels and the above six rare cell types

within metacells (Fig. S23), where the cell type of a metacell was assigned based on the predominant cell type of single cells it contains. The results demonstrated a good correspondence between Plasma B, B, Endothelial, and Myeloid types and clusters 18, 17, 13, and 15, respectively, at the metacell level, indicating the ability of EpiCarousel to maintain rare cell types from the original single-cell data at the metacell level. We visualized the metacell data identified by EpiCarousel using UMAP, clearly distinguishing between CD8 TEx and Tfh types within metacell types (Fig. S21C). Secondly, we computed adjusted mutual information (AMI), adjusted Rand index (ARI), normalized mutual information (NMI), homogeneity score (Homo), completeness score (CS), and Fowlkes-Mallows index (FMI) to evaluate the Dlouvain clustering performance of EpiCarousel on the aforementioned six rare cell types (Fig. S24A) quantitatively, based on the mc_Dlouvain labels of these six rare types in the clustering results of the full single-cell dataset. The results demonstrate the potential of EpiCarousel in discovering rare cell types.

Thirdly, we evaluated the ability of EpiCarousel to identify sub-cell types from both qualitative and quantitative perspectives. Taking the BCC dataset as an example, CD8+ exhausted (CD8 TEx) and CD4+ T follicular helper (Tfh) cells represent two subtypes of therapy-responsive T cells (Satpathy, et al., 2019). We identified the predominant mc_Dlouvain labels for CD8 TEx and Tfh cells in single cells as '4' and '2', respectively. Treating the other 18 cell types in single cells as one cluster and the other clusters in mc_Dlouvain as one cluster, we investigated this 3 vs. 3 correspondence (Fig. S25), revealing strong correspondence between CD8 TEx cells in single cells and mc_Dlouvain cluster 4, and between Tfh cells and cluster 2. Similarly, CD8 TEx and Tfh types in metacells also correspond well with Dlouvain clusters 4 and 2, respectively (Fig. S26). We also employed UMAP to visualize the metacell data identified by EpiCarousel. The results indicate clear distinguishment between CD8 TEx and Tfh types within metacell types (Fig. S21D), demonstrating the ability of EpiCarousel to identify sub-cell types that can be found at the single-cell resolution. We also quantitatively assessed the Dlouvain clustering performance of EpiCarousel on CD8 TEx and Tfh using six clustering metrics: AMI, ARI, NMI, Homo, CS, and FMI. Clustering evaluation was performed on subsets belonging to CD8 TEx and Tfh from the complete mc_Dlouvain clustering results of the BCC dataset. The results (Fig. S24B) indicate the potential of EpiCarousel to investigate sub-cell types.

Taken together, the above results shed light on the capability of EpiCarousel in identifying new cell types, rare cell types, and sub-cell types, preserving certain biological implications at the single-cell resolution.

Text S11. EpiCarousel facilitates a series of biological downstream analyses

In this section, we demonstrated the ability of EpiCarousel for biological implications from five new perspectives including building gene regulatory networks.

Firstly, we assessed the performance of EpiCarousel in inferring gene regulatory networks using DeepTFni (Li, et al., 2022). The intricate regulatory relationships among transcription factors (TFs) are typically modeled as TF regulatory networks (TRNs), a vital subset of gene regulatory networks that underlie numerous biological processes and diseases (Li, et al., 2022). We employed DeepTFni to infer transcription factor regulatory networks from the identified metacells by EpiCarousel. Taking the PBBMC dataset as an example, We implemented DeepTFni with default settings for each type of metacell. For Plasma metacells, DeepTFni identified 278 TF genes, which represent a group of TF genes with a high likelihood of regulatory interactions. *PRDM1* has been noted as a critical TF for plasma cell fate in prior research (Satpathy, et al., 2019). Within this group of TF pairs, DeepTFni predicted regulatory interactions between *PRDM1* and 18 other TF genes: *CLOCK*, *CTCF*, *EGR1*, *KLF1*, *KLF15*, *KLF16*, *KLF4*, *MAZ*, *MNT*, *PURA*, *RREB1*, *SP1*, *SP2*, *TBX1*, *TFDP1*, *THAP1*, *VSX2*, and *ZFX*. These results may provide biological insights into the regulatory mechanisms of the critical TF gene *PRDM1* in plasma cells. Previous studies have documented that the TF gene *IRF4* defines plasma cells (Tang, et al., 2021). DeepTFni predicted interactions between *IRF4* and 128 other TFs, including *KLF13*, *FOXO3*, and others. Additionally, DeepTFni predicted interactions between the TF gene *FOSL2* and 39 other TF genes, including *ASCL2*, *FOXM1*, *FOXO1*, *GATA3*, *GLIS2*, *IRF4*, *KLF1*, and *PAX5*, among which the regulatory relationships of *FOSL2* with *FOXO1* and *IRF4* have been reported in the earlier literature (Ubieta, et al., 2017) that FOSL2-encoded TF FRA2 plays a significant role as a key upstream regulator of *FOXO1* and *IRF4* expression, modulating the proliferation and differentiation of B cells across various stages. Given the multitude of predicted TF regulatory relationships, we selectively visualized the top 10 TF genes with the most interactions and the bottom 20 TF genes, along with the previously analyzed *FOSL2*, *FOXO1*, *IRF4*, and *PRDM1* in Fig. S27A, for clarity. The results indicate the capability of EpiCarousel to infer gene regulatory networks from metacells of the Plasma cell type.

Similarly, we performed DeepTFni with default settings on metacells of the Central Memory CD8 T (Tcm) type. Previous studies have indicated that the formation and function of Tcm cells

rely on transcription factors such as TCF1 (also known as TCF7) (Jeannet, et al., 2010; Zhao, et al., 2010), STAT3, and FOXO1(Hess Michelini, et al., 2013; Kim, et al., 2013). The TF gene *PRDM1*, encoding B-lymphocyte-induced maturation protein-1, has been reported in prior literature (Rutishauser, et al., 2009) to suppress the acquisition of central memory T cell properties. Additionally, BCL6 plays a crucial role in the generation and secondary expansion of Tcm (Ichii, et al., 2004). Therefore, we focused on the above five TF genes: *TCF7*, *STAT3*, *FOXO1*, *PRDM1*, and *BCL6*. DeepTFni captured a total of 289 TFs with a high likelihood of regulatory interactions in the Tcm metacells and inferred a regulatory relationship between *FOXO1* and *BCL6*, which may provide extra biological insights into the investigation of *FOXO1* licensing *BCL6* activity, as delineated in prior literature (Dominguez-Sola, et al., 2015). We selected the top 10 TF genes with the most regulatory relationships and the bottom 20 TF genes from the predicted results, along with five TF genes of particular interest, for visualization (Fig. S27B), to demonstrate the capability of the Tcm-type metacell identified by EpiCarousel in inferring gene regulatory networks.

We also investigated the role of EpiCarousel in inferring gene regulatory networks during B-cell differentiation processes. According to prior research (Revilla, et al., 2012), B-cell differentiation during the development of common lymphoid progenitors (CLPs) to Pro-B cells relies on the sequential activity of instructive transcription factors like EBF1, and PAX5. DeepTFni captured regulatory relationships between TF gene *PAX5* and 183 other TF genes including *FOXO1*, *GATA3*, and *GATA6* in CLP metacells, and 173 TF genes in Pro-B metacells, aligning with the above reports and providing significant insights into the regulatory role of the B-cell identity factor PAX5 in controlling the developmental potential of lymphoid progenitors towards the B-cell lineage. We visualized the top 10 TF genes with the most regulatory relationships and the bottom 20 TF genes, along with *PAX5* of interest, from the predicted results of CLP and Pro-B, respectively (Fig. S27C and S27D). The results indicate that the metacells identified by EpiCarousel also play a certain role in inferring gene regulatory networks during cell differentiation.

Moreover, we explored the inference of transcription factor regulatory relationships by EpiCarousel in T-cell subtypes. DeepTFni predicted 268 TF genes with a high likelihood of regulatory interactions in the Th1 metacells of the BCC dataset. Among these TF genes, TBX21, STAT1, STAT4, RUNX3, EOMES, and GATA3, coordinate to trigger the differentiation of Th1 cells (Luckheeram, et al., 2012) and we captured 25, 19, 16, 36, 15, and 25 potential TF gene regulatory relationships of each TF gene, respectively. The results potentially provide novel insights into the differentiation process of Th1 cells. We selectively visualized the top 10 TF

genes with the most regulatory relationships and the bottom 20 TF genes for clarity, along with the aforementioned six TF genes of interest (Fig. S28).

The above results indicate that the metacells identified by EpiCarousel are capable of being used for inferring gene regulatory networks, particularly for the inference of its core subnetwork TRN, and providing insights into the complex regulation process of cell differentiation.

Secondly, we explored the cell type-specific peaks of metacells identified by EpiCarousel for the enrichment of specificity to tissues via SNPsea (Slowikowski, et al., 2014). Specifically, we selected the top 1000 differentially accessible peaks for each type of metacells identified by EpiCarousel on the PBBMC dataset, using the 'episcanpy.tl.rank_features()' function from EpiScanpy on the binary metacell-by-region matrix. We combined the least differentially accessible peaks from each metacell type to form a set of 1000 background peaks. We performed SNPsea analysis with default settings for single nucleotide polymorphisms (SNPs) in each set of the cell type-specific peaks and the set of background peaks, respectively. The enrichments of tissue-specific expression in profiles of 17,581 genes across 79 human tissues (Su, et al., 2004) were quantified. For the PBBMC dataset from human peripheral blood and bone marrow cells, we present the top 30 significantly enriched tissues. It was observed that tissues related to peripheral blood and bone marrow significantly enriched for cell type-specific expression of the genes within the metacell type-specific peaks while showing less enrichment in the background peaks (Fig. S29). For example, we identify Thymus as the tissue with the most significant enrichment (Bonferroni corrected p-value < 0.05) for metacell type-specific peaks in CLP and PB-BDCA4+Dentritic cells as the tissue with the most significant enrichment for metacell type-specific peaks in pDC. Significant enrichment for BM-CD71+Early Erythroid, BM-CD34+, and BM-CD105+Endothelial tissue-specific expression, which is related to bone marrow, is observed in MEP-specific peaks. The above results indicate that the cell type-specific peaks of metacells identified by EpiCarosuel can provide obvious tissue specificity, and thus better capture the cell heterogeneity in related tissues.

Thirdly, we wondered if EpiCarousel can contribute to the research on the variations of the phenotypes, such as diseases, based on the metacell type-specific peaks. More specifically, following the original study (Satpathy, et al., 2019), we created the Immune dataset by selecting 2,129 $CD34^+$ bone marrow progenitors and dendritic metacells across 10 subpopulations (HSC/MPP, MEP, CMP/BMP, LMPP, CLP, Pro-B, GMP, MDP, pDC, cDC) from full metacells of the PBBMC datasets. We first obtained 1000 metacell type-specific peaks for each type and a set of 1000 background peaks in the Immune dataset as described above. We then quantified the enrichment of heritability for Childhood-onset asthma phenotypes within metacell type-

specific peaks for each type and background peaks using partitioned linkage disequilibrium score regression (LDSC) (Finucane, et al., 2015) with default settings. LDSC takes summary statistics from genome-wide association studies (GWASs) as input and estimates the enrichment of heritability in an annotated set of SNPs. We followed the recommended workflow for running LDSC using HapMap3 SNPs and downloaded the summary statistics from https://zenodo.org/records/7768714. As depicted in Fig. S30A, there was a strong enrichment of heritability for childhood-onset asthma in cell type-specific peaks of metacells, aligning with previous literature indicating the relevance of individual immune cell types and whole blood to asthma (Ferreira, et al., 2019). Concretely, the enrichments in the metacell type-specific peaks are at least 1.9 times than that in the background peaks. The above results further support the functional relevance of cell type-specific peaks in metacells and their potential role in regulating the investigated phenotypes, thus shedding light on gene regulatory mechanisms in specific cell types or tissues.

Fourthly, we conducted pathway enrichment analysis on metacells identified by EpiCarousel to further explore whether metacells can retain cell type-specific functional insights at the single-cell level. We used the cell type-specific peaks of metacells in the Immune dataset mentioned above to perform GREAT (McLean, et al., 2010) analysis to identify significant pathways associated with each type of metacells. The top three significant pathways for classical dendritic metacells (Table S4) consist of regulation of 'immune system process', 'positive regulation of immune system process' and 'regulation of immune response', which are consistent with the known functions of classical dendritic cells (cDCs). Similarly, we obtained 'hemostasis', 'blood coagulation', and 'coagulation' pathways for megakaryocyte-erythroid progenitor metacells (Table S5), which are consistent with the previous reports that megakaryocytic-erythroid progenitors (MEPs) differentiate to the cells that generate red blood cells and platelets (Lu, et al., 2018), with platelets playing a critical role in hemostasis (Mussbacher, et al., 2019). The aforementioned results bolster the notion that the cell type-specific peaks of metacells identified by EpiCarousel possess the capability to encapsulate cellular functional information and unveil biological insights.

Finally, we delved into the ability of EpiCarousel to infer cell lineages and pseudotimes using Slingshot (Street, et al., 2018), following previous research (Chen, et al., 2021) on scCAS data. Taking the PBBMC dataset as an example, we performed EpiCarousel with default settings on the full PBBMC dataset. We preprocessed the resulting metacells as described in the main text, which included binarizing the metacell-by-region matrix, feature filtering, TF-IDF transformation, and PCA. Subsequently, we constructed a graph of k-nearest neighbors. To infer

trajectory on 2,129 $CD34^+$ bone marrow progenitors and dendritic metacells across 10 subpopulations (HSC/MPP, MEP, CMP/BMP, LMPP, CLP, Pro-B, GMP, MDP, pDC, cDC) from full metacells of the PBBMC dataset, following the original study (Satpathy, et al., 2019), we implemented Slingshot with default settings, except specifying the starting cluster as HSC/MPP from which lineages will be drawn. The inputs for Slingshot include the low-dimensional embedding of metacells obtained by PCA and the metacell type labels, which are annotated as the major type of the constituent single cells. Slingshot outputted the smooth curves representing the estimated cell lineages, which were then mapped to the low-dimensional UMAP space of the 2,129 metacells for visualization. EpiCarousel with Slingshot deciphers the differential lineage, which is highly consistent with the real hematopoietic differentiation tree (Fig. S30B-C). For example, the inferred differentiation path (HSC/MPP → LMPP → CLP → Pro-B) aligns with the previous study (Satpathy, et al., 2019). The above results suggest that metacells identified by EpiCarousel have the potential to facilitate trajectory inference, yielding insights into capturing cell status and pseudotime.

Taken together, the above results show that EpiCarousel facilitates various biological downstream analysis tasks and demonstrate the biological significance of the metacells identified by EpiCarousel.

Text S12. Potential limitations and effectiveness of the approach of partitioning the whole dataset

There is a probability that the cells in different chunks are more similar to each other than the cells in the same chunk. However, since they are in different chunks, they cannot be assigned into one metacell. Intuitively, the order of cell arrangement may have some impact on the identification of metacells. In this section, we investigated the possible problem of our approach to divide the full dataset into chunks.

In addressing the concerns of potential users regarding the chunking method, we have exerted considerable efforts. First, we analyzed the distribution of each cell type across different chunks and the proportion of each cell type within each chunk to investigate whether the phenomenon that the cells in different chunks are more similar to each other than the cells in the same chunk exists. Secondly, we proposed a metric to assess the extent to which the identification of metacells is influenced by this phenomenon. Thirdly, we performed EpiCarousel on the data with different cell arrangement orders to explore the impact of this chunking approach on identifying metacells. Finally, we explained how we balanced the pros and cons to address this potential issue.

We analyzed the distribution of each cell type across different chunks and the proportion of each cell type within each chunk to investigate whether cells in different chunks exhibit greater similarity than those within the same chunk. Take the BCC and PBBMC datasets as examples, which have 37,818 and 63,882 cells, and 20 and 31 cell types, respectively. Due to the varying sizes of the last chunk in each dataset, often smaller than the chunk size c, the proportion of each cell type assigned to the last chunk is typically smaller than that assigned to other chunks (Fig. S36A and Fig. S37A). The distribution of cell types across chunks is relatively similar (Fig. S36B and Fig. S37B). Cells of the same type indeed may be assigned to different chunks, preventing them from being allocated to the same metacell.

We also evaluated the probability that cells in different chunks are more similar than those within the same chunk. Firstly, we preprocessed the single-cell data in the same manner as the preprocessing of single-cell data for each chunk in EpiCarousel. We transformed all non-zero values in the count matrix to 1, and then filtered the features, retaining only peaks with counts in at least 1% of cells. We subsequently normalized the scCAS data matrix using TF-IDF. Next, we performed PCA to obtain a 50-dimensional embedding, based on which we utilized EpiScanpy to compute a neighborhood graph with the number of nearest neighbors set to the

default value of 15, and the method for computing connectivities set to 'umap'. The resulting connectivities represent the weighted adjacency matrix of the neighborhood graph of single cells.

Let $sc1$, $sc2$, and $sc3$ represent three different single cells. Among them, $sc1$ and $sc2$ belong to the same metacell, while $sc3$ does not. They form triplet $(sc1, sc2, sc3)$, with the total number of triplets denoted as N_s. If the connectivity from $sc1$ to $sc2$ is less than that from $sc1$ to $sc3$, we consider $sc1$ to be more similar to $sc3$ than to $sc2$. We counted the number of such triplet $(sc1, sc2, sc3)$, denoted as n_s, and thus obtained the probability $P_{similarity}$ that the similarity between cells inside and outside the metacells is greater than the similarity between cells inside the metacells. The probability is calculated as follows: $P_{similarity} = n_s/N_s$. For the BCC dataset, $P_{similarity} = 0.000968$, and for PBBMC, $P_{similarity} = 0.000552$. Therefore, we conclude that although similar single cells may be allocated to different chunks, and thus cannot be assigned to the same metacell, the similarity between single cells within the metacells identified by the current chunking method is still very high and acceptable.

Besides, we also calculated the distribution of the sizes of the metacells at two resolutions, 10 and 75, taking the BCC and PBBMC datasets as examples (Figs. S19A, D, S20A, B). The results suggest that the sizes of metacells are not strictly equal to the resolution γ. For less similar cells within the same chunk, EpiCarousel may identify multiple smaller-sized metacells from them, thereby to some extent mitigating potential issues caused by sequential chunking.

Thirdly, we examined the robustness of the chunking method employed by EpiCarousel to the cell ordering in the complete data. Firstly, we would like to explain why we did not strive to ensure that cells of the same type were assigned to the same chunk. Performing preprocessing to ensure that cells of the same type are placed in the same chunk is essentially equivalent to analyzing all cells once, potentially demanding more time and memory usage. We deemed this approach impractical. Considering that the chunking method we employed might benefit when cells are ordered by cell type in the raw data, we initially randomized the raw data for a fair benchmark. All scCAS datasets used in our main text were randomized using a random seed of 1. In this section, we randomly selected seeds 301, 1888, and 3407 to shuffle the data, simulating various arrangements of original single-cell data, and verified that the data shuffled with four random seeds were not exactly the same in their respective cell arrangements. Taking the PBBMC dataset as an example, the distribution of each cell type across different chunks and the proportion of each cell type within each chunk varied under the three additional random shuffling scenarios (Figs. S38-S40). We shuffled all five datasets under four random seed settings and ran the default EpiCarousel pipeline to investigate the influence of the chunking

method on metacell identification when cells are partitioned sequentially. We then evaluated the clustering results by adjusted mutual information (AMI), adjusted Rand index (ARI), normalized mutual information (NMI), homogeneity score (Homo), completeness score (CS), and Fowlkes-Mallows index (FMI). The clustering results (Fig. S41) demonstrate the robustness of EpiCarousel to various cell arrangement orders of scCAS datasets, indicating its effectiveness and robustness in practice.

In summary, the current sequential chunking method not only achieves the identification of internally highly similar metacells but also demonstrates robustness to the cell arrangement order of the original scCAS data. Although it may not guarantee that cells within a certain metacell are always more similar than those inside and outside the metacell, the method we currently employ offers certain advantages in terms of time and memory, which is a critical factor we considered in our deliberations.

Text S13. Benchmarking results of various methods on chunks of datasets

For a fair comparison, we executed each compared method separately on each chunk and evaluated the clustering performance individually on each chunk. To be specific, for SEACells, we initially obtained the barcodes of cells contained in each chunk of the shuffled scCAS data. We then specified the parameter 'validBarcodes' in the ArchR function 'createArrowFiles()' as the barcodes corresponding to the cells in each chunk, in order to preprocess the complete fragments file of the BCC dataset and PBBMC dataset, following the SEACells pipeline (https://github.com/dpeerlab/SEACells/blob/main/notebooks/SEACell_computation.ipynb and https://github.com/dpeerlab/SEACells/blob/main/notebooks/ArchR/ArchR-preprocessing-nfr-peaks.R). Subsequently, we applied different settings of SEACells to the preprocessing results on each chunk, ultimately yielding four SEACells configurations: SEACells_D_re10, SEACells_D_re75, SEACells_A_re10, and SEACells_A_re75. The metacells identified by SEACells in each chunk were then evaluated for clustering according to the methodology described in the main text. We encountered errors when running Scarf on chunks of 10,000 cells with a default batch size of 1,000 and failed to obtain results for each chunk. We performed EpiScanpy and EpiCarousel on each chunk, both with default settings. In summary, we implemented EpiScanpy, two configurations of EpiCarousel, and four configurations of SEACells on individual chunks of the BCC and PBBMC datasets. We executed the default EpiScanpy and EpiCarousel on individual chunks of the BMMC, AHT, and HFO datasets. The Dlouvain clustering performance on each chunk of the BCC and PBBMC datasets (Fig. S42) generally follows the order: EpiScanpy > EpiCarousel_re10 > SEACells_A_re10 and SEACells_D_re10 > SEACells_A_re75, SEACells_D_re75 and EpiCarousel_re75. Similarly, clustering results obtained using Dleiden (Fig. S43) show a similar trend: EpiScanpy > EpiCarousel_re10 > SEACells_A_re10 > SEACells_D_re10 > EpiCarousel_re75 > SEACells_D_re75 and SEACells_A_re75. The clustering results evaluated by Clouvain suggest that the clustering performance generally follows the order: EpiScanpy > EpiCarousel_re10 > EpiCarousel_re75, SEACells_A_re10 and SEACells_D_re10 > SEACells_A_re75 and SEACells_D_re75. The Cleiden clustering results indicate that the clustering performance follows the order generally: EpiScanpy and EpiCarousel_re10 > EpiCarousel_re75 > SEACells_A_re10 > SEACells_D_re10 > SEACells_D_re75 > SEACells_D_re75. The results above collectively exhibit the advantage of EpiCarousel in identifying high-quality metacells for scCAS datasets, in addition to the segmentation of the whole data.

The clustering results obtained by Dlouvain, Dleiden, Clouvain, and Cleiden (Fig. S46-S49, respectively) on the BMMC, AHT, and HFO datasets indicate that EpiCarousel_re10 outperforms EpiScanpy in datasets with a larger number of chunks. The advantage of EpiScanpy on small chunks is expected as it's a widely used approach for single-cell level analysis. However, it loses its edge when facing larger datasets. This also suggests that apart from splitting the full scCAS data into chunks, the built-in preprocessing and Walktrap algorithm for identifying metacells in EpiCarousel also confer certain advantages. Moreover, when confronted with large raw scCAS data, EpiScanpy not only demands high hardware and time requirements but also demonstrates less effectiveness compared to EpiCarousel (Fig. 1B-D, and Fig. S1A). With the increasing scalability of single-cell sequencing technology, the generation of datasets spanning hundreds of thousands of cells is becoming more common. For example, Zhang et al. profiled 615,998 single cells in 30 adult human tissues (Zhang, et al., 2021), and Domcke et al. profiled 790,957 single cells across 53 samples representing fetal tissues (Domcke, et al., 2020). This further emphasized the importance of EpiCarousel in efficiently exploring large datasets and its complementarity with EpiScanpy when analyzing small datasets.

Text S14. Discussion on the approach of feature selection and its effectiveness in EpiCarousel

The whole dataset is split by random since the scCAS data we utilized has been shuffled randomly before analysis. Each chunk has a different selected feature list before normalization and dimension reduction. EpiCarousel supports selecting the size of chunks. Since each user has different hardware conditions, they can choose a larger or smaller chunk size based on their hardware conditions. Additionally, as shown in Fig. S34, we observe fluctuations in clustering performance with an increase in chunk size. Therefore, users can choose the inflection point of chunk size where the clustering performance gain is no longer significant as the ideal chunk size.

In this section, we conducted comprehensive experiments to explore the impact of shared feature selection and shared dimensionality reduction on each chunk for identifying metacells. Specifically, we initially attempted to take the union of the different selected feature lists for each chunk and used it as the features for subsequent normalization and dimension reduction steps on each chunk. We denote this feature selection approach as EpiCarousel_U. Secondly, we attempted feature selection on the entire dataset in the same way as in EpiCarousel and applied the selected feature list to each chunk. We denote this feature selection approach as EpiCarousel_E. Thirdly, we attempted to take the union of features selected individually on each chunk and applied Incremental PCA from the scikit-learn package (https://scikit-learn.org/stable/modules/generated/sklearn.decomposition.IncrementalPCA.html) to perform dimensionality reduction on each chunk in a unified way. We denote this approach as EpiCarousel_I. In these three settings, respectively, chunks shared the same features for normalization and dimension reduction. In EpiCarousel_I, in addition to sharing the same features, each chunk also shares the same dimensionality reduction manner. The results (Fig. S50A) indicate that EpiCarousel_re10, EpiCarousel_U, EpiCarousel_E, and EpiCarousel_I achieve very similar clustering performance, except that EpiCarousel_E performs significantly worse on the BCC dataset. However, the peak memory usage during runtime for EpiCarousel_I,

EpiCarousel_U, and EpiCarousel_E exceeds that of EpiCarousel_re10 (Fig. S50B). We conclude that these three settings for shared feature selection show no significant difference in clustering performance compared to EpiCarousel. Since we prioritize computational performance, we decide on selecting features separately for each chunk.

Text S15. Motivation and advantages of implementing Walktrap for metacell identification

In this section, we began by explaining our motivation for using the Walktrap algorithm to identify metacells. Then, we replaced the method for identifying metacells in EpiCarousel from Walktrap with other clustering methods, including the Louvain clustering, to elaborate on the practical advantages of using the Walktrap algorithm for metacell identification.

Firstly, previous review research on community detection algorithms (Gates, et al., 2016)has shown that compared to clustering methods like the Louvain GJA (Rubinov and Sporns, 2011), an improved version of the original Louvain algorithm (Blondel, et al., 2008), and Infomap (Rosvall and Bergstrom, 2008), Walktrap generally outperforms on sparse matrix and exhibit superiority across diverse conditions such as low within-community connectivity, subgroups of unequal sizes, and small subgroups. Walktrap is also sensitive to small groups and performs better when the communities are relatively small. The above conditions align well with the characteristics of scCAS data, making the use of the Walktrap algorithm to identify metacells a reasonable and well-motivated choice. Furthermore, the communities obtained by Walktrap are more stable within runs, making it an appropriate algorithm for exploring the consistency of networks over time (Gates, et al., 2016). Moreover, the Walktrap algorithm iteratively merges communities until all nodes in the network are merged into one community, resulting in a dendrogram. This facilitates the control of the quantity of identified metacells, which is also one of our motivations for utilizing the Walktrap method.

Secondly, we replaced the metacell identification method in EpiCarousel with various parameter settings of the Louvain and Leiden algorithms, empirically demonstrating the advantage of using the Walktrap algorithm for metacell identification. Specifically, we denote the Louvain and Leiden algorithms implemented in EpiScanpy using default settings as Dlouvain and Dleiden, respectively, with both having a default resolution of 1. Given that higher resolutions result in more and finer-grained clusters, we utilize the ‘getNClusters()’ function in EpiScanpy to ensure that the Louvain and Leiden algorithms can identify a sufficient number of metacells. This function employs a binary search algorithm within a limited number of steps to find the resolution that can achieve the closest number of clusters to the target and performs Louvain or Leiden clustering at that resolution. We specify the target number of clusters as the number of cells divided by the metacell resolution, set the binary search steps to the default value of 20, the minimum value for finding an appropriate resolution to the default of 0, and the maximum values to 3 by default, 100, 200, 300, 3000, and 6000, respectively. We

designate the clustering methods as Louvain or Leiden and denote them as Clouvain_[0,3], Clouvain_[0,100], Clouvain_[0,200], Clouvain_[0,300], Clouvain_[0,3000], Clouvain_[0,6000], Cleiden_[0,3], Cleiden_[0,100], Cleiden_[0,200], Cleiden_[0,300], Cleiden_[0,3000], and Cleiden_[0,6000], correspondingly. The clustering results (Fig. S51-S54) indicate that the Louvain and Leiden algorithms for metacell identification are significantly influenced by the clustering resolution parameter, with lower resolutions tending to yield inferior clustering results. This may be related to lower clustering resolutions, such as the commonly used value of 1, resulting in the identification of too few metacells by the Louvain and Leiden algorithms (Table S6). Although adopting uncommonly high clustering resolutions leads to a sufficient number of metacells identified by Louvain and Leiden, it cannot control the number of metacells stably, which is not in line with our intention. In summary, we demonstrated through a series of experiments that the Walktrap algorithm exhibits certain advantages in identifying metacells, in terms of the heterogeneity and the quantity of identified metacells.

Supplementary Figures

Fig. S1

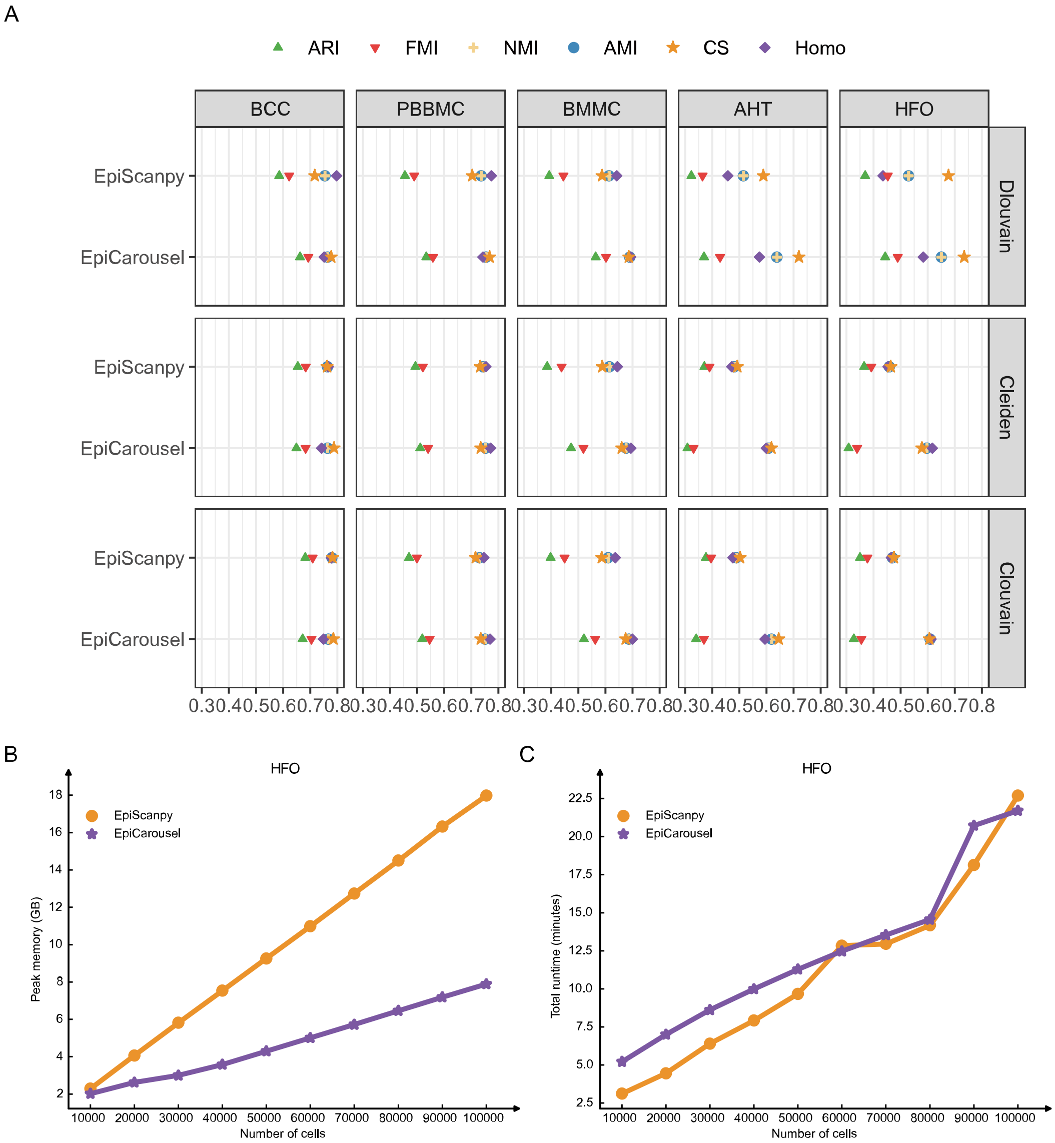

Fig. S1. Plots illustrated the clustering results of Dlouvain, Cleiden, and Clouvain, memory usage, and runtime of EpiCarousel and EpiScanpy. **(A)** Dot plots showing the clustering results of EpiCarousel and EpiScanpy using Dlouvain, Cleiden, and Clouvain on individual datasets. The scale of the dataset increases from left to right. **(B)** and **(C)** depict the peak memory usage (in GB) and total runtime (in minutes) of EpiScanpy and EpiCarousel respectively, both using the Dleiden, Dlouvain, Cleiden, and Clouvain clustering.

Fig. S2

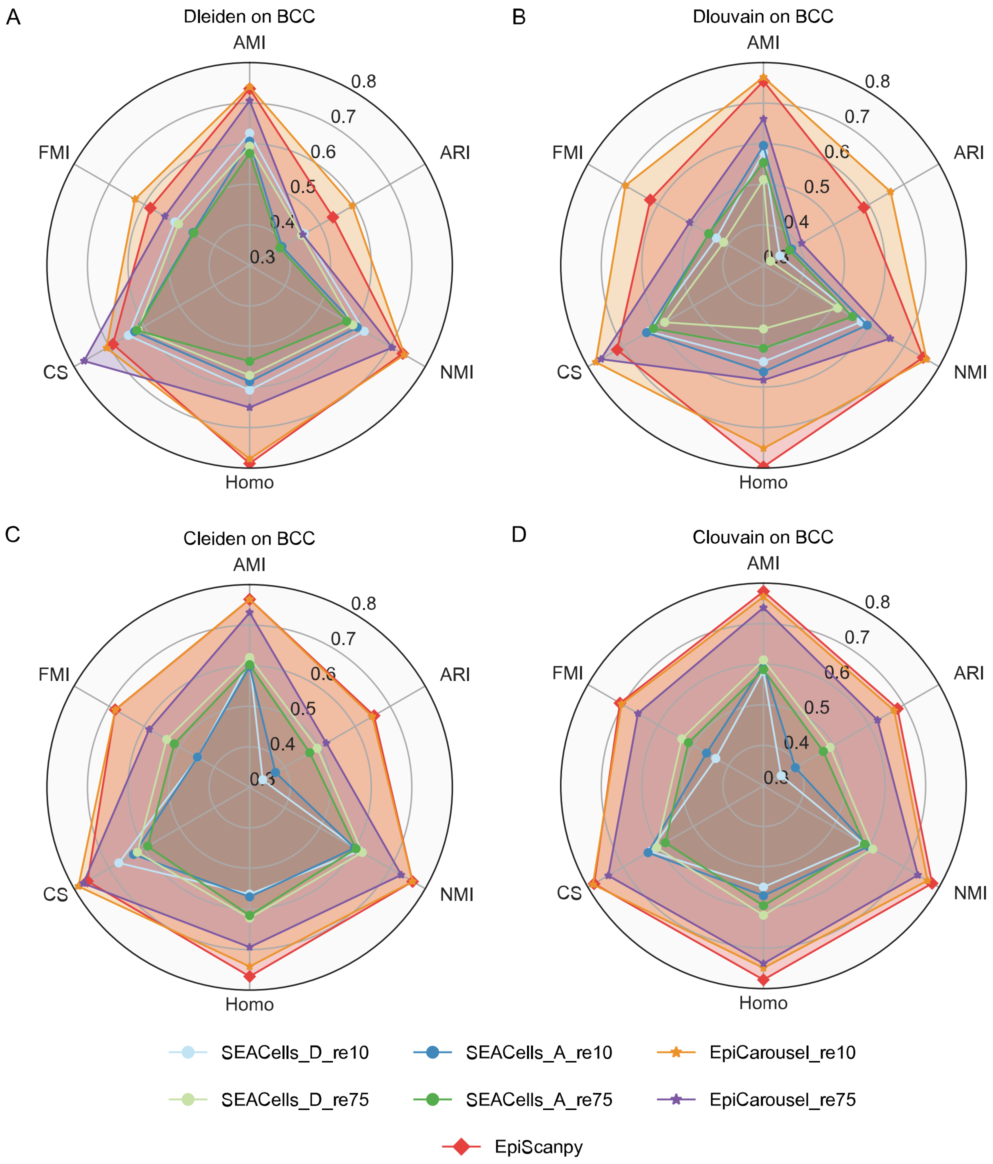

Fig. S2. Radar charts display the benchmarking results of EpiCarousel, SEACells, and EpiScanpy, using 4 clustering methods on the BCC dataset. **(A)**, **(B)**, **(C)**, and **(D)** depict the clustering results using Dleiden, Dlouvain, Cleiden, and Clouvain, respectively.

Fig. S3

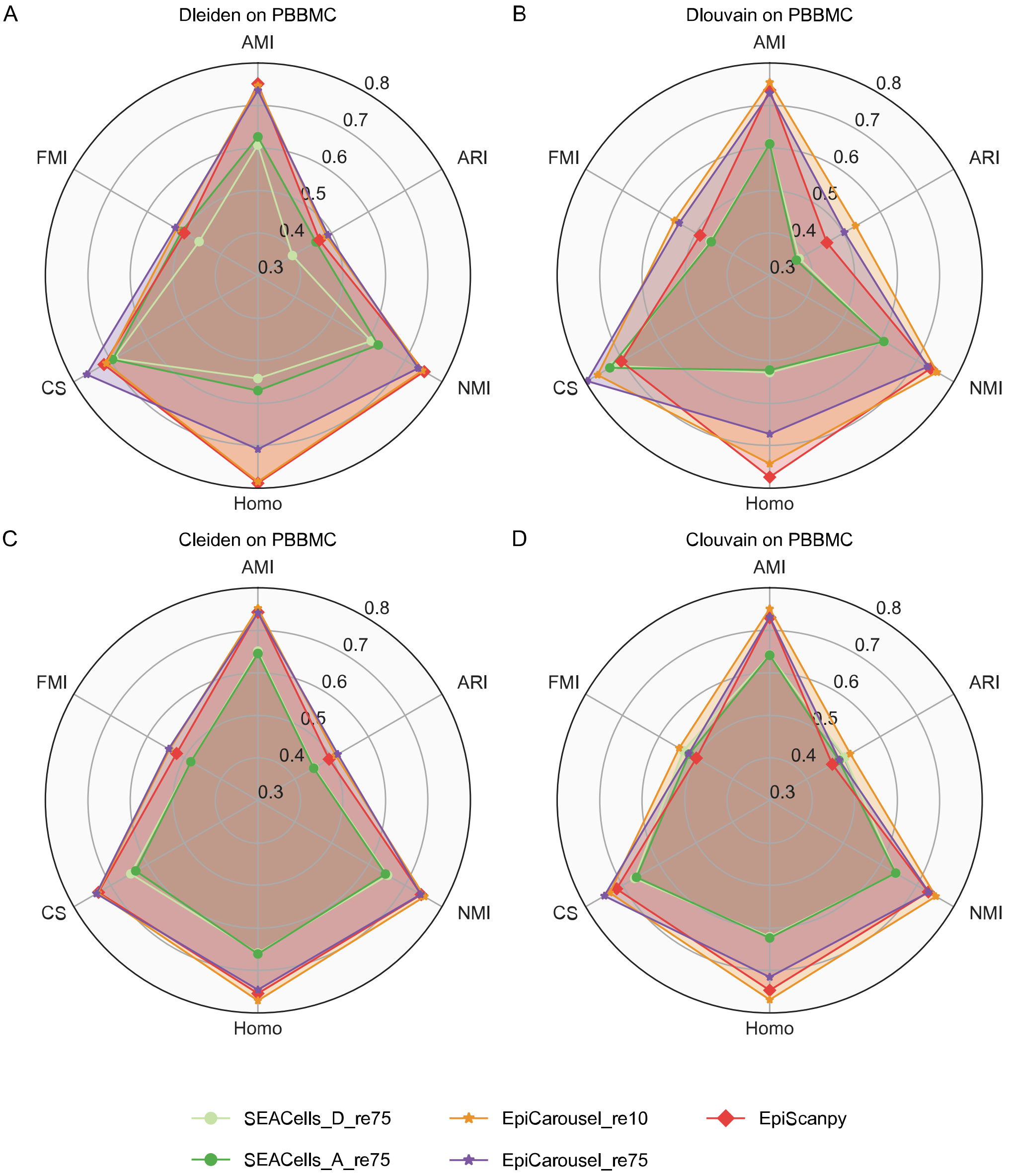

Fig. S3. Radar charts display the benchmarking results of EpiCarousel, SEACells, and EpiScanpy, using 4 clustering methods on the PBBMC dataset. **(A)**, **(B)**, **(C)**, and **(D)** depict the clustering results using Dleiden, Dlouvain, Cleiden, and Clouvain, respectively.

Fig. S4

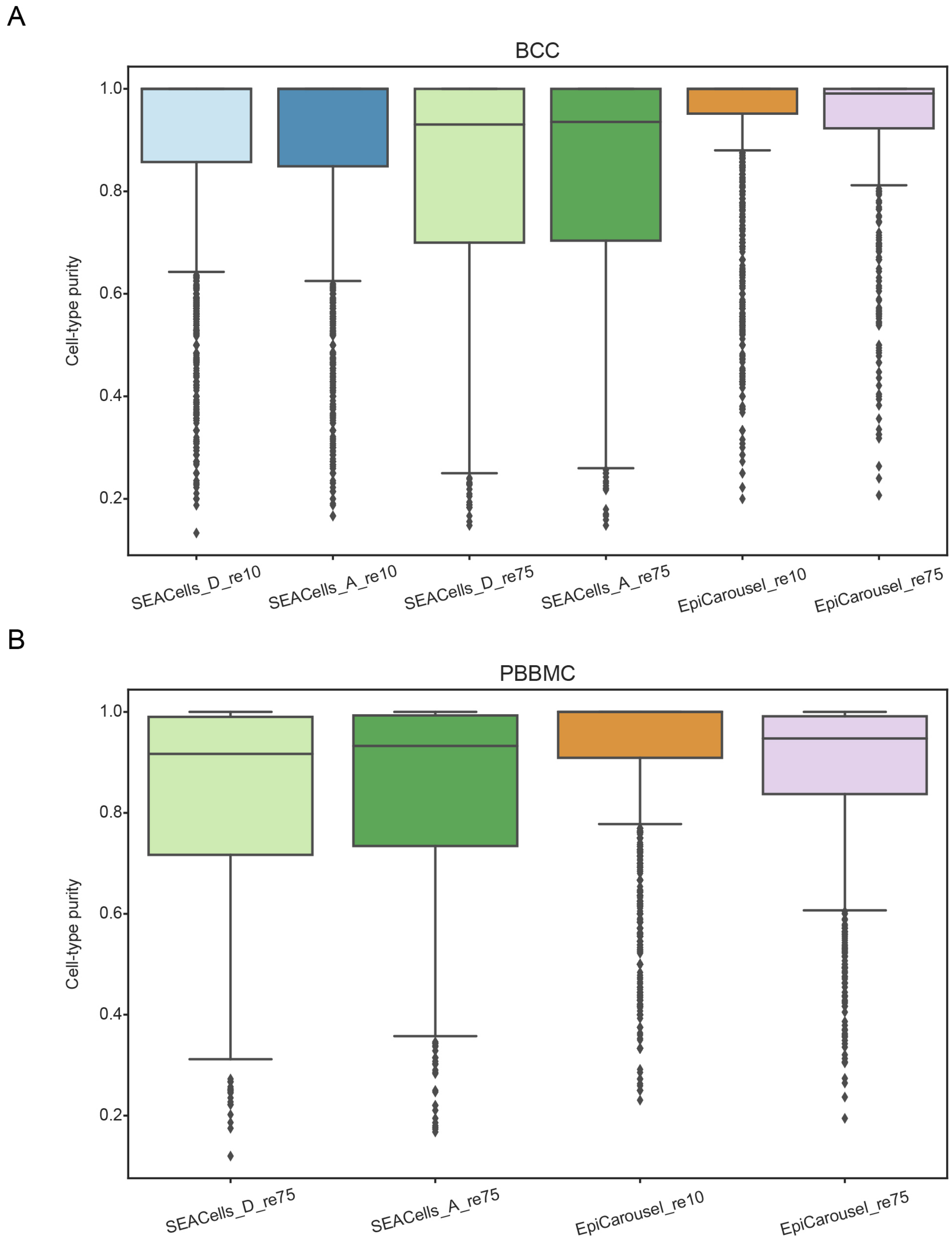

Fig. S4. Boxplots showing the purity of metacells identified by various methods on the BCC **(A)** and PBBMC **(B)** datasets.

Fig. S5

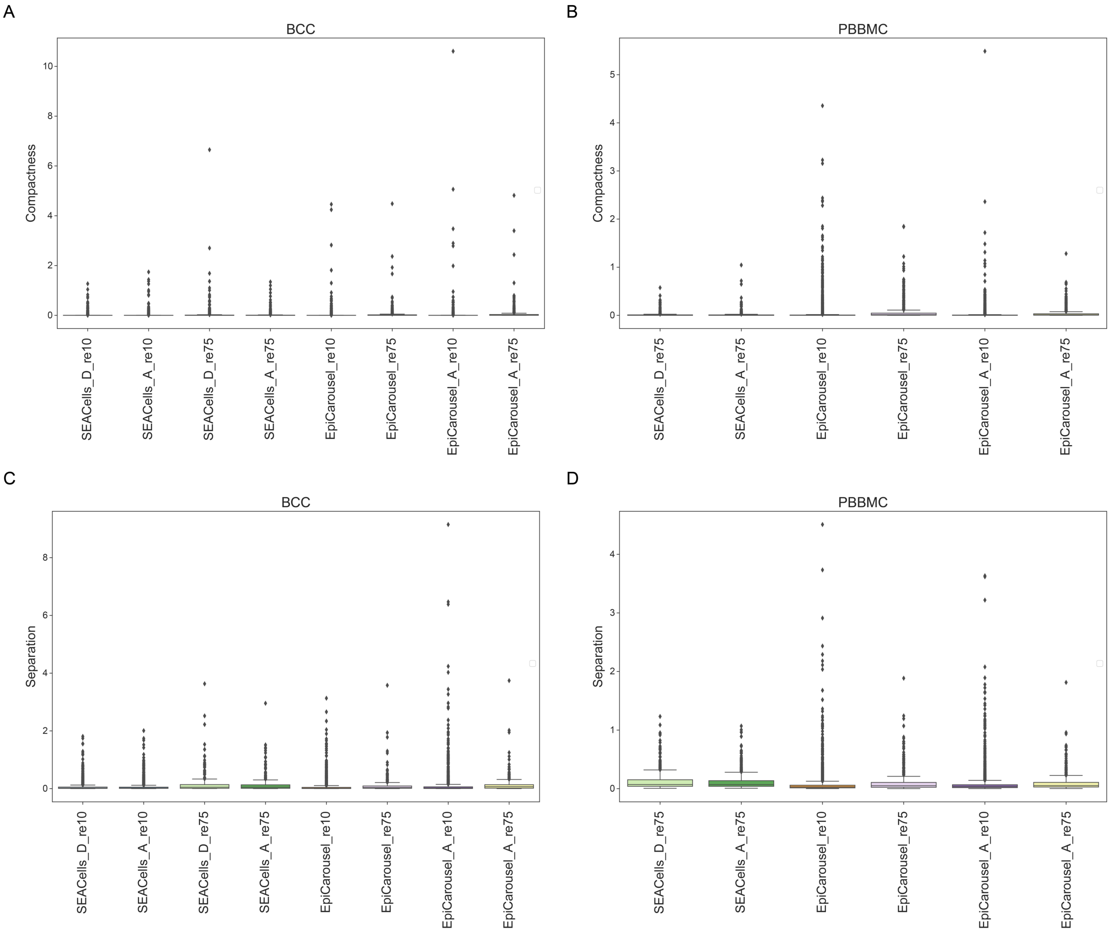

Fig. S5. Boxplots showing the compactness of metacells identified by various approaches on the BCC **(A)** and PBBMC **(B)** datasets, and the separation of metacells identified by various approaches on the BCC **(C)** and PBBMC **(D)** datasets.

Fig. S6

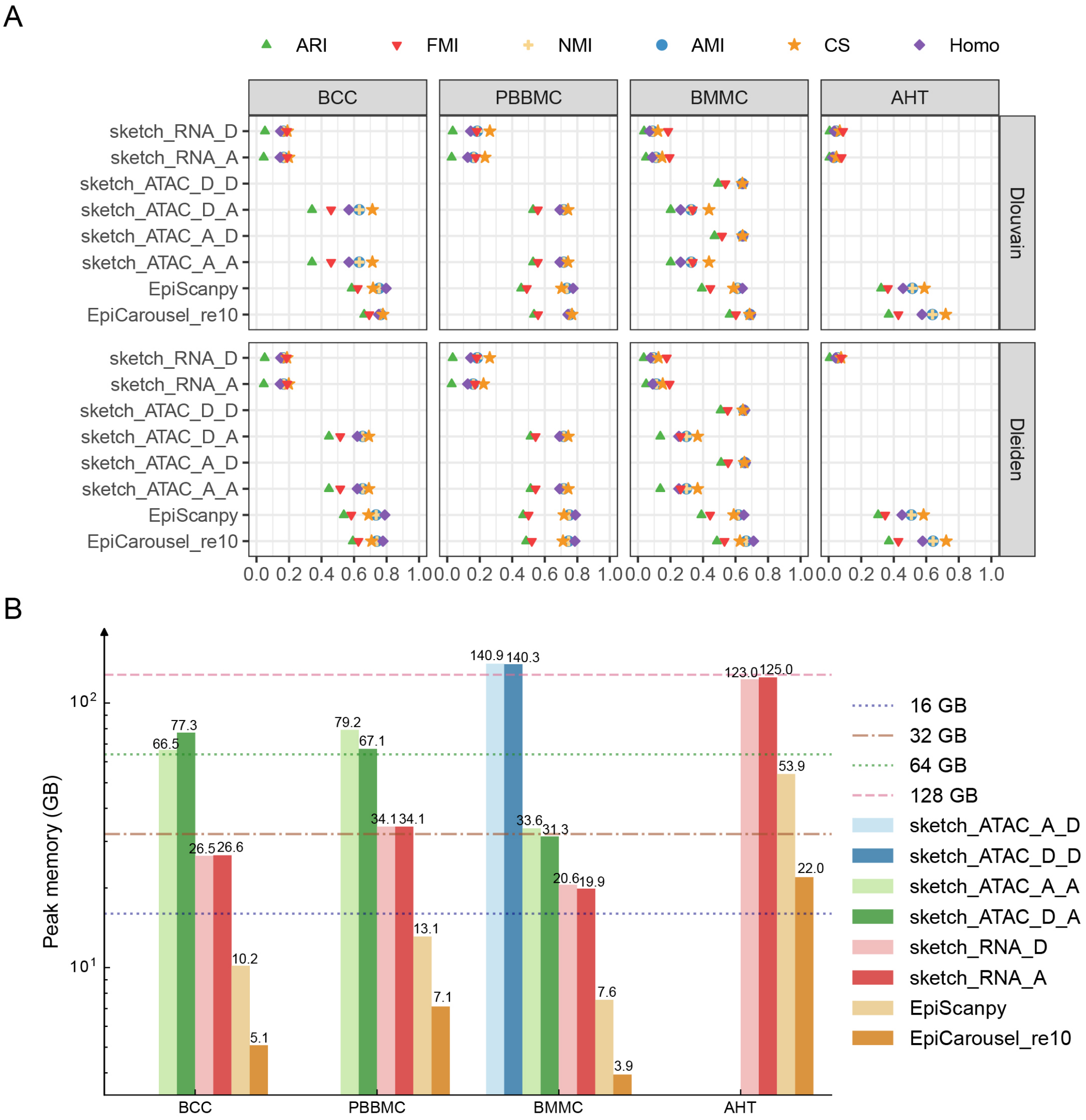

Fig. S6. Benchmarking results of various methods. **(A)** Clustering performance of different methods across various datasets using Dlouvain and Dleiden. **(B)** Plot of the peak memory usage of each method for benchmarking on various datasets

Fig. S7

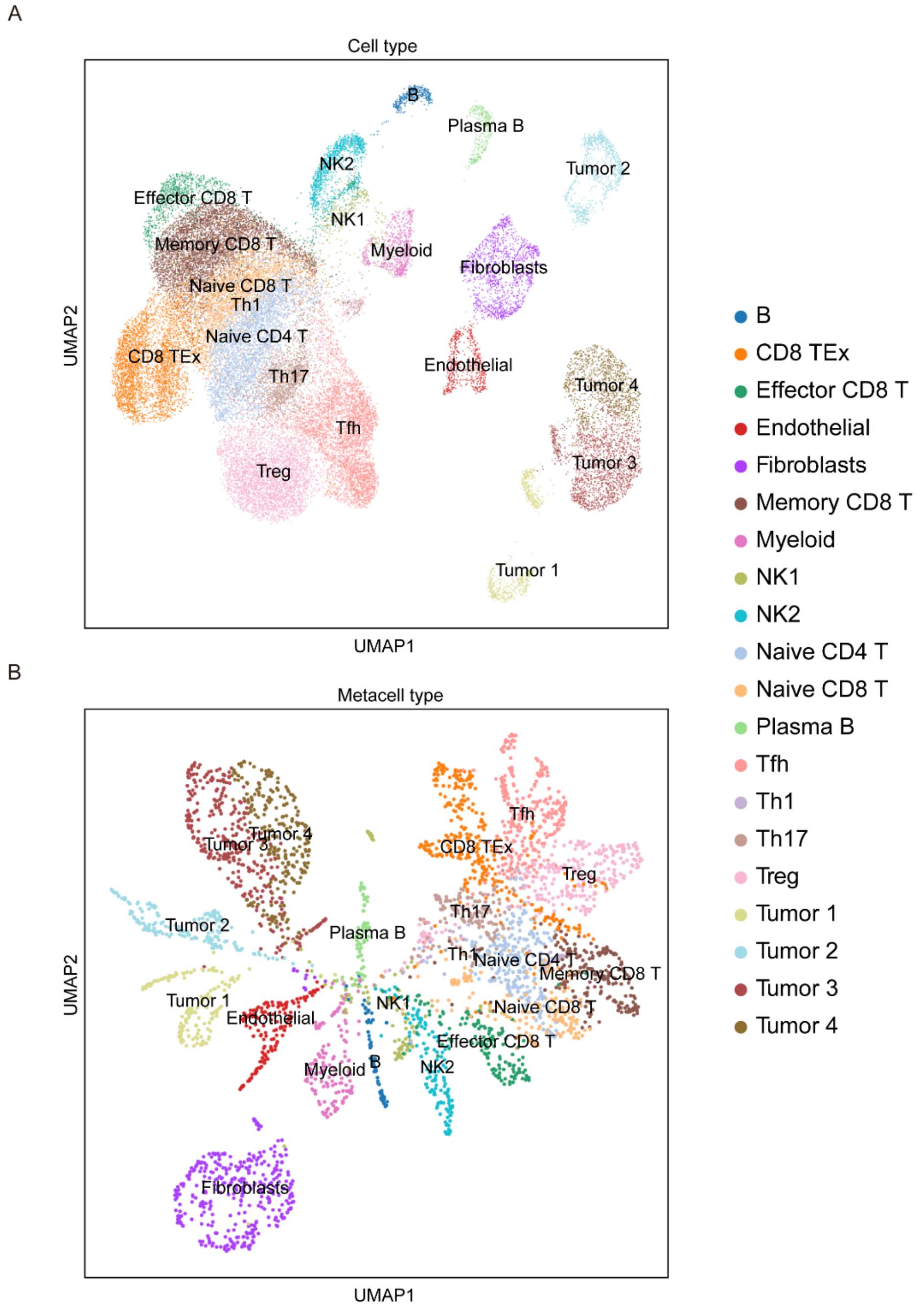

Fig. S7. UMAP visualization of BCC single cells and metacells. **(A)** UMAP visualization generated using the default workflow of EpiScanpy, where cells are colored by their true cell types of the BCC dataset. **(B)** UMAP visualization of the metacell matrix generated by EpiCarousel on BCC dataset. The cell type of each metacell is annotated as the most prevalent cell type among the comprising single cells. Metacells are colored by their cell types.

Fig. S8

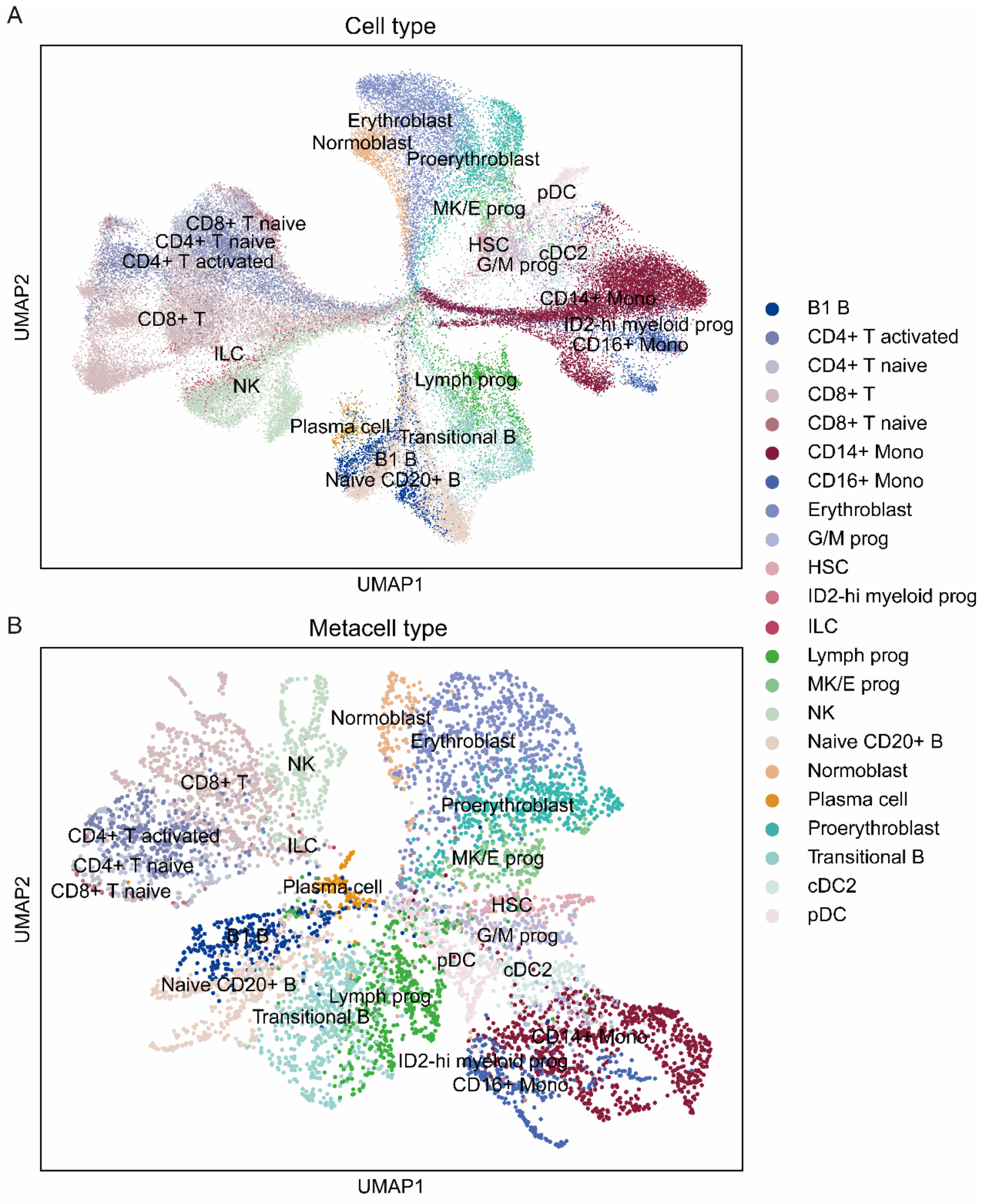

Fig. S8. UMAP visualization of BMMC single cells and metacells. **(A)** UMAP visualization generated using the default workflow of EpiScanpy, where cells are colored by their true cell types of the BMMC dataset. **(B)** UMAP visualization of the metacell matrix generated by EpiCarousel on BMMC dataset. The cell type of each metacell is annotated as the most prevalent cell type among the comprising single cells. Metacells are colored by their cell types.

Fig. S9

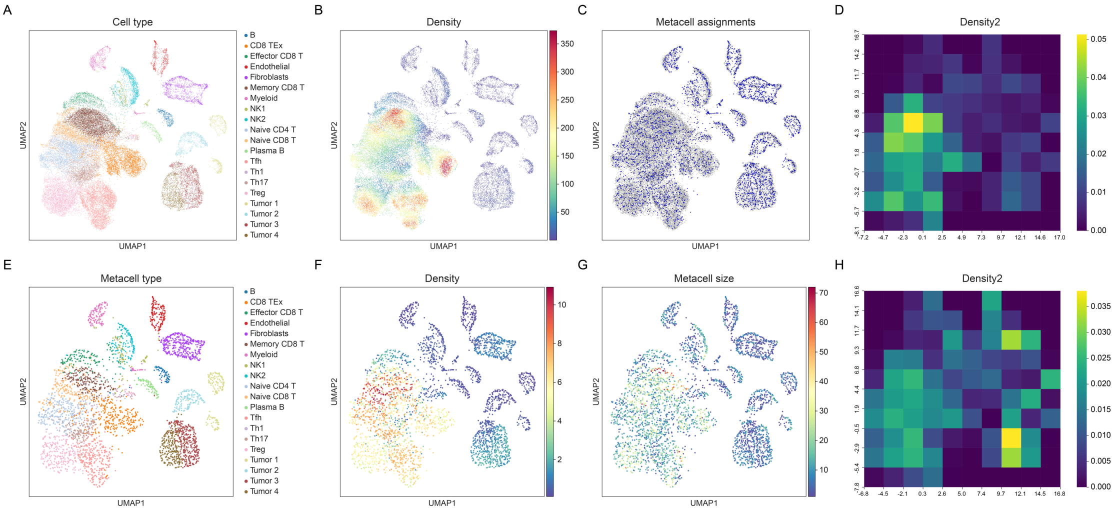

Fig. S9. The relationship between the distribution, size, and density of metacells identified by EpiCarosuel_re10 and the distribution and density of individual cells on the BCC dataset. UMAPs of single-cell data preprocessed through EpiCarousel, colored by cell types **(A)** and density **(B)** of single cells, respectively. **(C)** UMAP of single-cell data preprocessed through EpiCarousel, where gray dots represent single cells, and blue dots represent metacells identified by EpiCarousel_re10. **(D)** Heatmap of Density2 on 100 equal parts of the UMAP of single-cell data preprocessed by EpiCarousel. UMAPs of metacells, where the UMAP coordinates of metacells are obtained by averaging the UMAPs of the constituent single cells, colored by metacell types **(E)**, metacell density **(F)**, and metacell size **(G),** respectively. **(H)** Heatmap of Density2 on 100 equal parts of the above UMAP of metacells.

Fig. S10

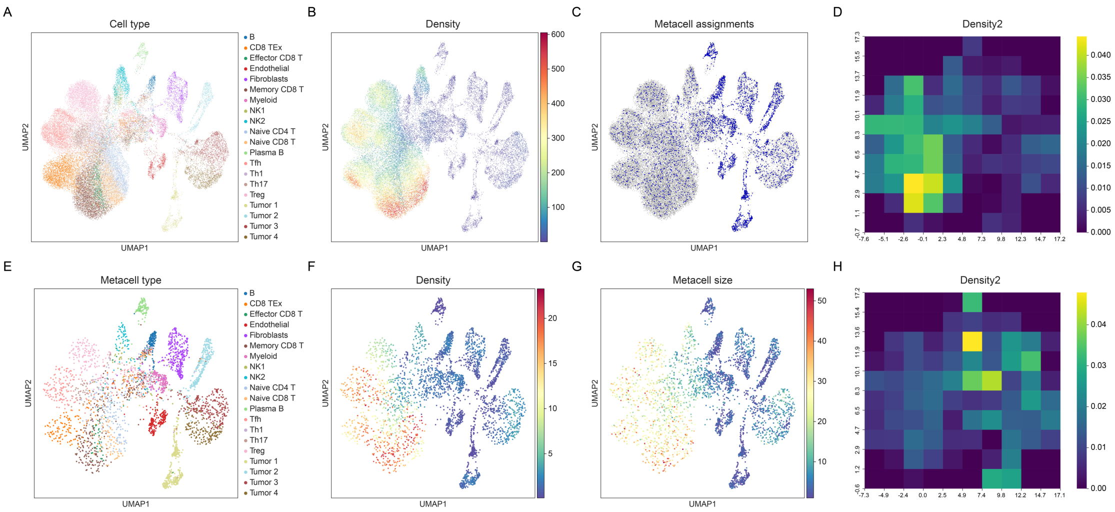

Fig. S10. The relationship between the distribution, size, and density of metacells identified by SEACells_D_re10 and the distribution and density of individual cells on the BCC dataset. UMAPs of single-cell data preprocessed following SEACells, colored by cell types **(A)** and density **(B)** of single cells, respectively. **(C)** UMAP of single-cell data preprocessed following SEACells, where gray dots represent single cells, and blue dots represent metacells identified by SEACells_D_re10. **(D)** Heatmap of Density2 on 100 equal parts of the UMAP of single-cell data preprocessed following SEACells. UMAPs of metacells, where the UMAP coordinates of metacells are obtained by averaging the UMAPs of the constituent single cells, colored by metacell types **(E)**, metacell density **(F)**, and metacell size **(G)**, respectively. **(H)** Heatmap of Density2 on 100 equal parts of the above UMAP of metacells.

Fig. S11

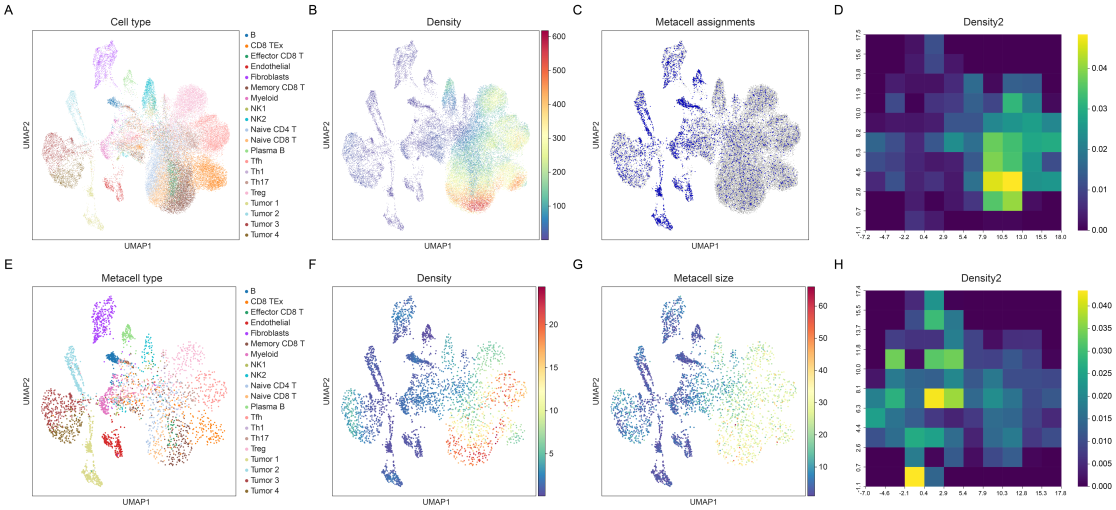

Fig. S11. The relationship between the distribution, size, and density of metacells identified by SEACells_A_re10 and the distribution and density of individual cells on the BCC dataset. UMAPs of single-cell data preprocessed following SEACells, colored by cell types **(A)** and density **(B)** of single cells, respectively. **(C)** UMAP of single-cell data preprocessed following SEACells, where gray dots represent single cells, and blue dots represent metacells identified by SEACells_A_re10. **(D)** Heatmap of Density2 on 100 equal parts of the UMAP of single-cell data preprocessed following SEACells. UMAPs of metacells, where the UMAP coordinates of metacells are obtained by averaging the UMAPs of the constituent single cells, colored by metacell types **(E)**, metacell density **(F)**, and metacell size **(G)**, respectively. **(H)** Heatmap of Density2 on 100 equal parts of the above UMAP of metacells.

Fig. S12

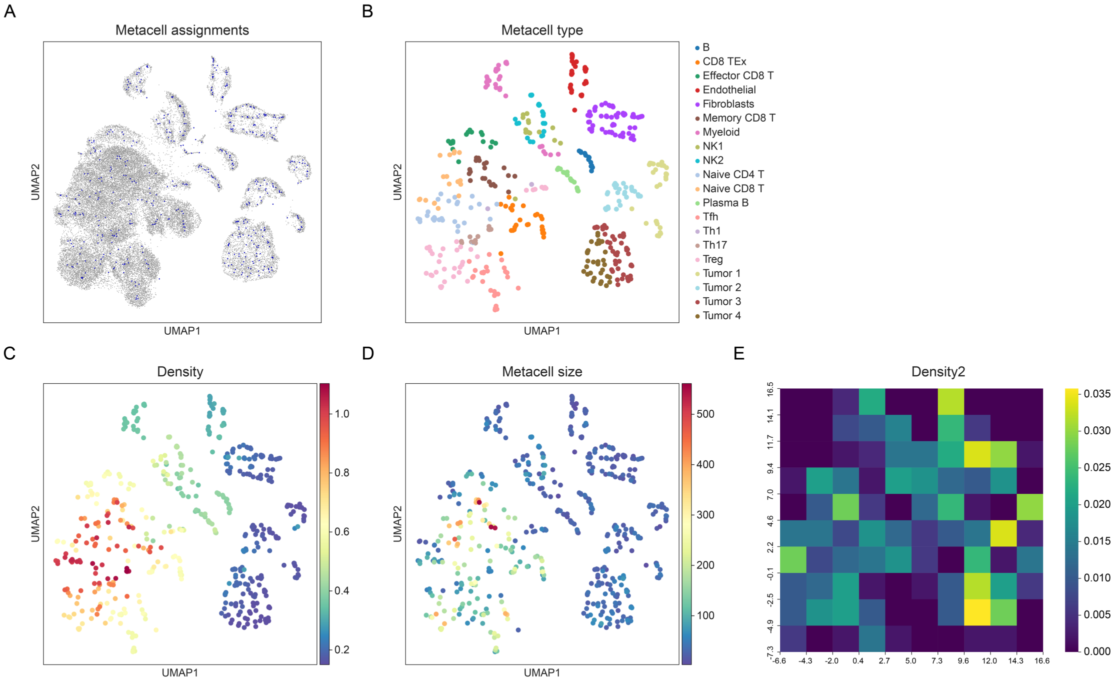

Fig. S12. The relationship between the distribution, size, and density of metacells identified by EpiCarosuel_re75 and the distribution and density of individual cells on the BCC dataset. **(A)** UMAP of single-cell data preprocessed through EpiCarousel, where gray dots represent single cells, and blue dots represent metacells identified by EpiCarousel_re75. UMAPs of metacells, where the UMAP coordinates of metacells are obtained by averaging the UMAPs of the constituent single cells, colored by metacell types **(B)**, metacell density **(C)**, and metacell size **(D)**, respectively. **(E)** Heatmap of Density2 on 100 equal parts of the above UMAP of metacells.

Fig. S13

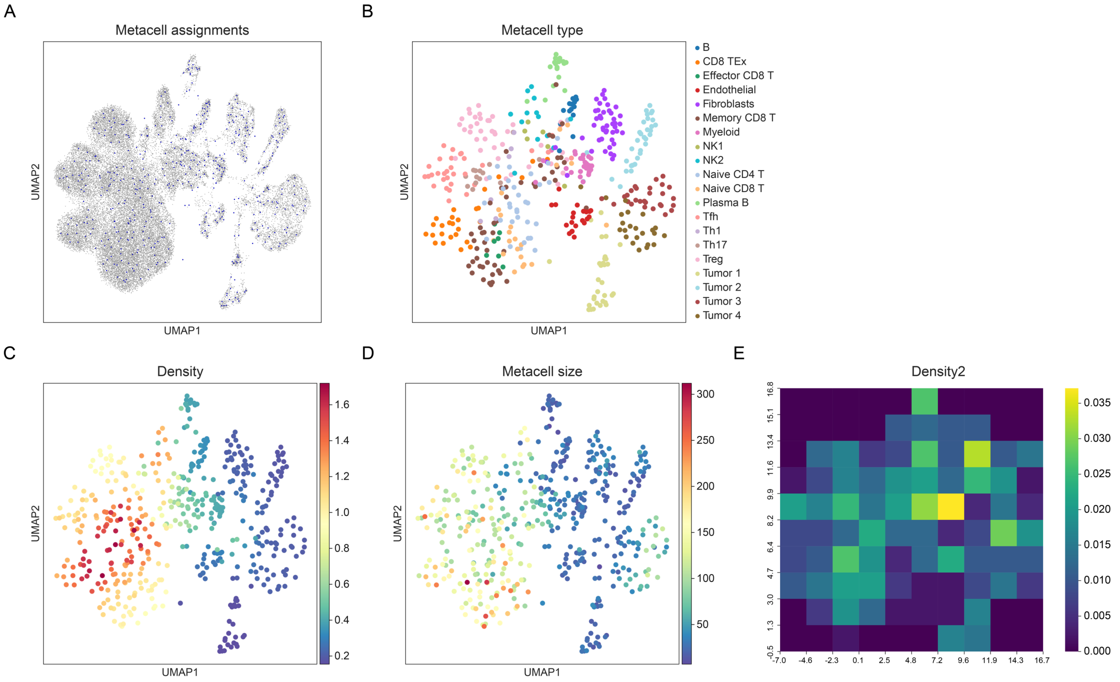

Fig. S13. The relationship between the distribution, size, and density of metacells identified by SEACells_D_re75 and the distribution and density of individual cells on the BCC dataset. **(A)** UMAP of single-cell data preprocessed following SEACells, where gray dots represent single cells, and blue dots represent metacells identified by SEACells_D_re75. UMAPs of metacells, where the UMAP coordinates of metacells are obtained by averaging the UMAPs of the constituent single cells, colored by metacell types **(B)**, metacell density **(C)**, and metacell size **(D)**, respectively. **(E)** Heatmap of Density2 on 100 equal parts of the above UMAP of metacells.

Fig. S14

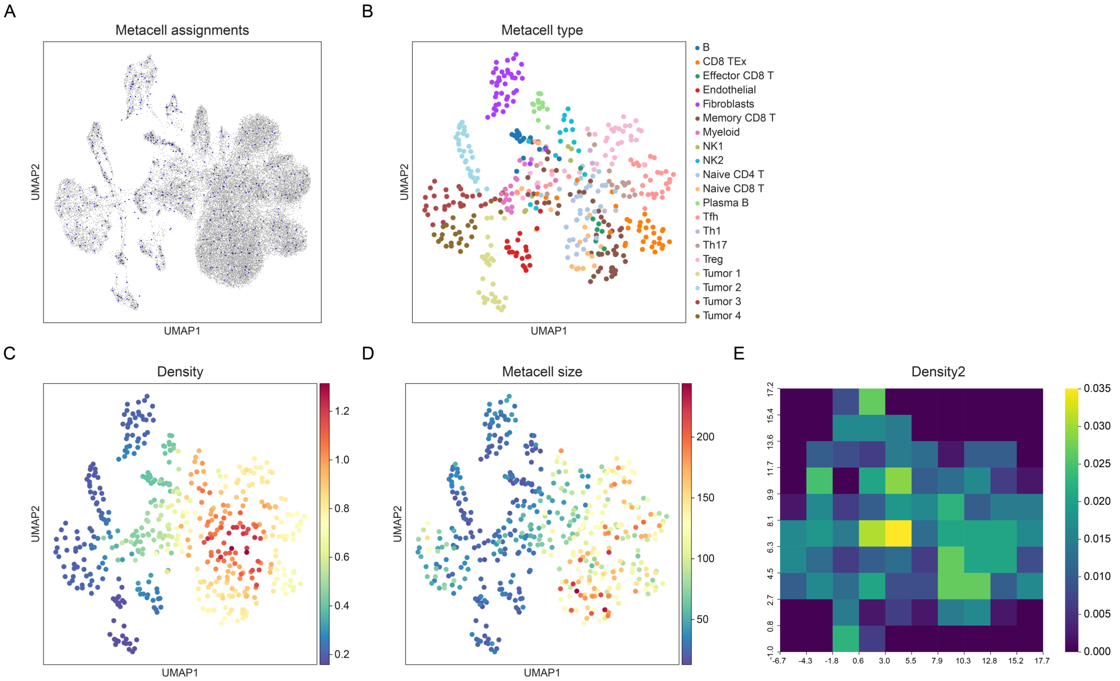

Fig. S14. The relationship between the distribution, size, and density of metacells identified by SEACells_A_re75 and the distribution and density of individual cells on the BCC dataset. **(A)** UMAP of single-cell data preprocessed following SEACells, where gray dots represent single cells, and blue dots represent metacells identified by SEACells_A_re75. UMAPs of metacells, where the UMAP coordinates of metacells are obtained by averaging the UMAPs of the constituent single cells, colored by metacell types **(B)**, metacell density **(C)**, and metacell size **(D)**, respectively. **(E)** Heatmap of Density2 on 100 equal parts of the above UMAP of metacells.

Fig. S15

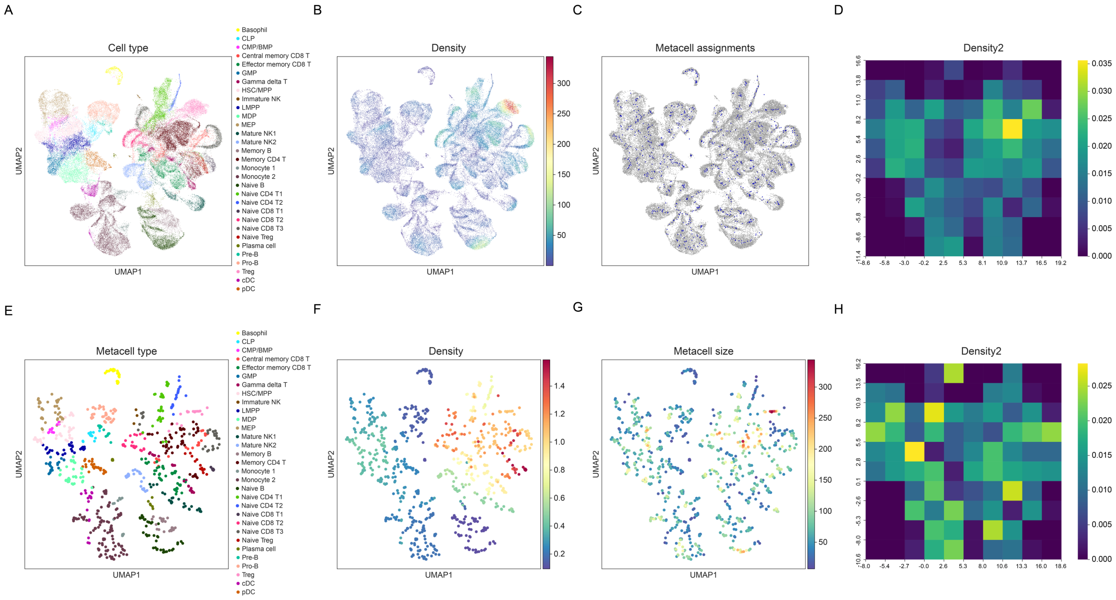

Fig. S15. The relationship between the distribution, size, and density of metacells identified by EpiCarosuel_re75 and the distribution and density of individual cells on the PBBMC dataset. UMAPs of single-cell data preprocessed through EpiCarousel, colored by cell types **(A)** and density **(B)** of single cells, respectively. **(C)** UMAP of single-cell data preprocessed through EpiCarousel, where gray dots represent single cells, and blue dots represent metacells identified by EpiCarousel_re75. **(D)** Heatmap of Density2 on 100 equal parts of the UMAP of single-cell data preprocessed by EpiCarousel. UMAPs of metacells, where the UMAP coordinates of metacells are obtained by averaging the UMAPs of the constituent single cells, colored by metacell types **(E)**, metacell density **(F)**, and metacell size **(G)**, respectively. **(H)** Heatmap of Density2 on 100 equal parts of the above UMAP of metacells.

Fig. S16

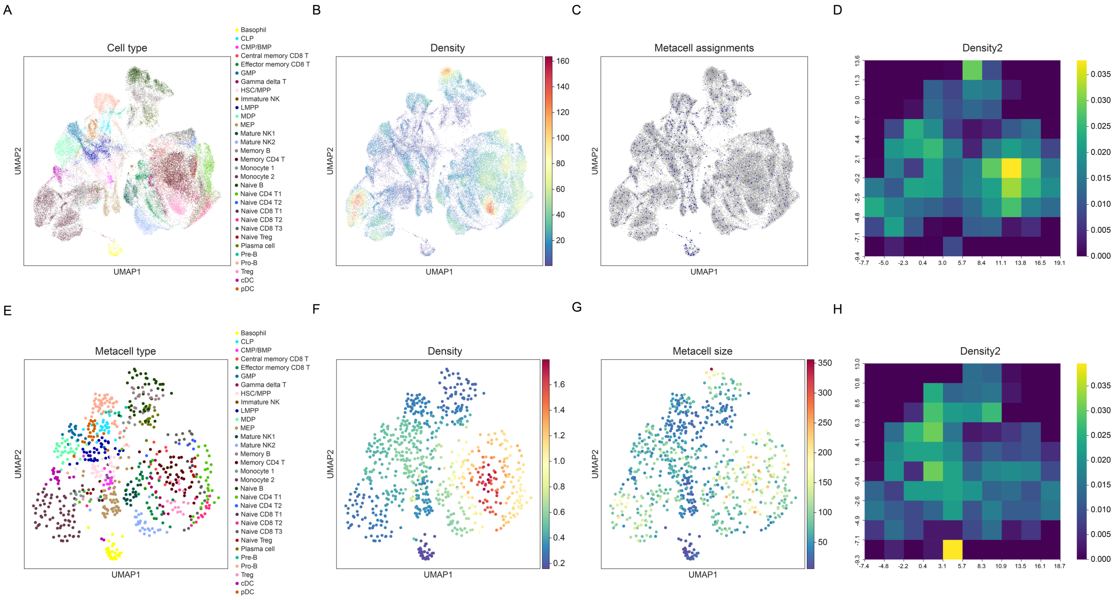

Fig. S16. The relationship between the distribution, size, and density of metacells identified by SEACells_D_re75 and the distribution and density of individual cells on the PBBMC dataset. UMAPs of single-cell data preprocessed following SEACells, colored by cell types **(A)** and density **(B)** of single cells, respectively. **(C)** UMAP of single-cell data preprocessed following SEACells, where gray dots represent single cells, and blue dots represent metacells identified by SEACells_D_re75. **(D)** Heatmap of Density2 on 100 equal parts of the UMAP of single-cell data preprocessed following SEACells. UMAPs of metacells, where the UMAP coordinates of metacells are obtained by averaging the UMAPs of the constituent single cells, colored by metacell types **(E)**, metacell density **(F)**, and metacell size **(G)**, respectively. **(H)** Heatmap of Density2 on 100 equal parts of the above UMAP of metacells.

Fig. S17

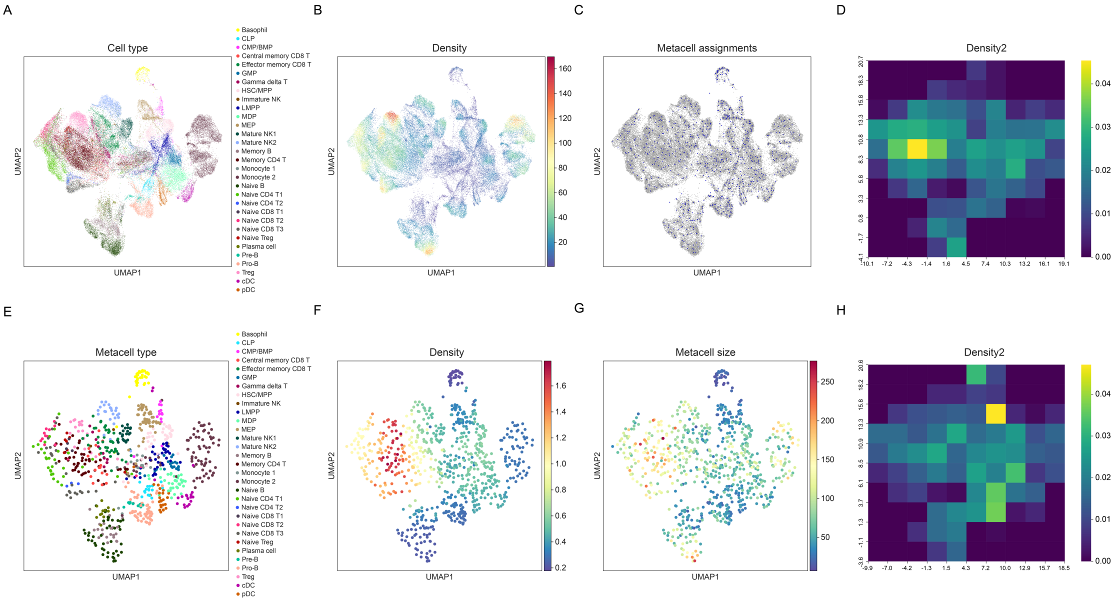

Fig. S17. The relationship between the distribution, size, and density of metacells identified by SEACells_A_re75 and the distribution and density of individual cells on the PBBMC dataset. UMAPs of single-cell data preprocessed following SEACells, colored by cell types **(A)** and density **(B)** of single cells, respectively. **(C)** UMAP of single-cell data preprocessed following SEACells, where gray dots represent single cells, and blue dots represent metacells identified by SEACells_A_re75. **(D)** Heatmap of Density2 on 100 equal parts of the UMAP of single-cell data preprocessed following SEACells. UMAPs of metacells, where the UMAP coordinates of metacells are obtained by averaging the UMAPs of the constituent single cells, colored by metacell types **(E)**, metacell density **(F)**, and metacell size **(G)**, respectively. **(H)** Heatmap of Density2 on 100 equal parts of the above UMAP of metacells.

Fig. S18

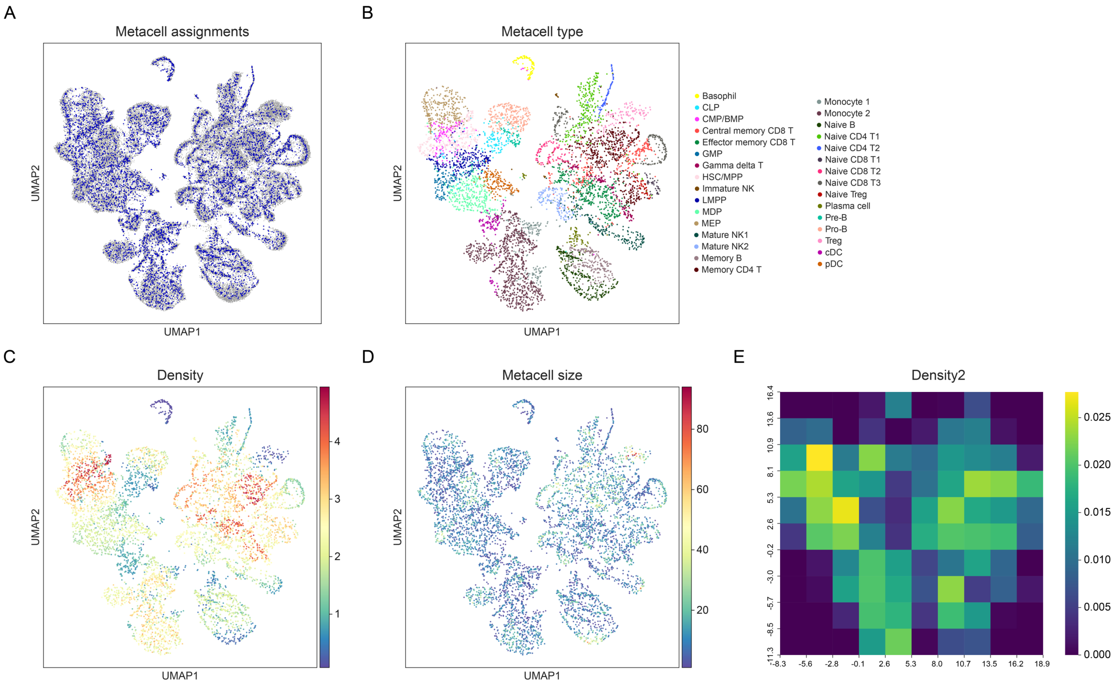

Fig. S18. The relationship between the distribution, size, and density of metacells identified by EpiCarosuel_re10 and the distribution and density of individual cells on the PBBMC dataset. **(A)** UMAP of single-cell data preprocessed through EpiCarousel, where gray dots represent single cells, and blue dots represent metacells identified by EpiCarousel_re10. UMAPs of metacells, where the UMAP coordinates of metacells are obtained by averaging the UMAPs of the constituent single cells, colored by metacell types **(B)**, metacell density **(C)**, and metacell size **(D)**, respectively. **(E)** Heatmap of Density2 on 100 equal parts of the above UMAP of metacells.

Fig. S19

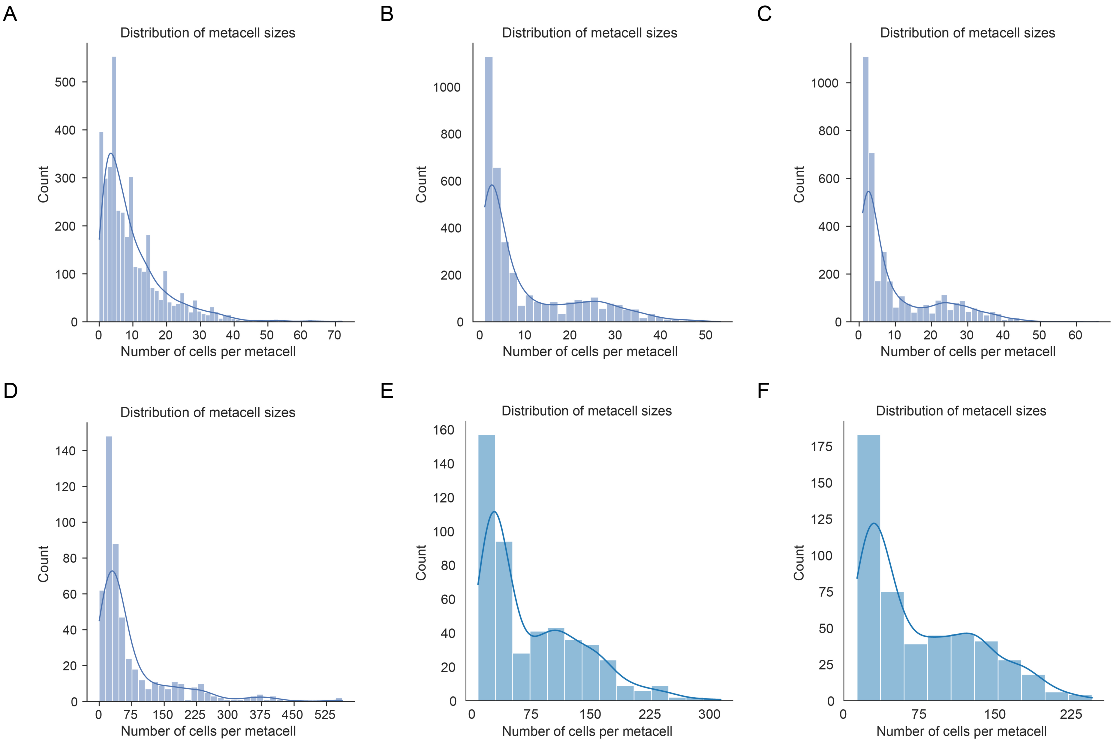

Fig. S19. The distributions of metacell sizes obtained by EpiCarousel_re10 **(A)**, SEACells_D_re10 **(B)**, SEACells_A_re10 **(C)**, EpiCarousel_re75 **(D)**, SEACells_D_re75 **(E)**, and SEACells_A_re75 **(F)** on the BCC dataset, respectively. Curves indicate corresponding kernel density estimates (KDEs).

Fig. S20

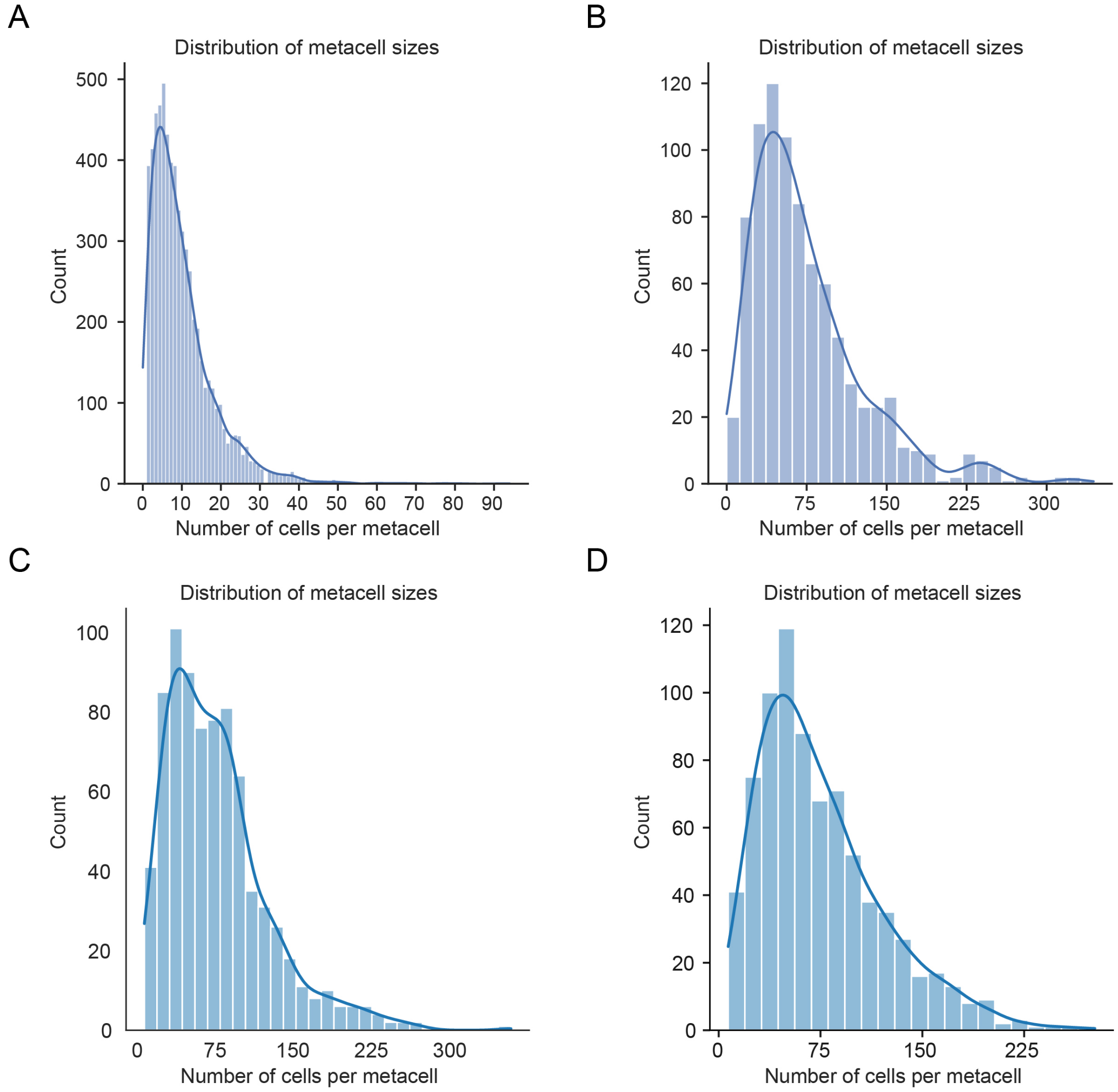

Fig. S20. The distributions of metacell sizes obtained by EpiCarousel_re10 **(A)**, EpiCarousel_re75 **(B)**, SEACells_D_re75 **(C)**, and SEACells_A_re75 **(D)** on the PBBMC dataset, respectively. Curves indicate corresponding kernel density estimates (KDEs).

Fig. S21

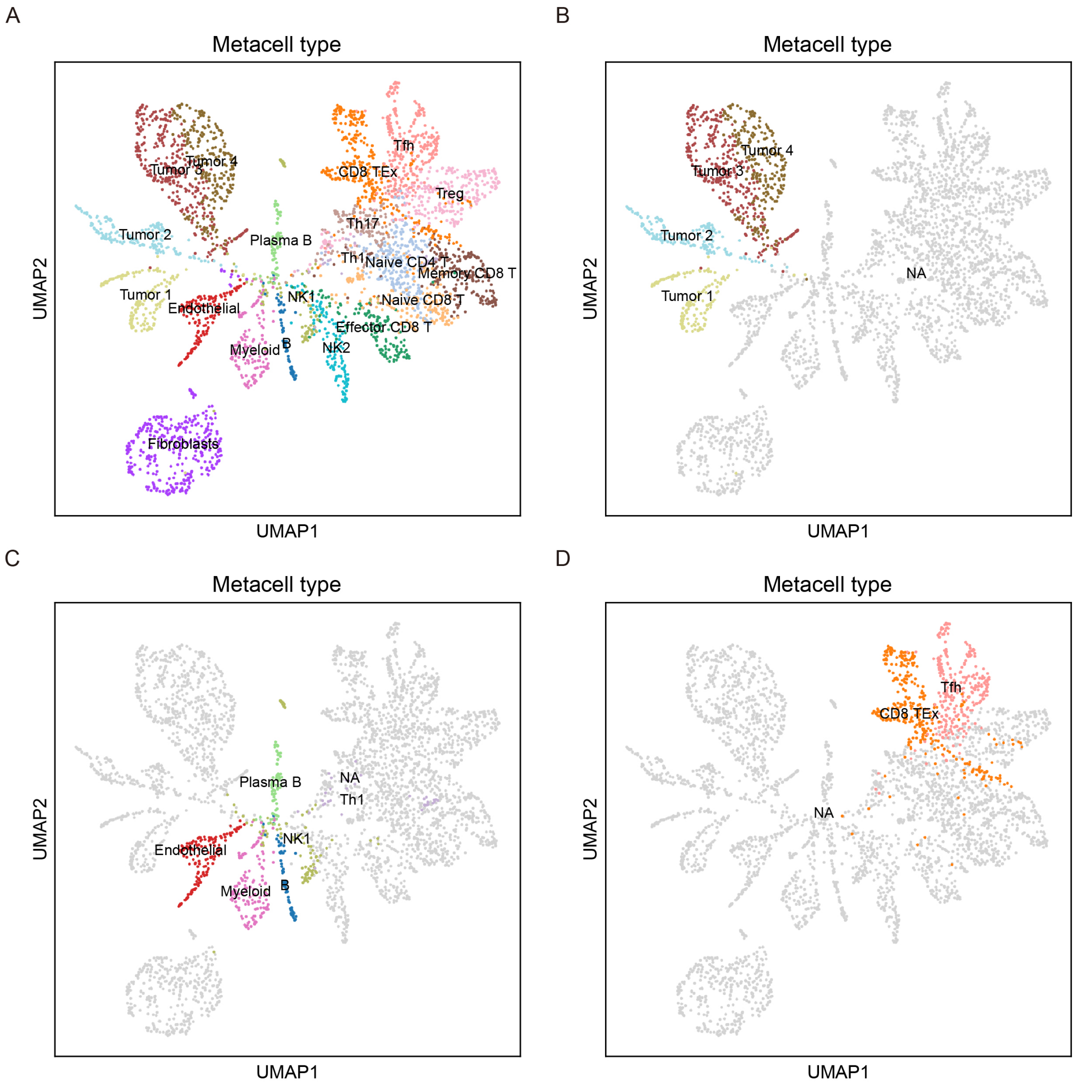

Fig. S21. UMAPs showing metacells identified by EpiCarousel on BCC profiles. All metacell types are colored in **(A)**.Four types of tumor cells are colored in **(B)**. Six rare cell types are colored in **(C)**. Two subtypes of therapy-responsive T cells are colored in **(D)**. NA represents other uncolored cell types in **(B)**, **(C)** and **(D)**.

Fig. S22

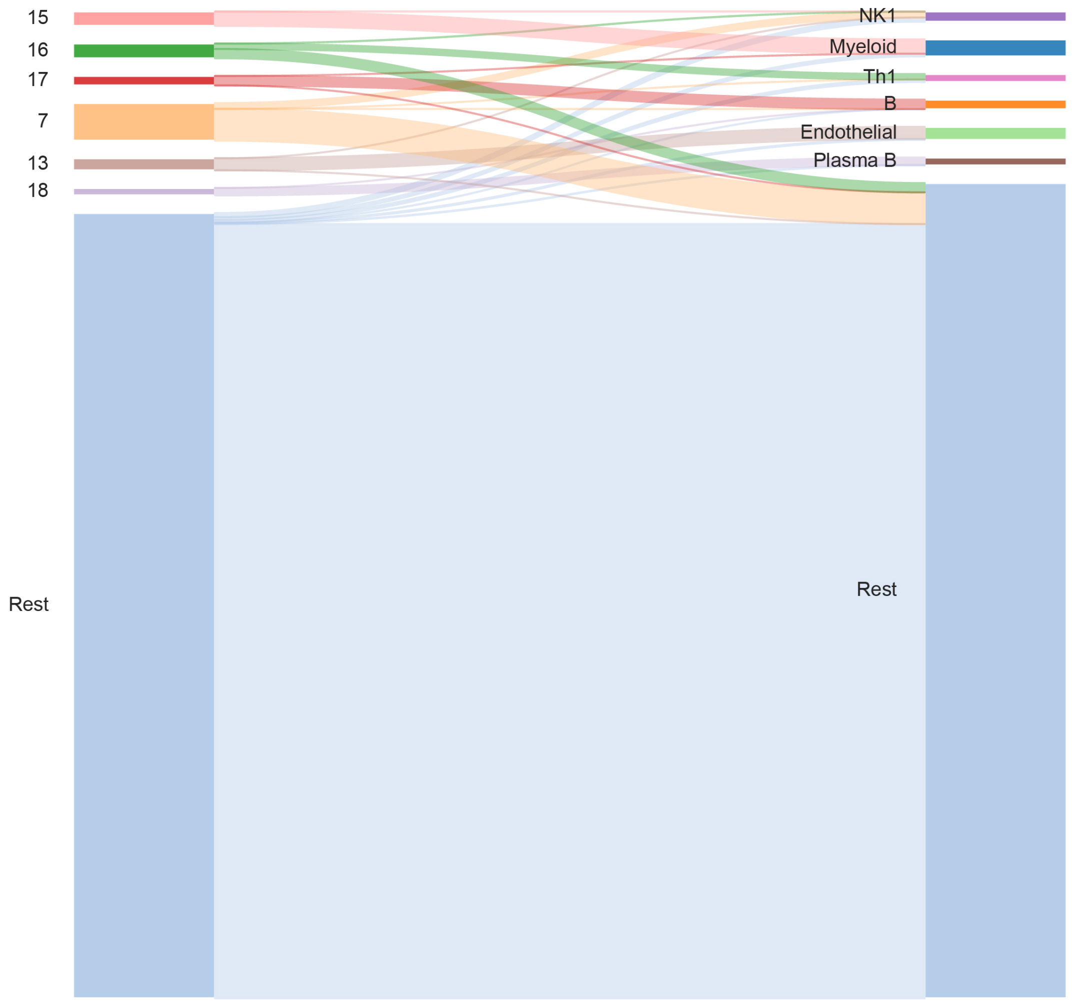

Fig. S22. Sankey diagram illustrating the 7 vs. 7 correspondence between mc_Dlouvain labels and true rare cell type labels of single cells on the BCC dataset.

Fig. S23

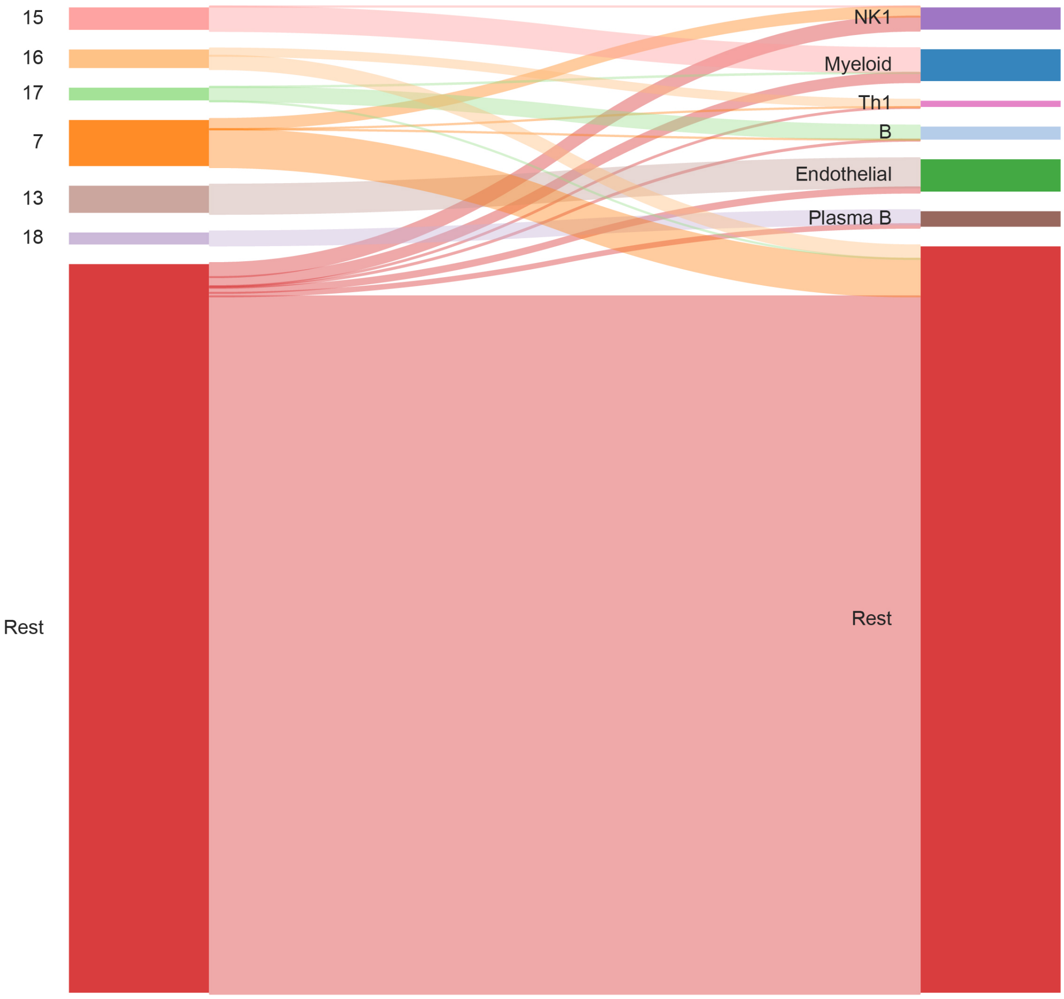

Fig. S23. Sankey diagram illustrating the 7 vs. 7 correspondence between Dlouvain labels and rare metacell type labels of metacells identified by EpiCarousel on the BCC dataset.

Fig. S24

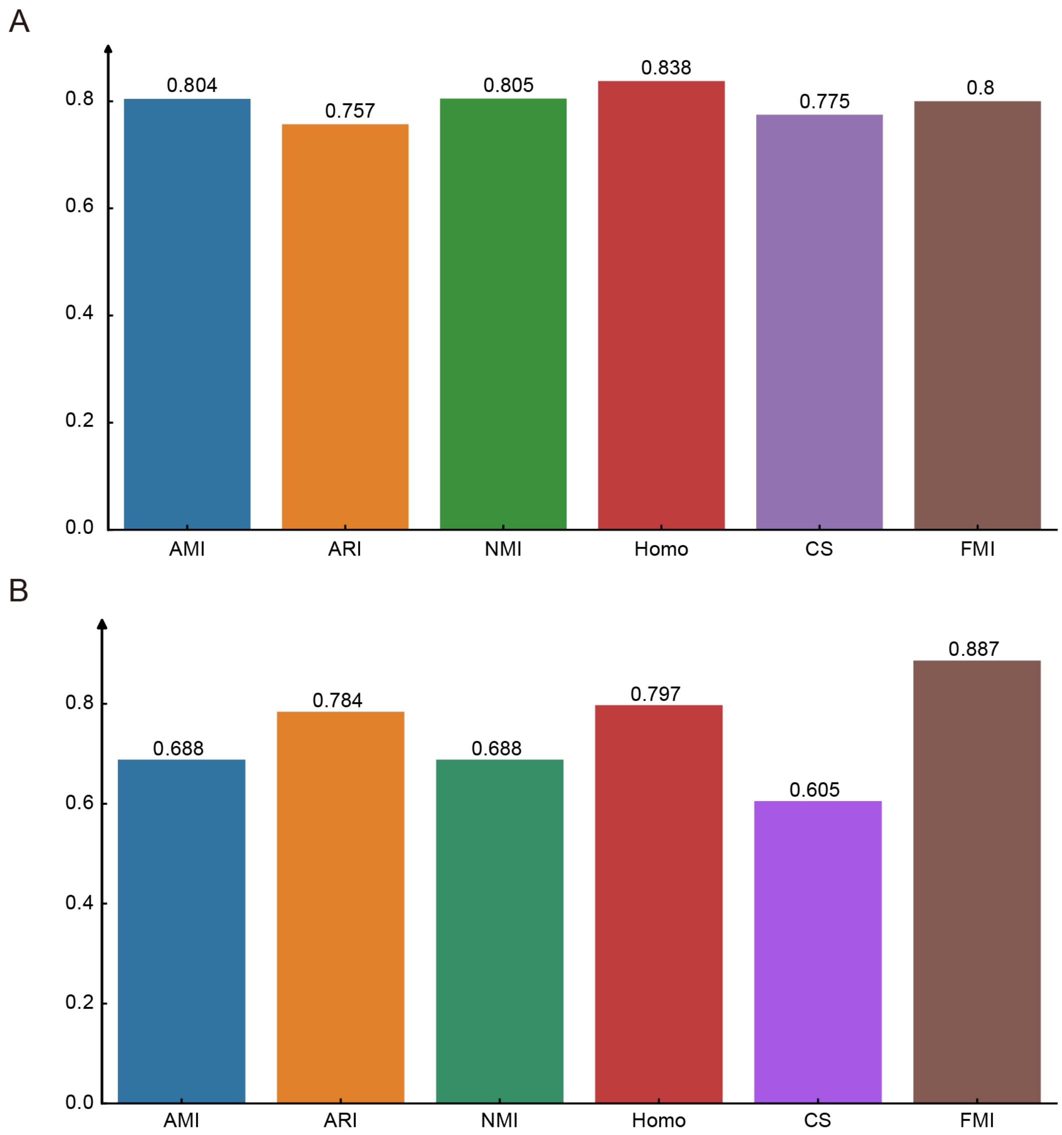

Fig. S24. Dlouvain clustering performance of EpiCarousel on the subset of six rare cell types Plasma B, Th1, B, NK1, Endothelial, and Myeloid **(A)** and the subset of two subtypes CD8 TEx and Tfh **(B)** of the full BCC clustering results.

Fig. S25

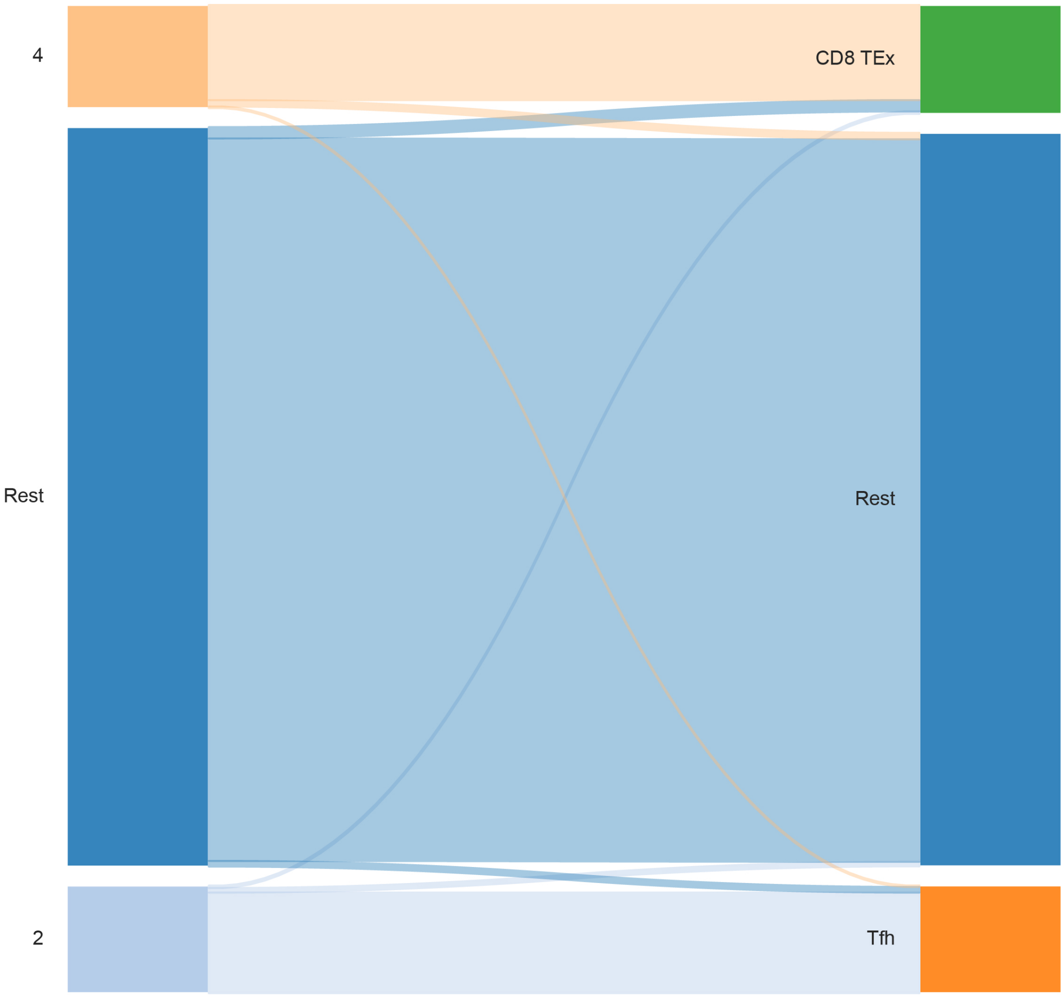

Fig. S25. Sankey diagram illustrating the 3 vs. 3 correspondence between mc_Dlouvain labels and true sub-cell type labels of single cells on the BCC dataset.

Fig. S26

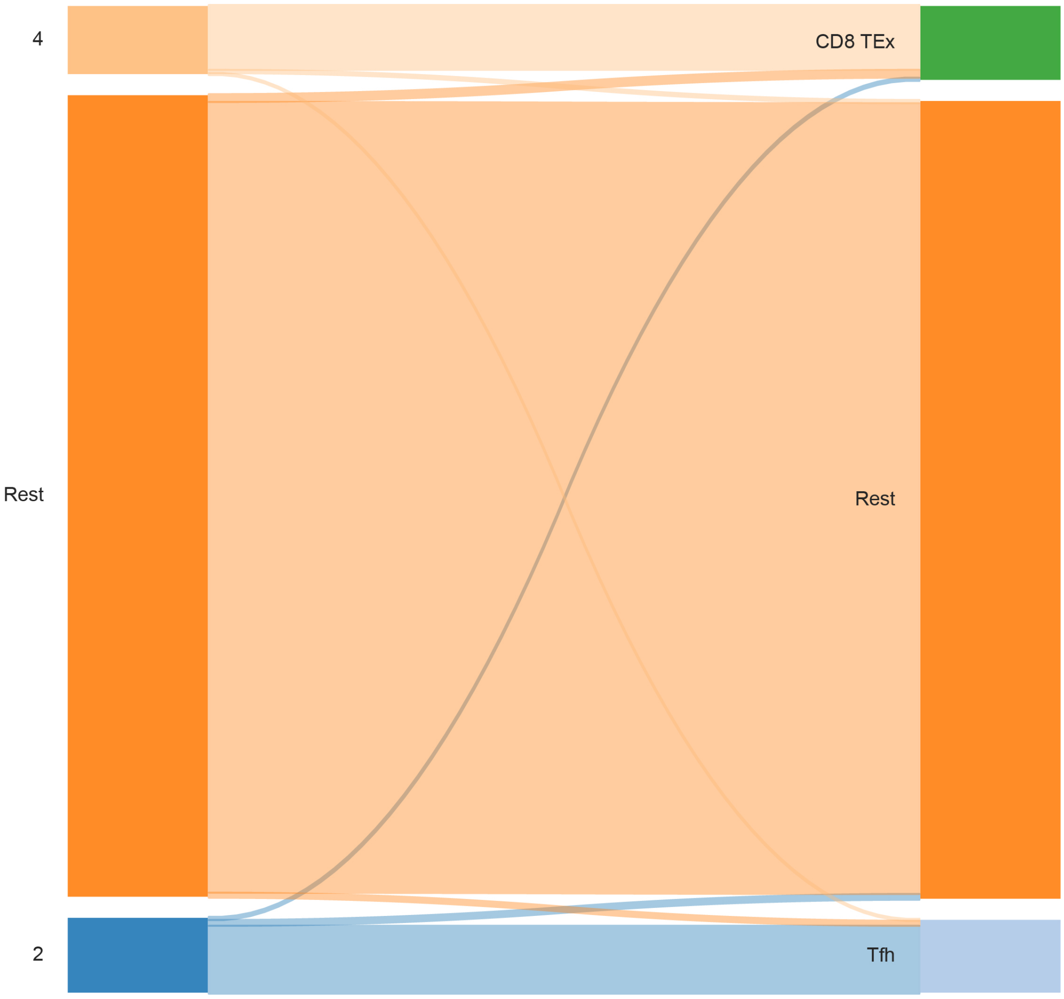

Fig. S26. Sankey diagram illustrating the 3 vs. 3 correspondence between Dlouvain labels and sub metacell type labels of metacells identified by EpiCarousel on the BCC dataset.

Fig. S27

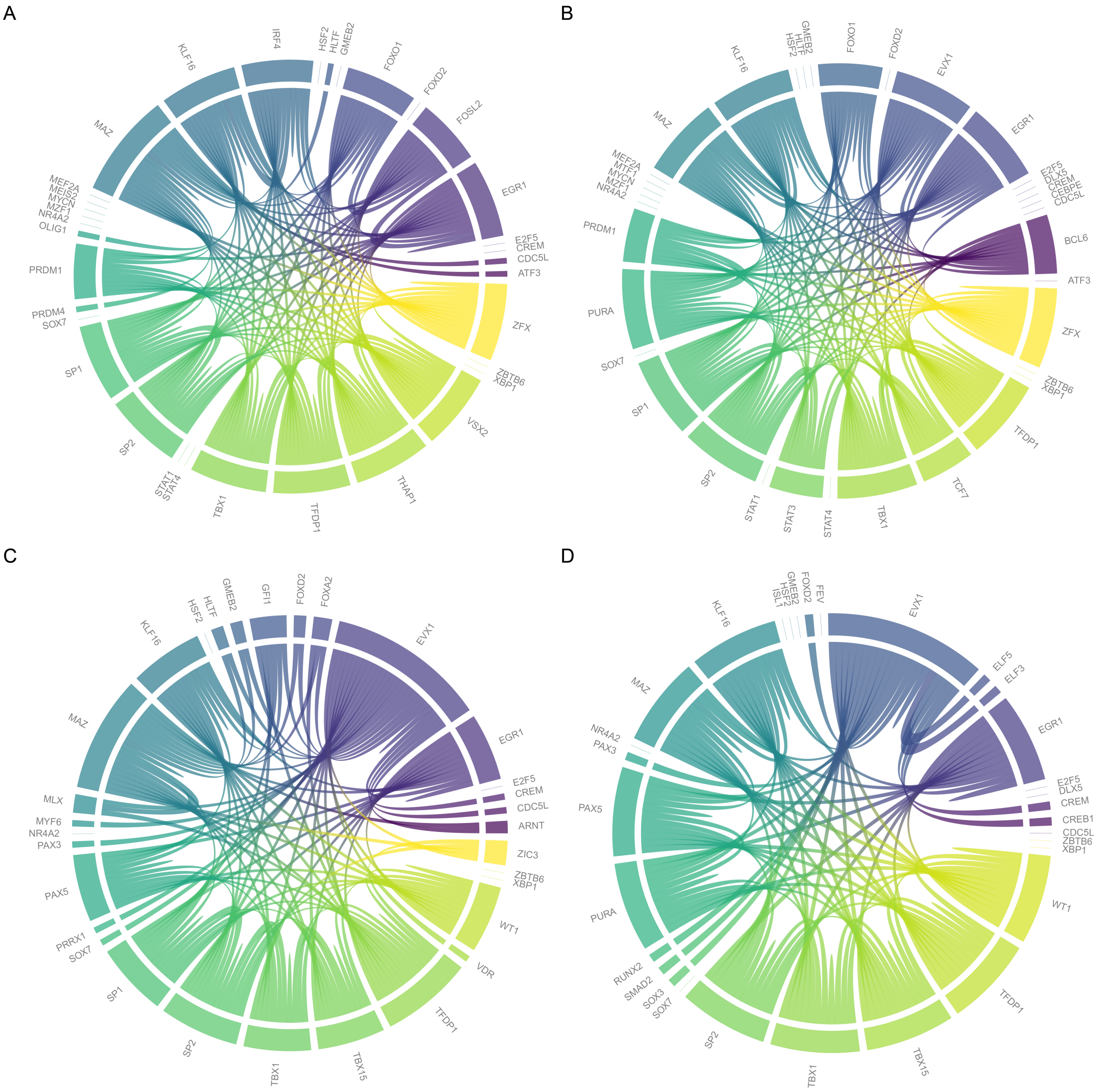

Fig. S27. Chord diagrams illustrate the transcription factor regulatory networks predicted by DeepTFni using metacells identified by EpiCarousel across Plasma cells **(A)**, Tcm **(B)**, CLP **(C)**, and Pro-B **(D)** in the PBBMC dataset.

Fig. S28

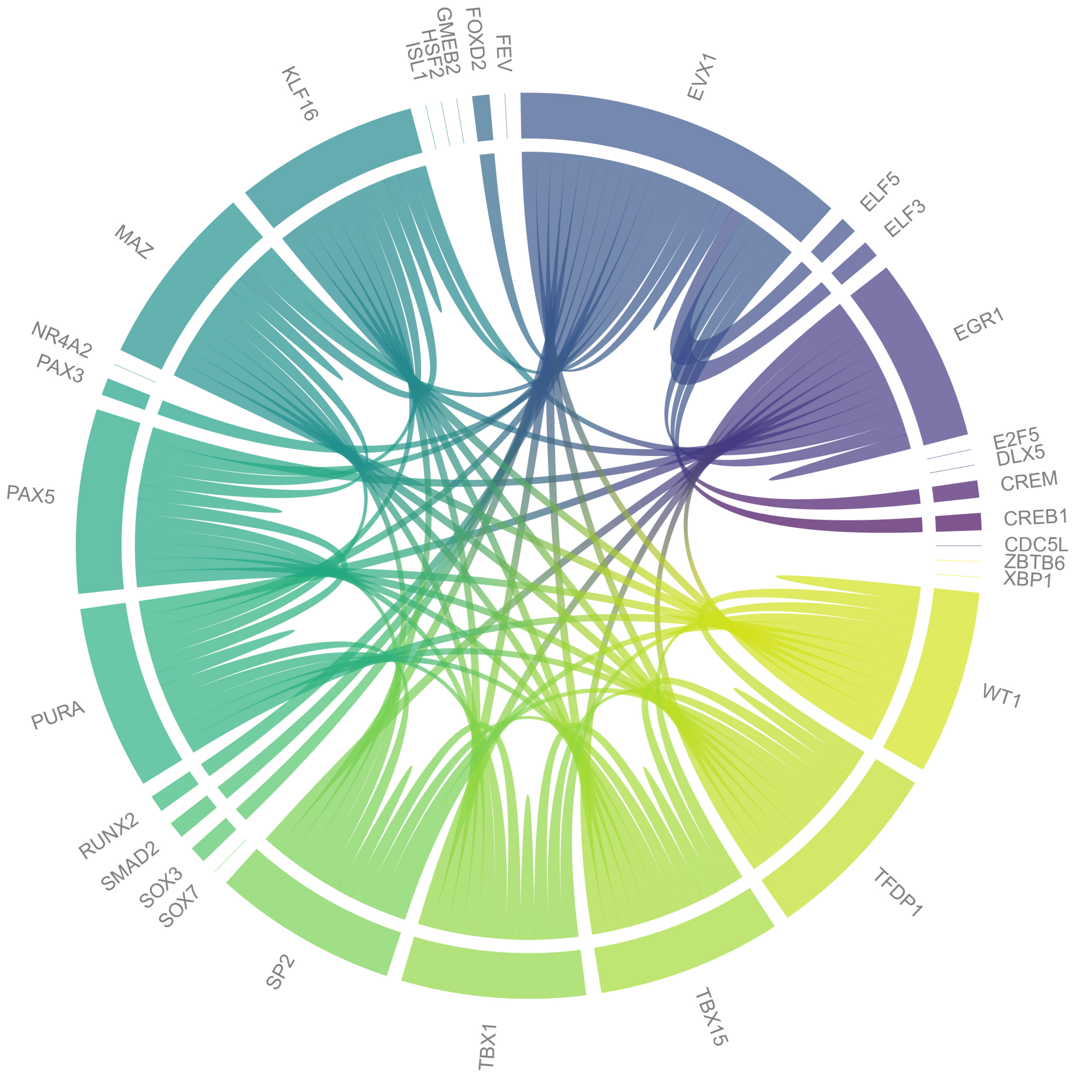

Fig. S28. Chord diagram illustrates the transcription factor regulatory networks predicted by DeepTFni using Th1 metacells identified by EpiCarousel in the BCC dataset.

Fig. S29

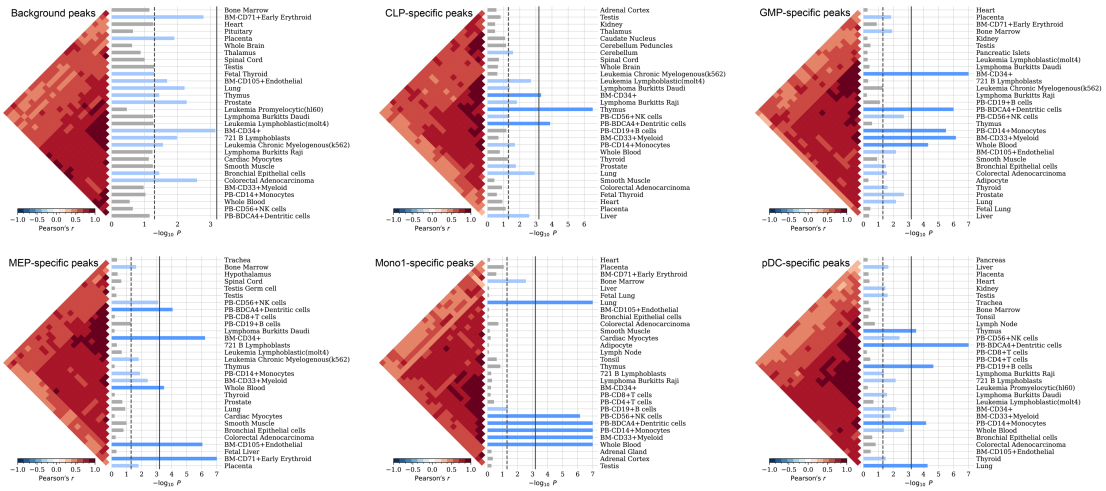

Fig. S29. Top 30 significantly enriched tissues in SNPsea analysis on the background peaks, CLP-specific peaks, GMP-specific peaks, MEP-specific peaks, Monocyte 1-specific peaks, and pDC-specific peaks, where 'Mono1' represents Monocyte 1. The vertical dashed and solid lines denote the one-sided P-value cutoff at the 0.05 level (unadjusted and with Bonferroni correction, respectively), testing the significance of gene enrichment for a specific annotation. The heatmaps show Pearson correlation coefficients for pairs of expression profiles ordered by hierarchical clustering with unweighted pair-group method with arithmetic means (UPGMA).

Fig. S30

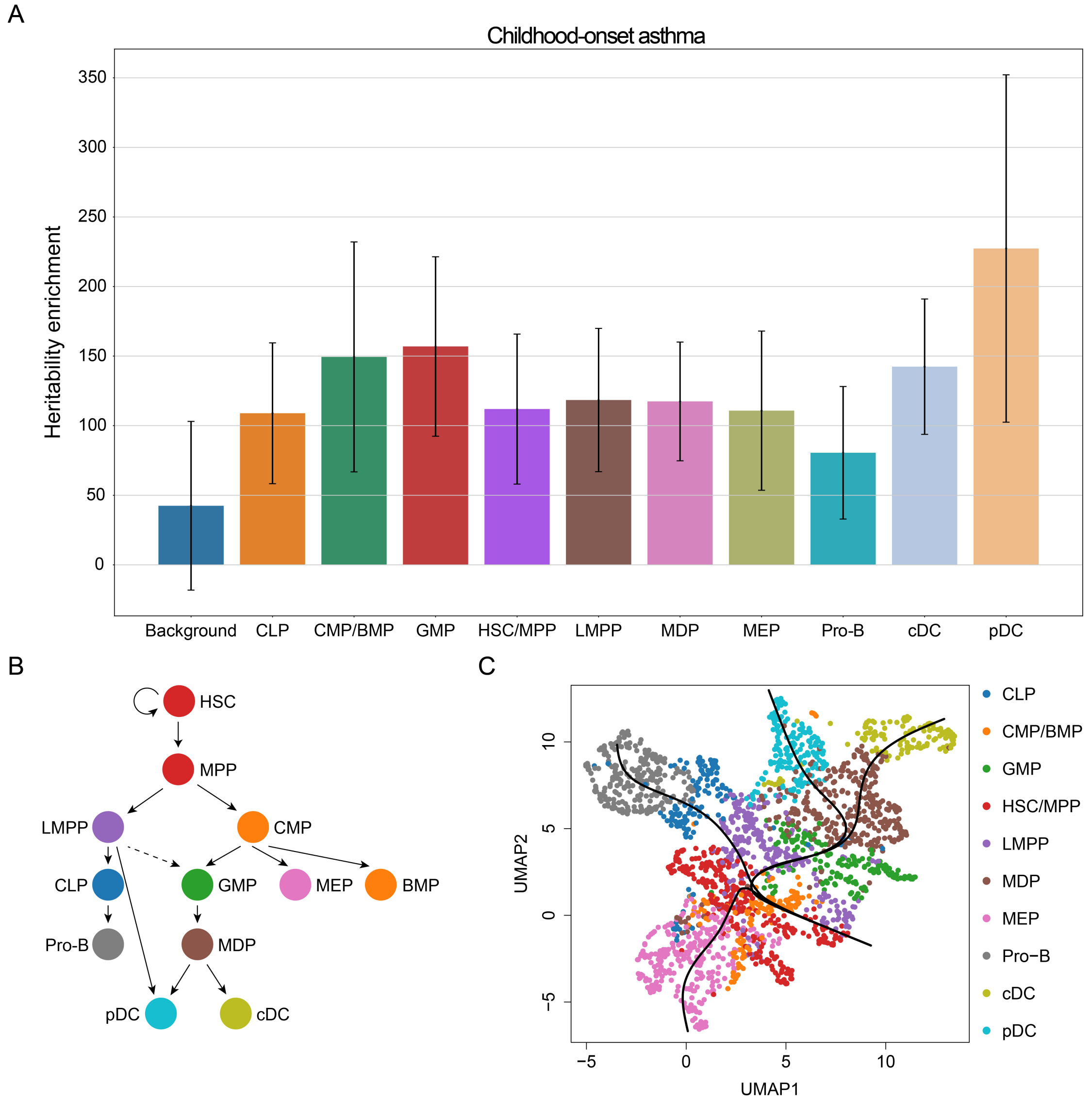

Fig. S30. (A) Heritability enrichment estimates for the cell type-specific peaks of metacells identified by EpiCarousel and the background peaks for childhood-onset asthma. The error bars and centres of error bars represent jackknife standard errors and average values over 200 equally sized blocks of adjacent SNPs about the estimates of enrichment, respectively. **(B)** A schematic of human hematopoiesis differentiation lineage for the Immune dataset. **(C)** UMAP visualization of the metacells from the Immune dataset and the inferred trajectory with Slingshot.

Fig. S31

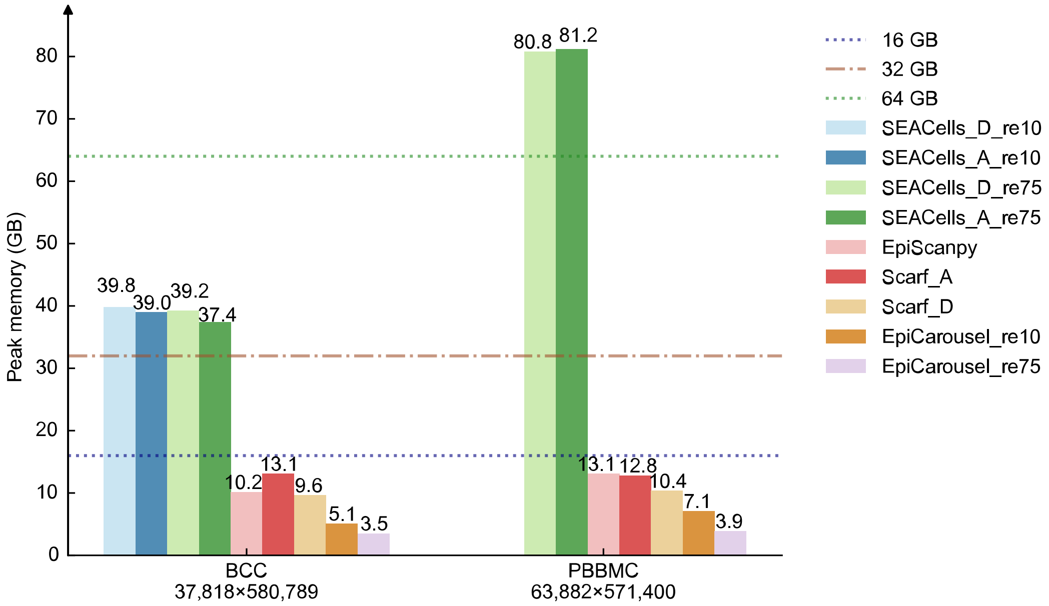

Fig. S31. Plot of the peak memory (in GB) required of each method for benchmarking on BCC and PBBMC datasets. The horizontal axis represents different data categories, with the number of cells and dimensions annotated below each data label.

Fig. S32

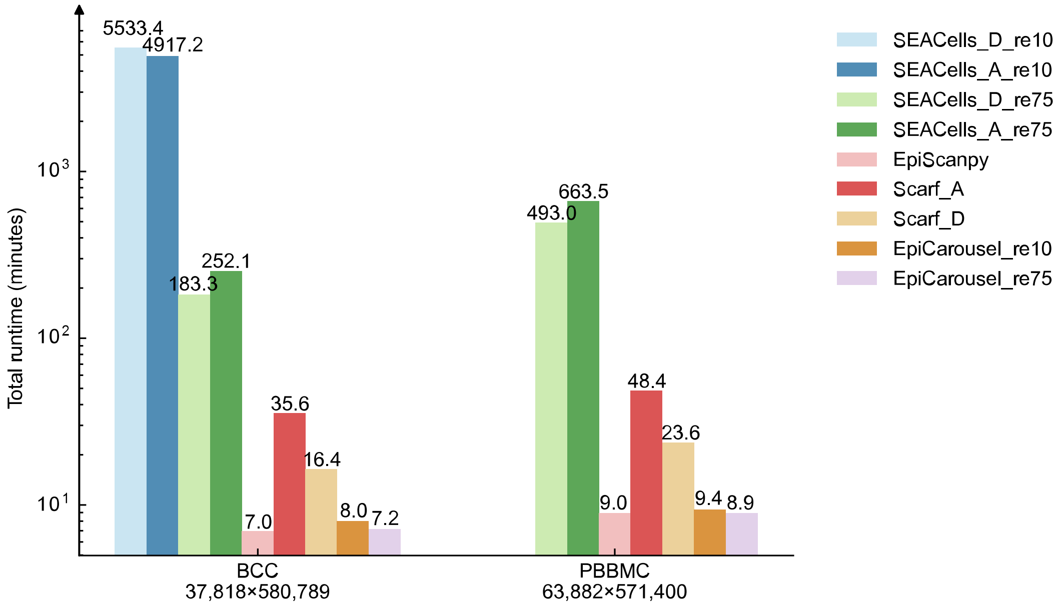

Fig. S32. Plot of total runtime (in minutes) for benchmarking each method on BCC and PBBMC datasets. The horizontal axis represents different data categories, with the number of cells and dimensions annotated below each data label.

Fig. S33

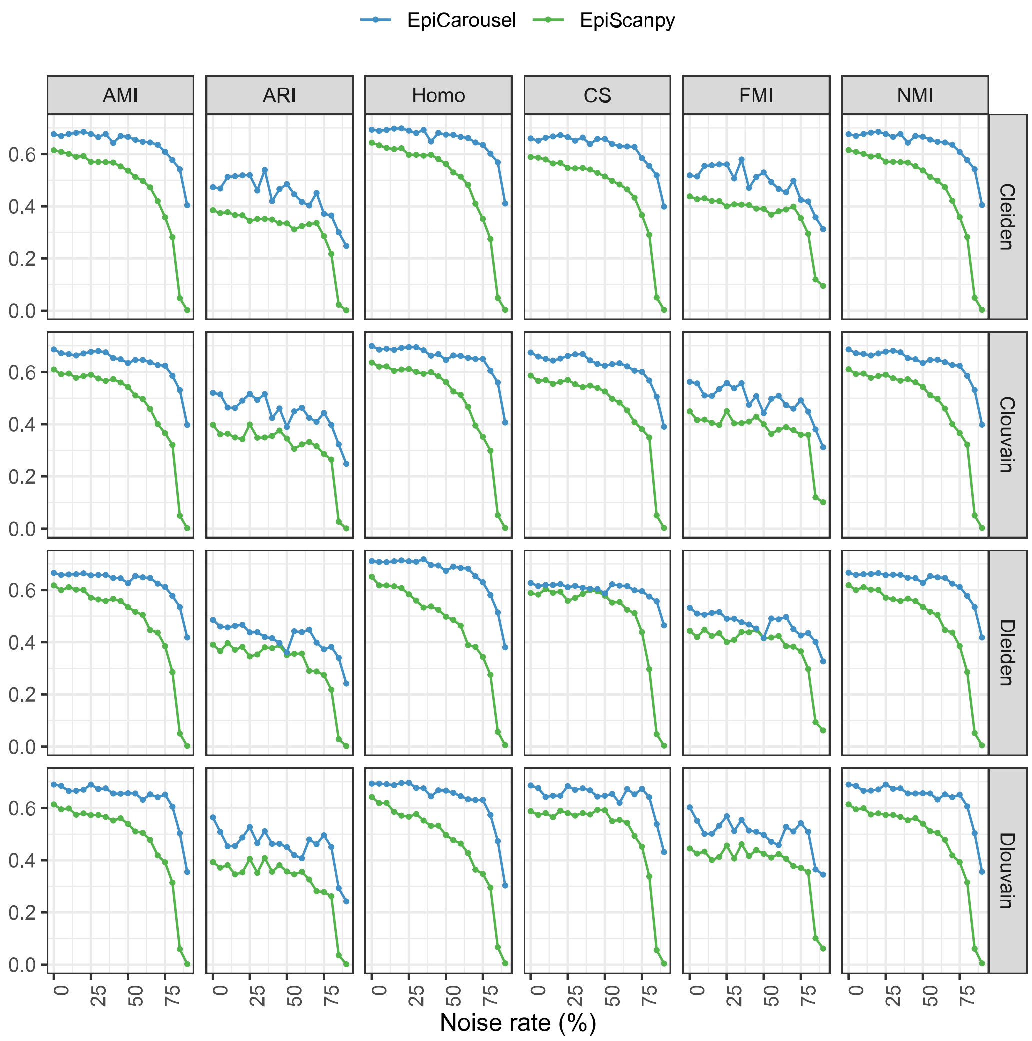

Fig. S33. Line plots illustrate the clustering performance of EpiCarousel and EpiScanpy using 4 clustering strategies on the BMMC dataset, which introduces varying degrees of noise. The noise level ranges from 0 to 90, with an increment of 5 for each step.

Fig. S34

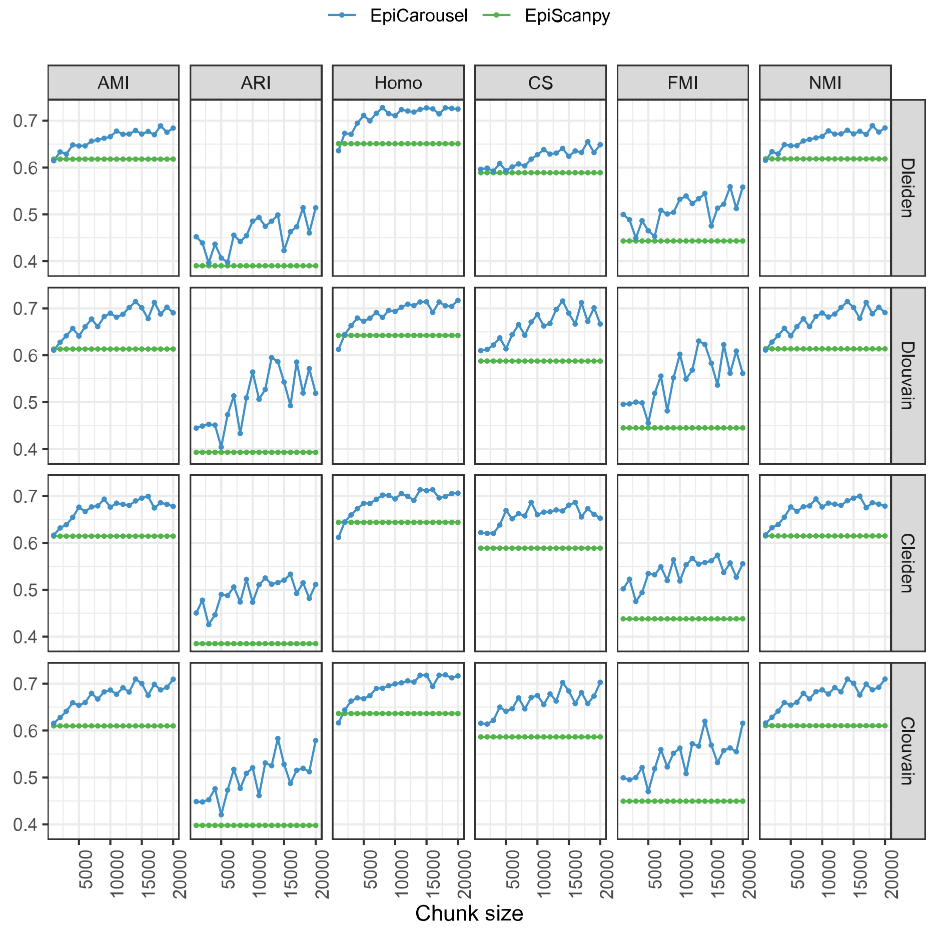

Fig. S34. Line plots demonstrate the robustness of EpiCarousel to chunk size, evaluated on the BMMC dataset using the 4 clustering strategies. The chunk size ranges from 1000 to 20,000, with an increment of 1000 for each step.

Fig. S35

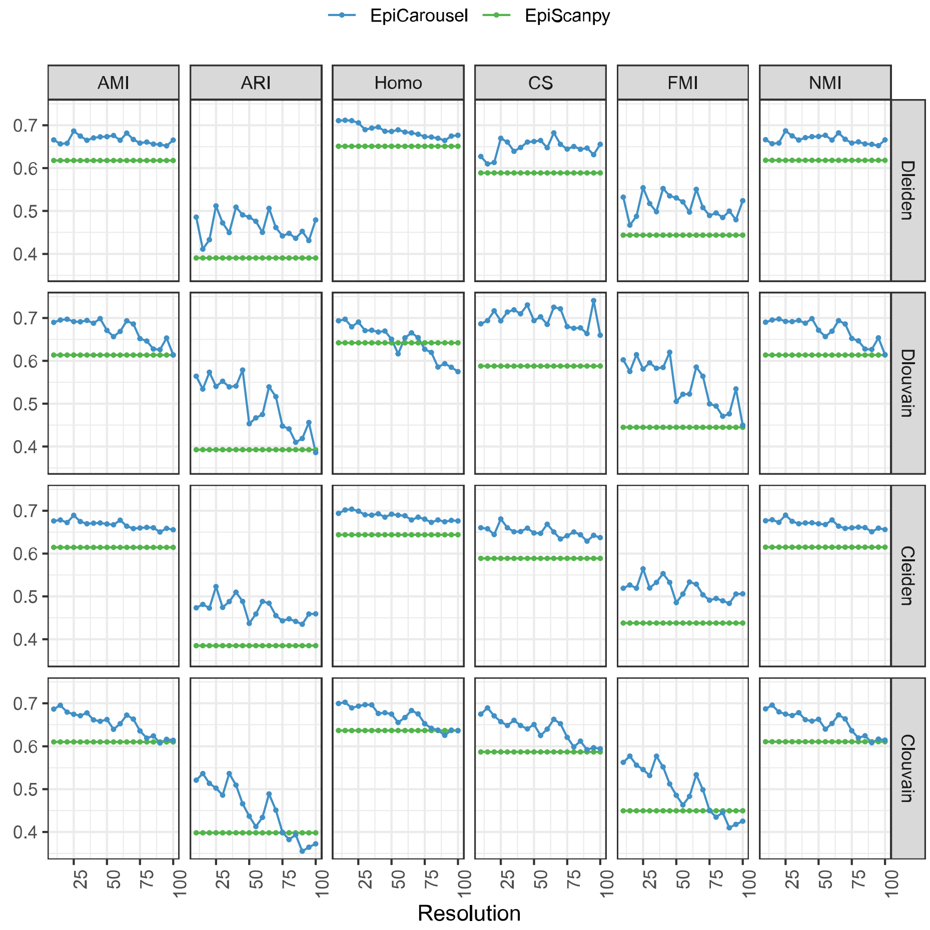

Fig. S35. Line plots demonstrate the robustness of EpiCarousel to resolution, evaluated on the BMMC dataset using the 4 clustering strategies. The resolution ranges from 10 to 100, with an increment of 5 for each step.

Fig. S36

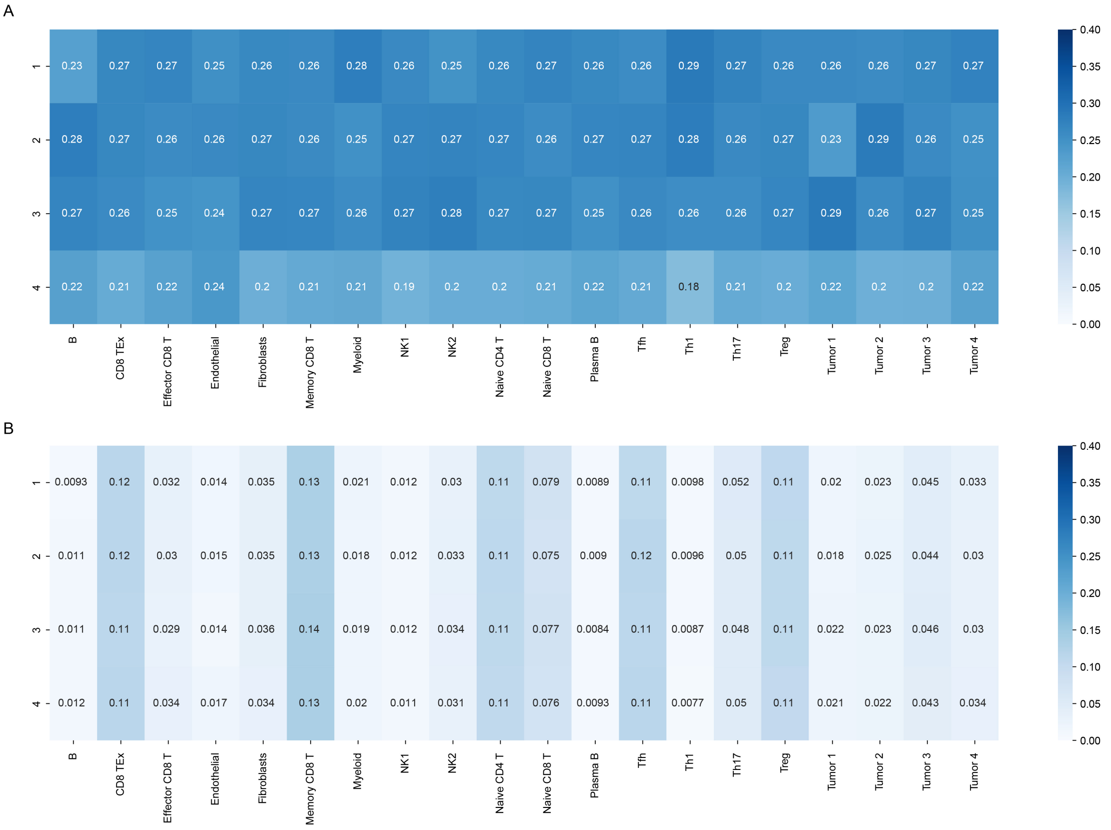

Fig. S36. The distribution of each cell type across different chunks **(A)** and the proportion of each cell type within each chunk **(B)** of BCC.

Fig. S37

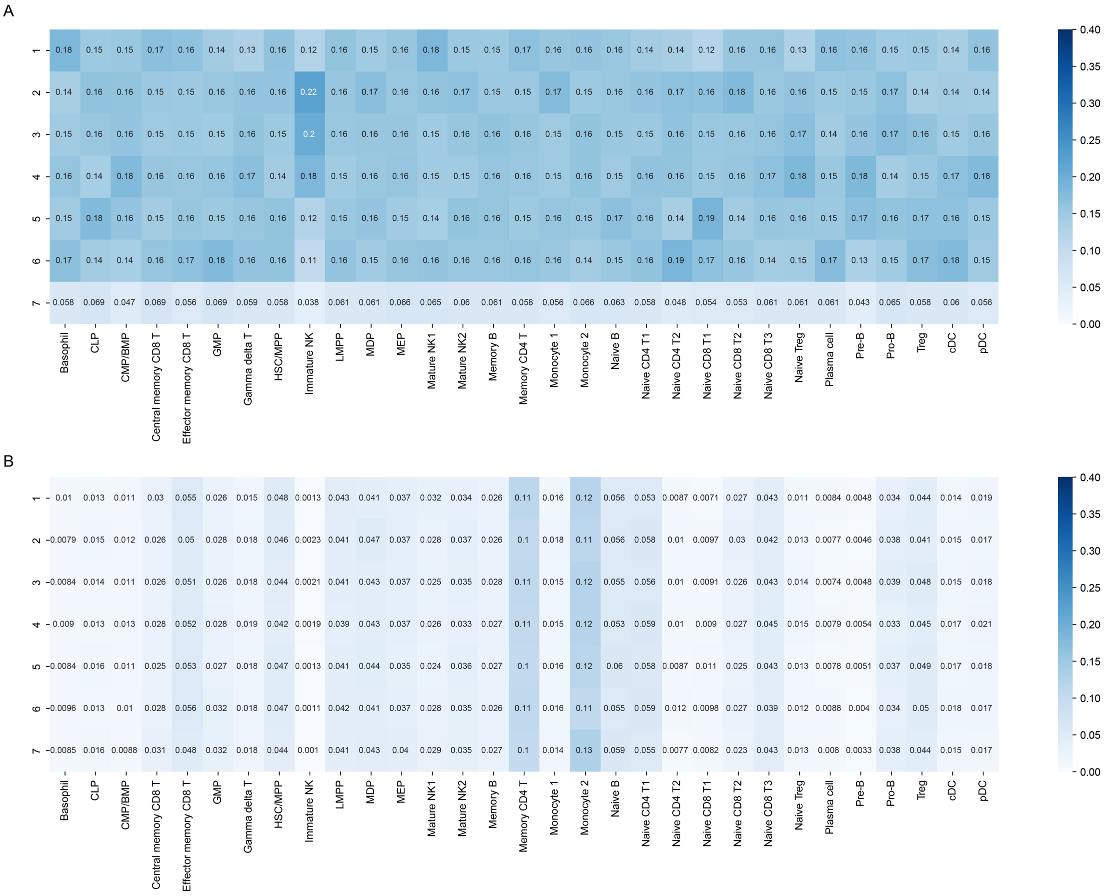

Fig. S37. The distribution of each cell type across different chunks **(A)** and the proportion of each cell type within each chunk **(B)** of PBBMC.

Fig. S38

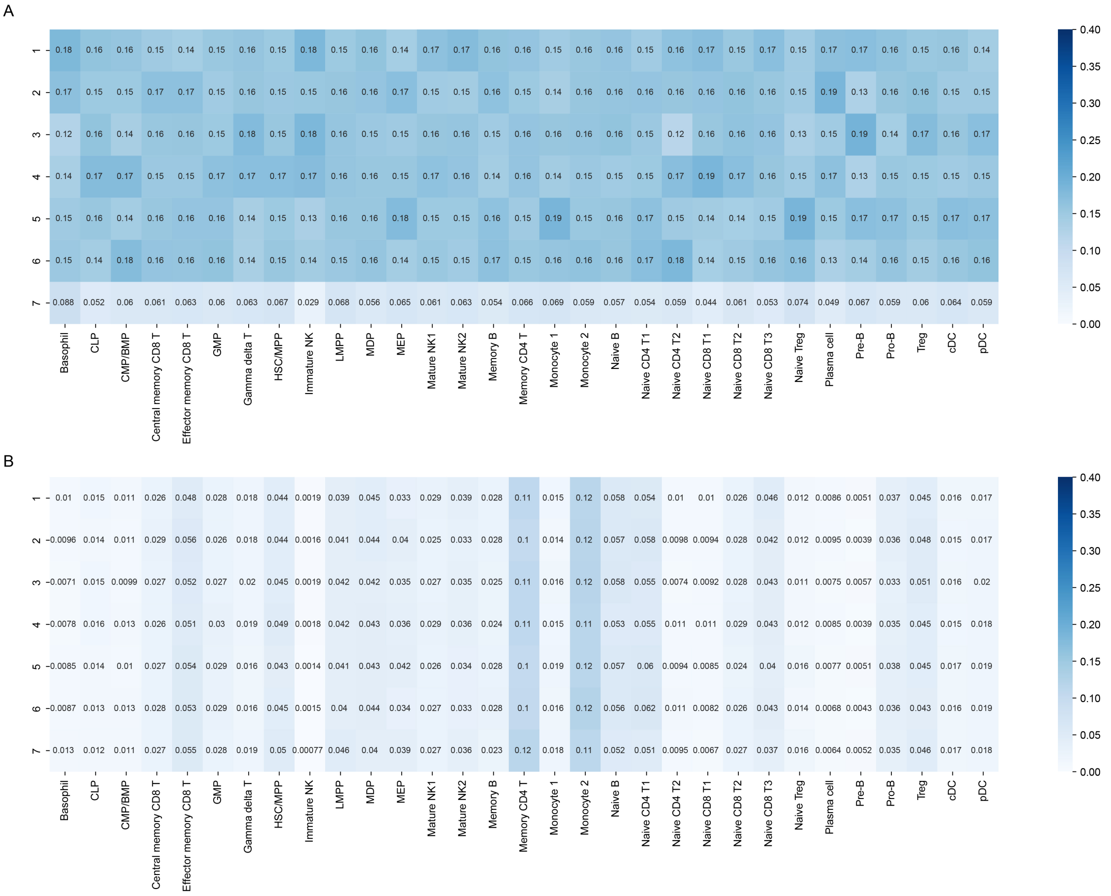

Fig. S38. The distribution of each cell type across different chunks **(A)** and the proportion of each cell type within each chunk **(B)** of the PBBMC dataset shuffled with random seed 301.

Fig. S39

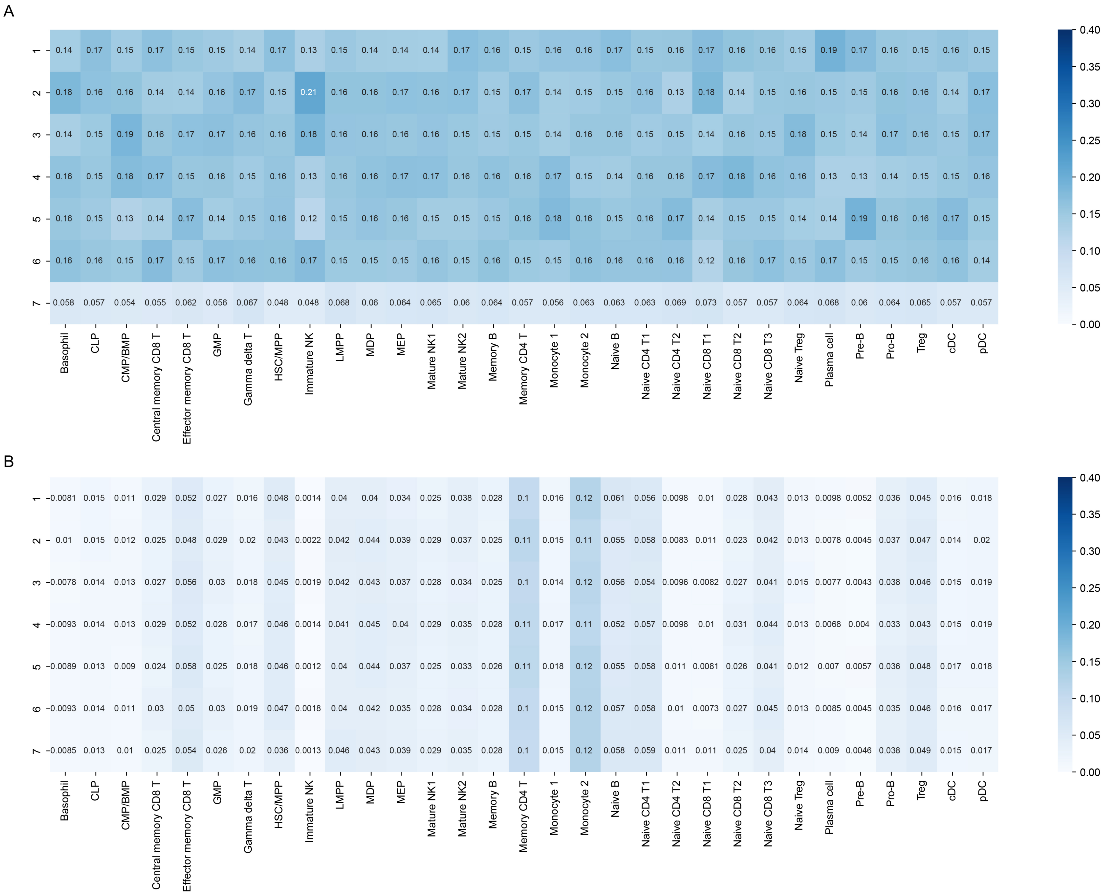

Fig. S39. The distribution of each cell type across different chunks **(A)** and the proportion of each cell type within each chunk **(B)** of the PBBMC dataset shuffled with random seed 1888.

Fig. S40

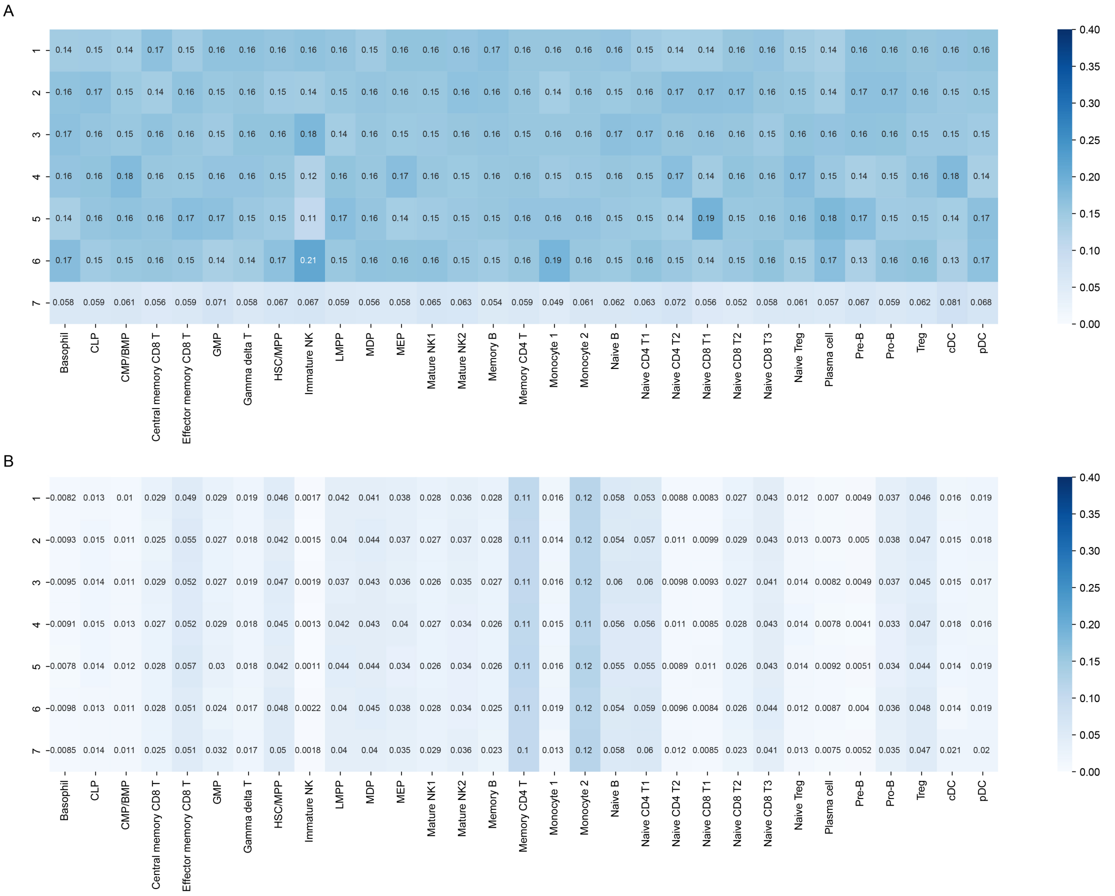

Fig. S40. The distribution of each cell type across different chunks **(A)** and the proportion of each cell type within each chunk **(B)** of the PBBMC dataset shuffled with random seed 3407.

Fig. S41

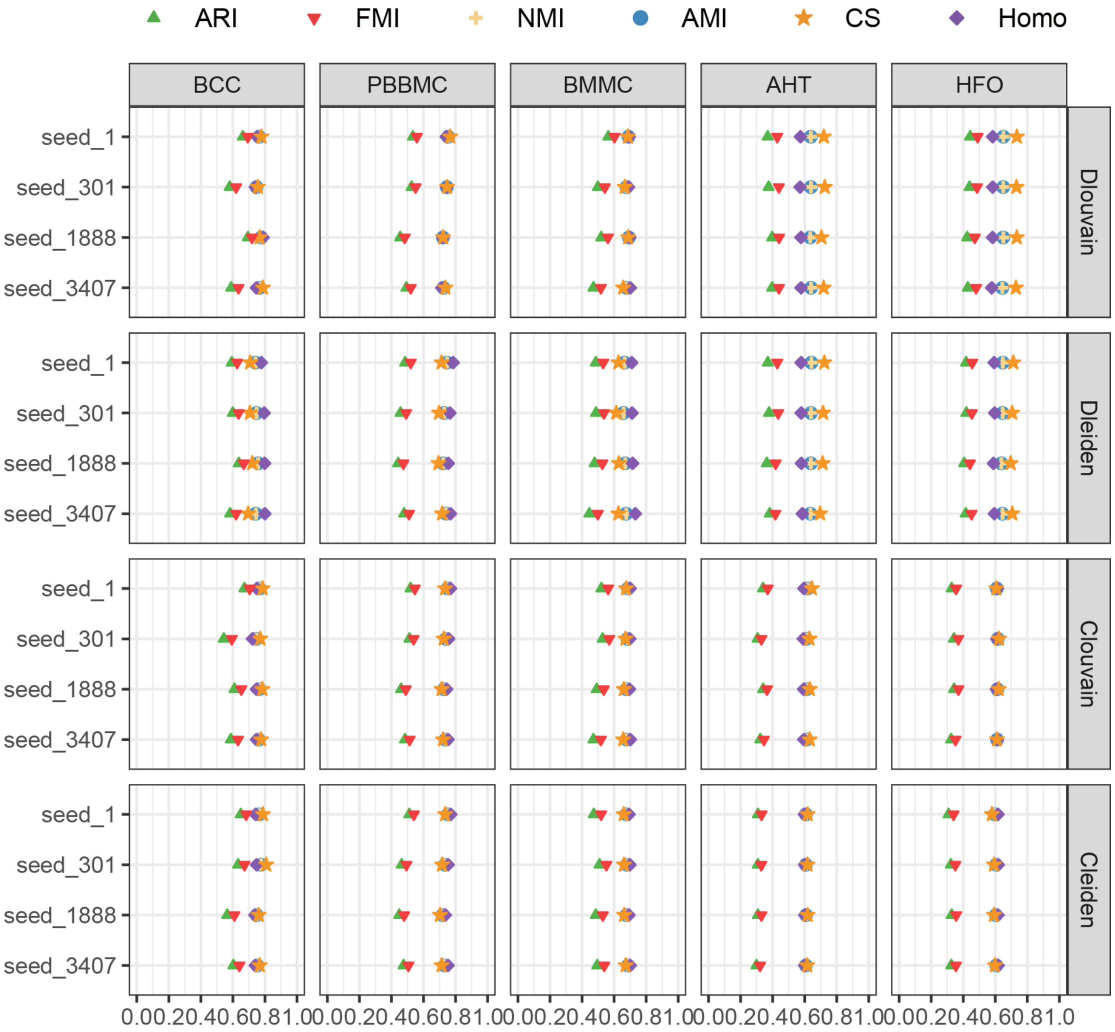

Fig. S41. Clustering performance of EpiCarousel on datasets shuffled with random seeds 1, 301, 1888, and 3407, respectively.

Fig. S42

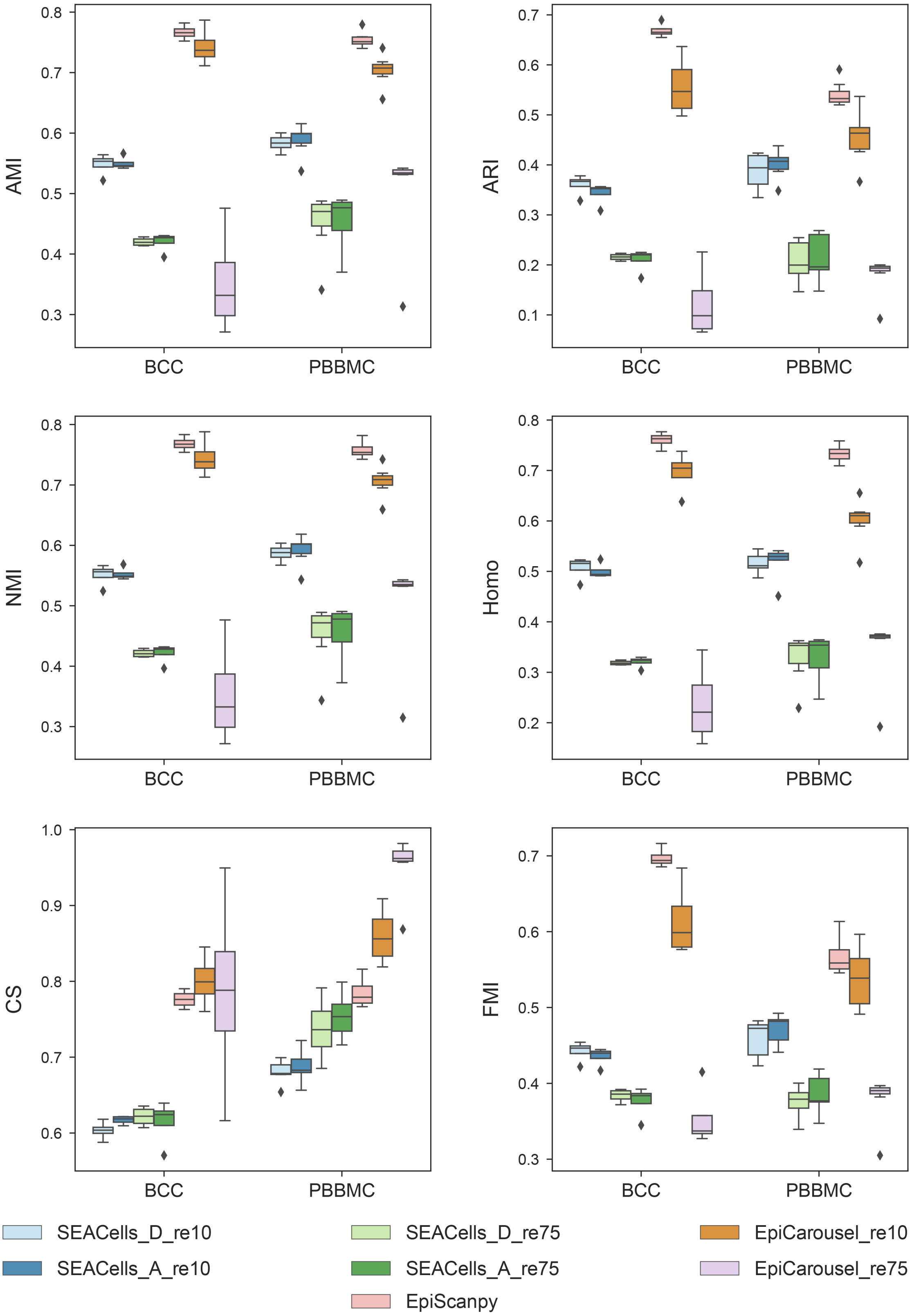

Fig. S42. Dlouvain clustering performance on chunks of the BCC and PBBMC datasets.

Fig. S43

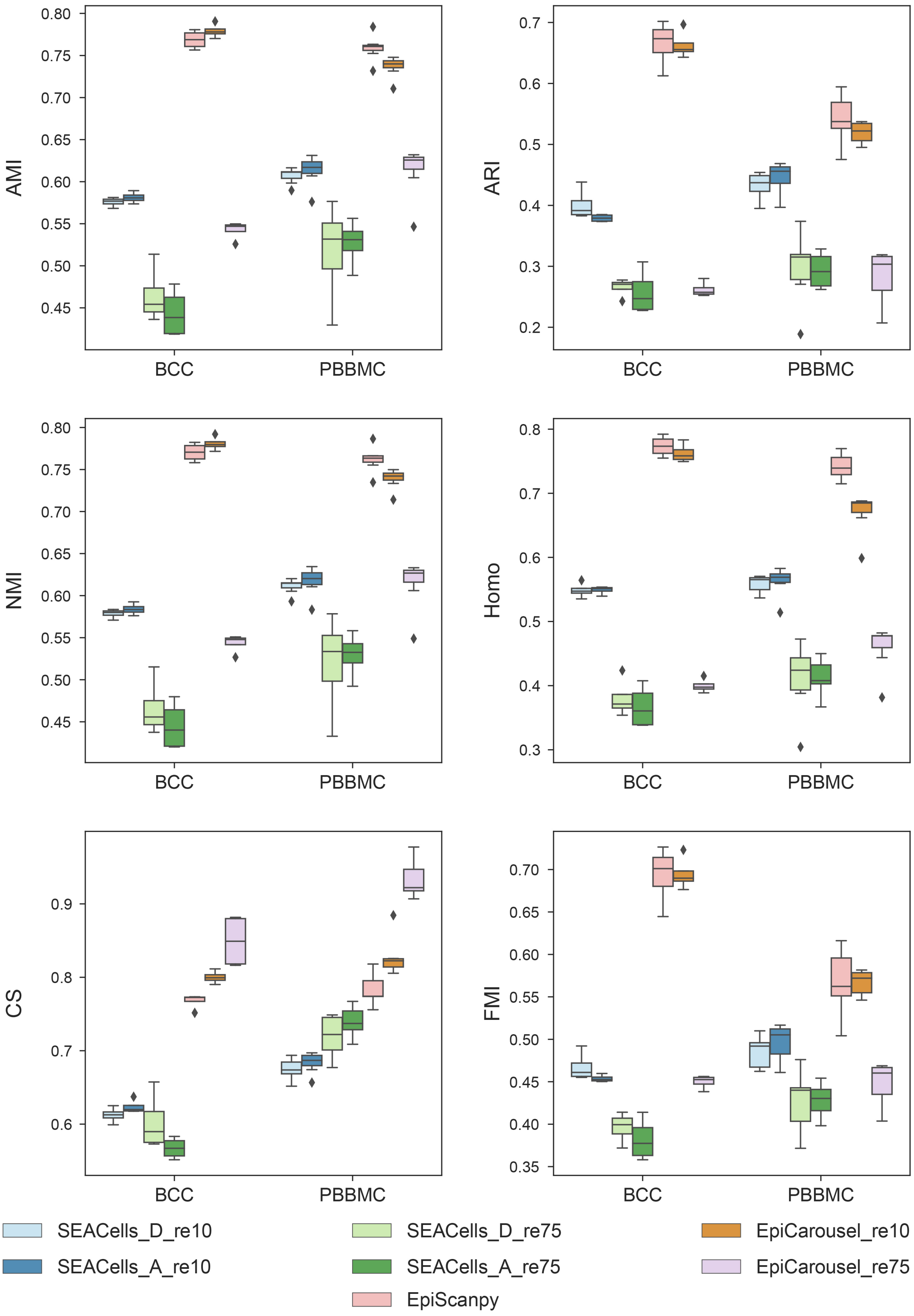

Fig. S43. Dleiden clustering performance on chunks of the BCC and PBBMC datasets.

Fig. S44

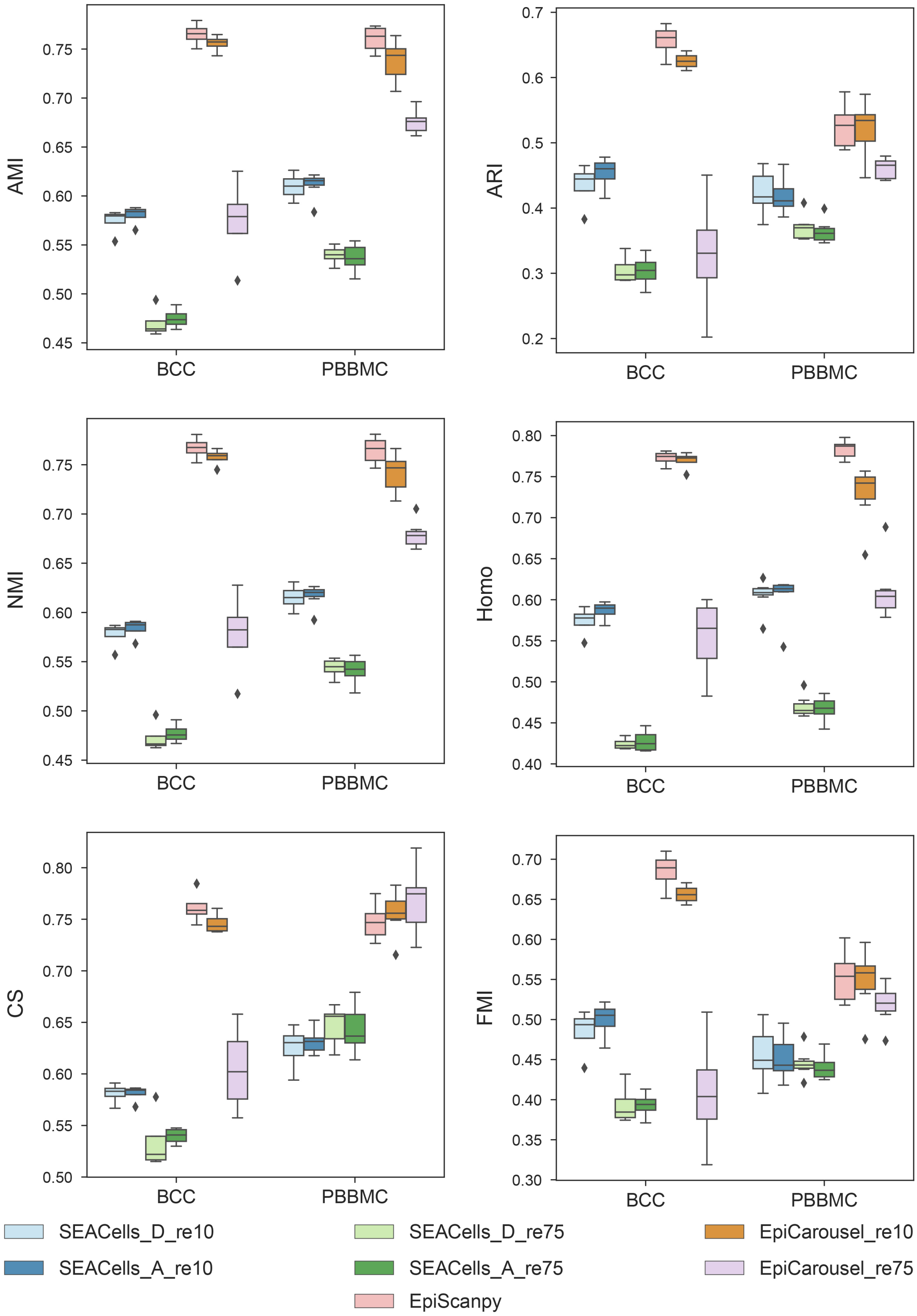

Fig. S44. Clouvain clustering performance on chunks of the BCC and PBBMC datasets.

Fig. S45

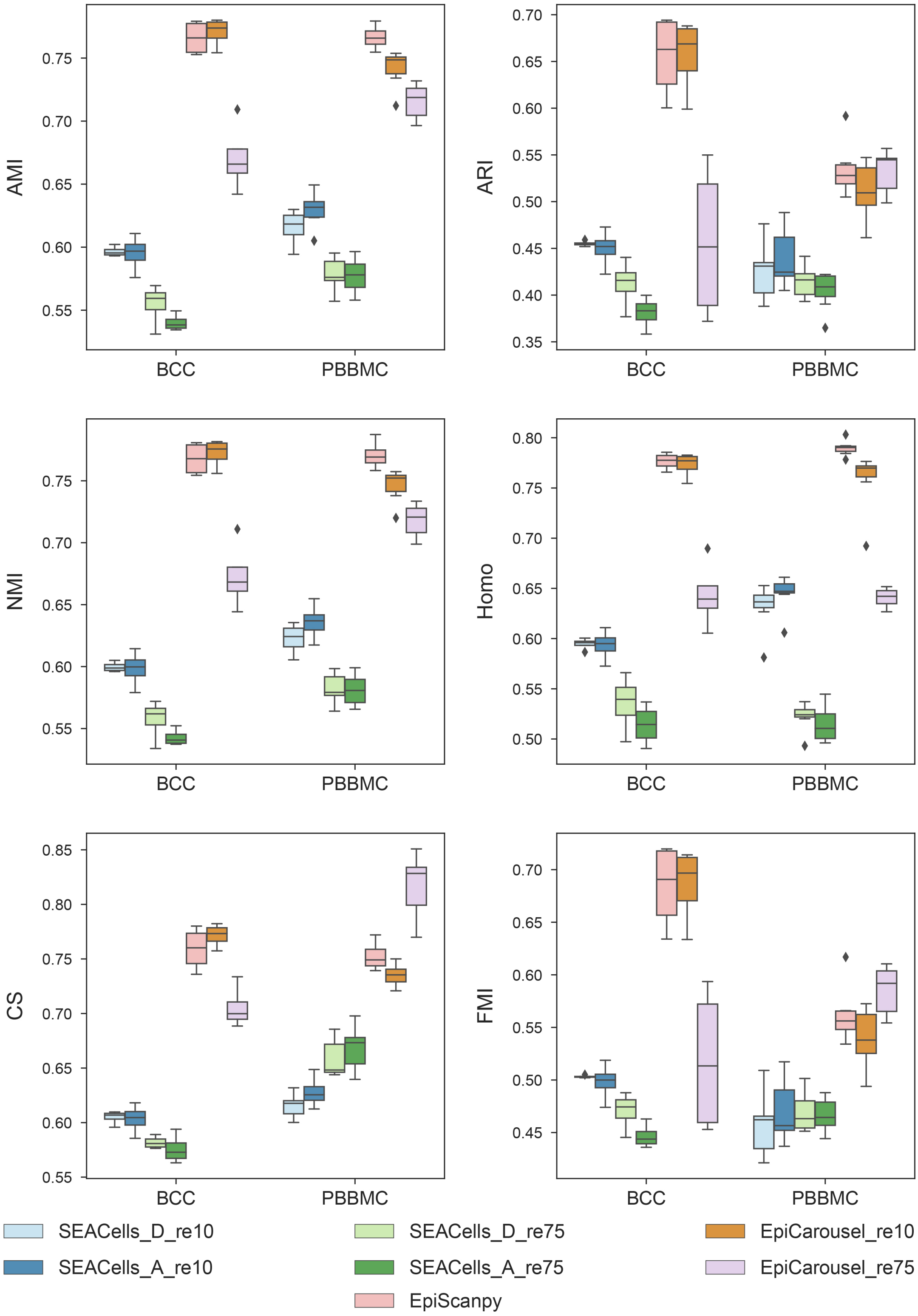

Fig. S45. Cleiden clustering performance on chunks of the BCC and PBBMC datasets.

Fig. S46

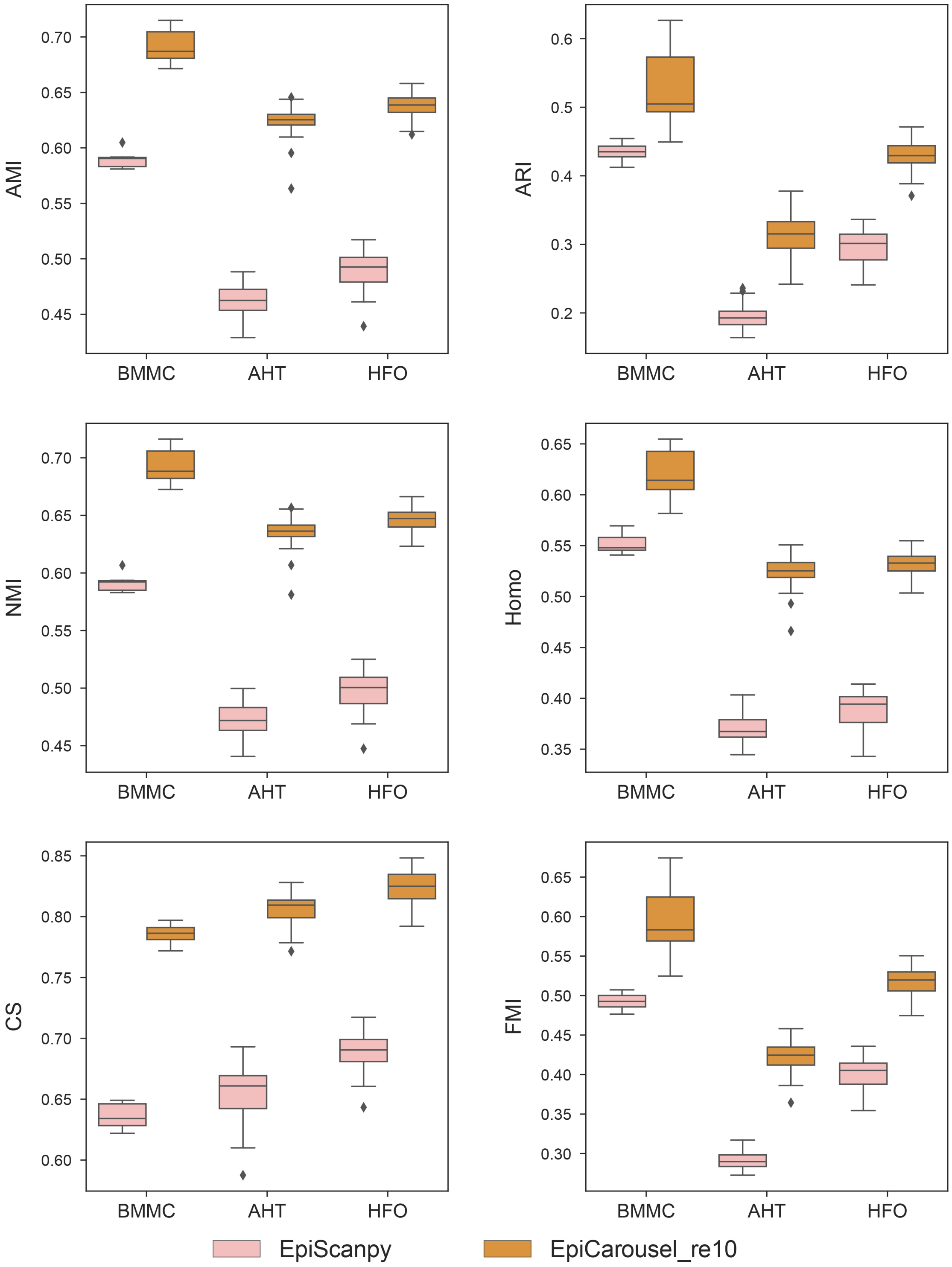

Fig. S46. Dlouvain clustering performance on chunks of the BMMC, AHT, and HFO datasets.

Fig. S47

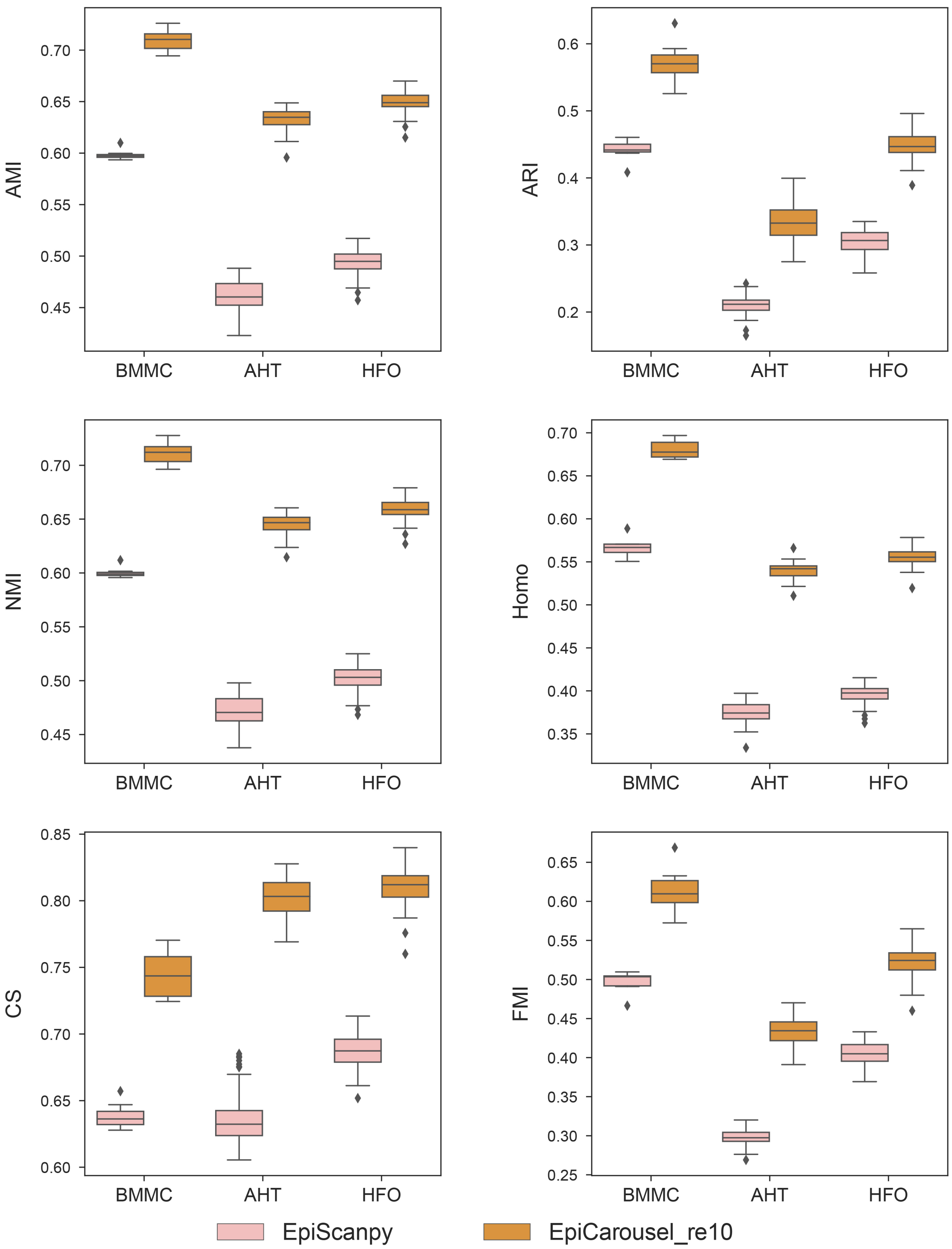

Fig. S47. Dleiden clustering performance on chunks of the BMMC, AHT, and HFO datasets.

Fig. S48

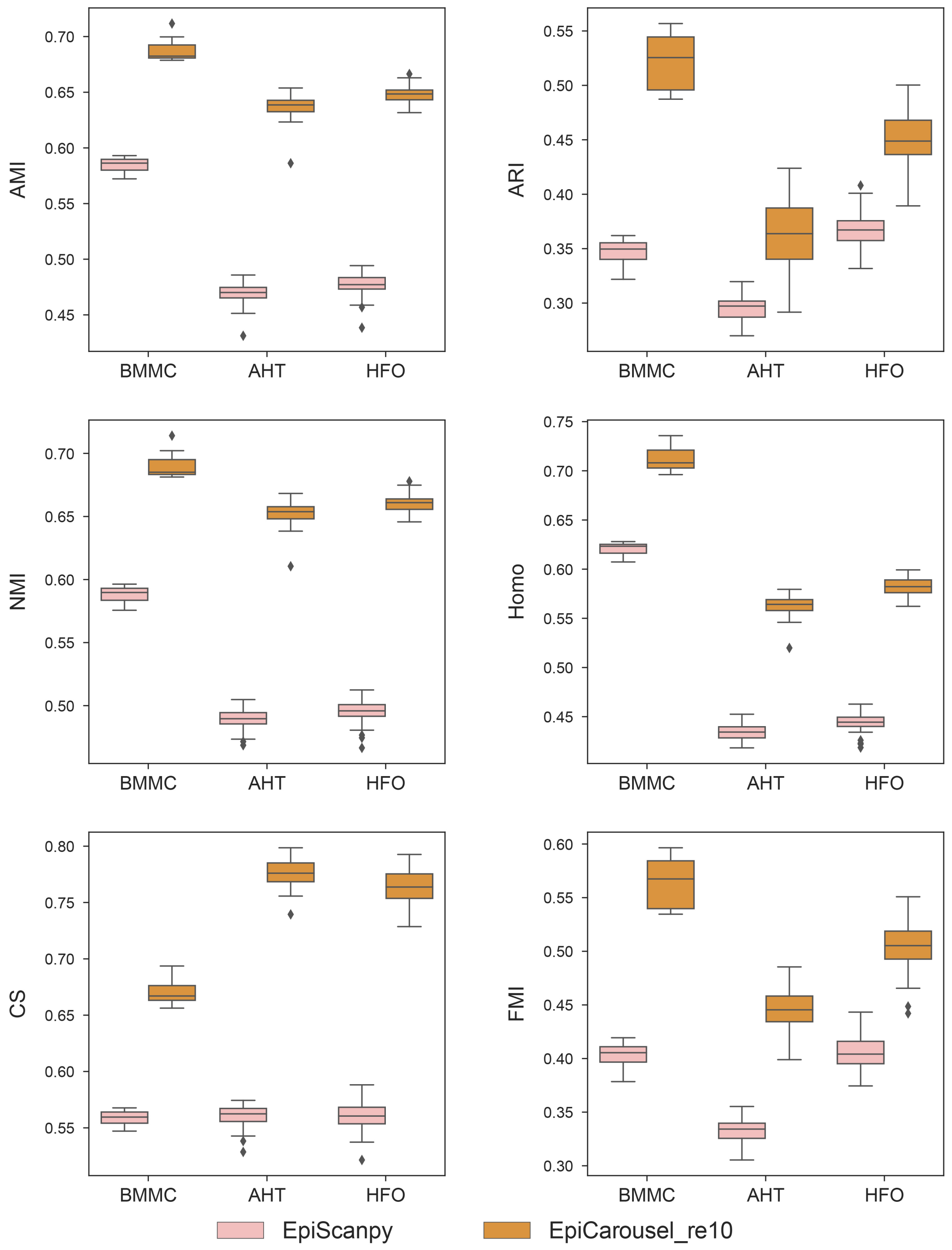

Fig. S48. Clouvain clustering performance on chunks of the BMMC, AHT, and HFO datasets.

Fig. S49

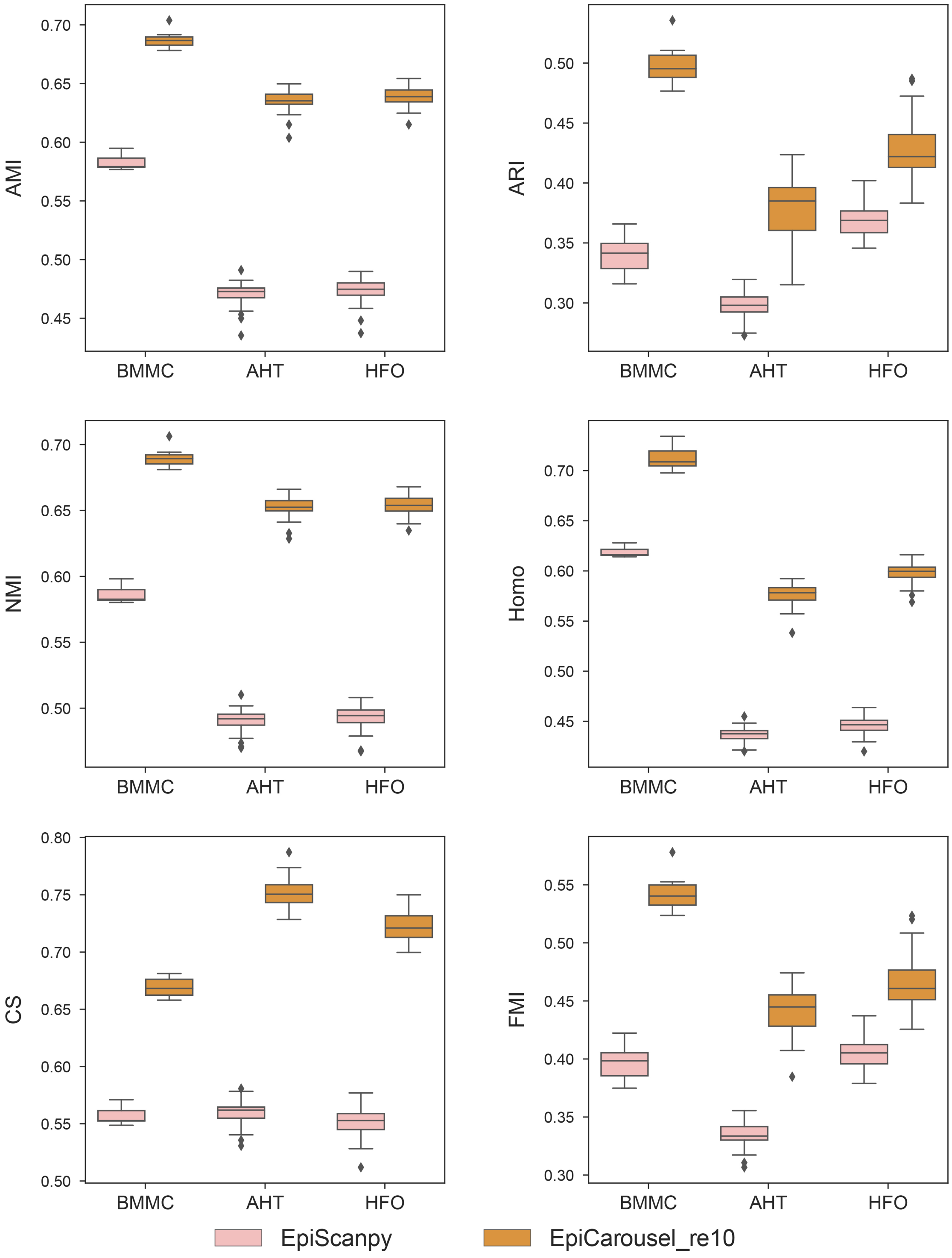

Fig. S49. Cleiden clustering performance on chunks of the BMMC, AHT, and HFO datasets.

Fig. S50

A

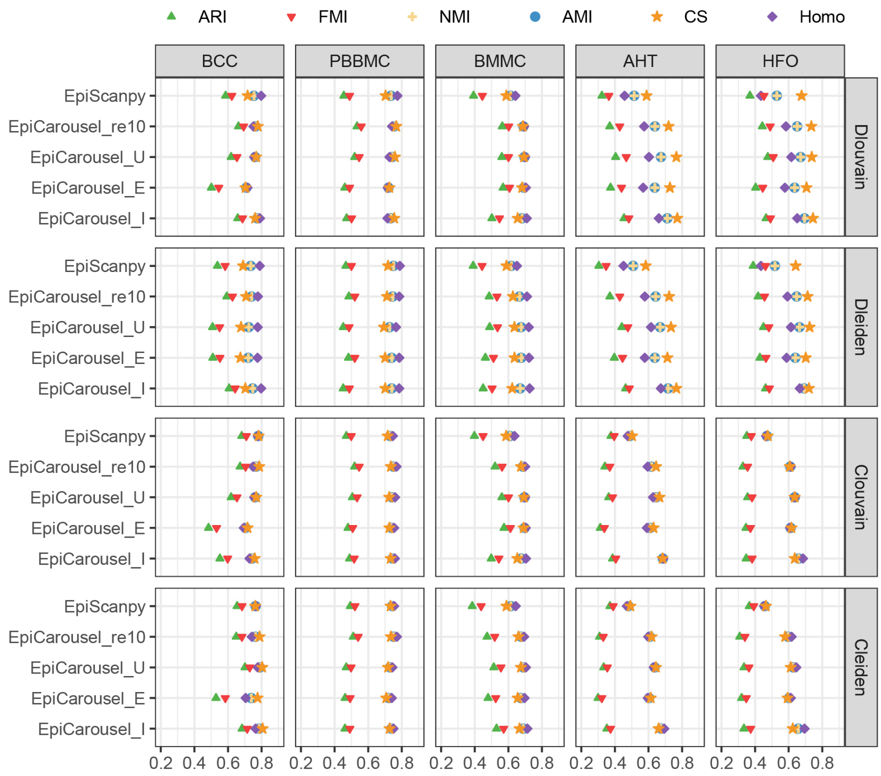

B

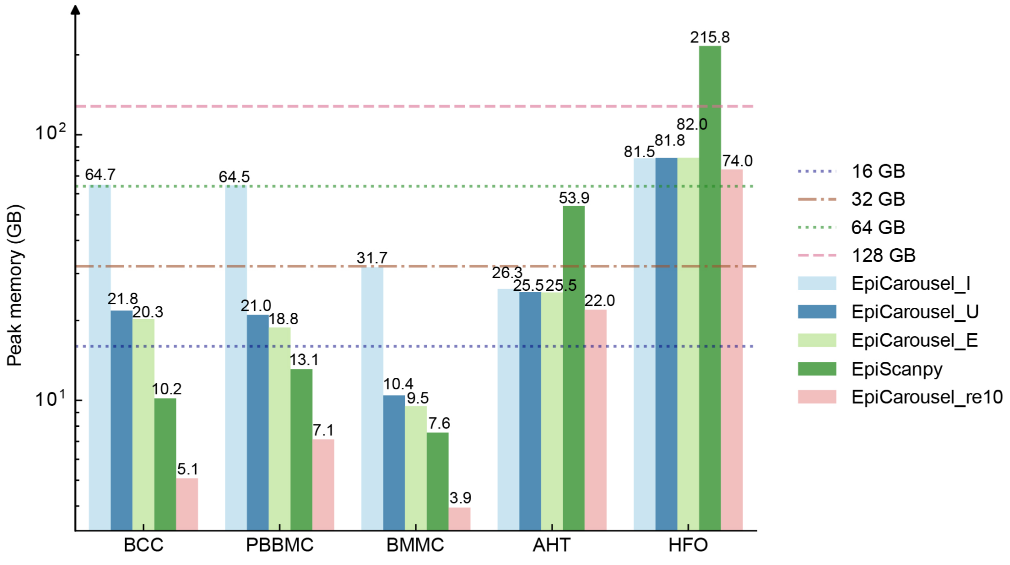

Fig. S50. Benchmarking results of various methods. (A) Clustering performance of different methods across various datasets. (B) Plot of the peak memory usage of each method for benchmarking on various datasets.

Fig. S51

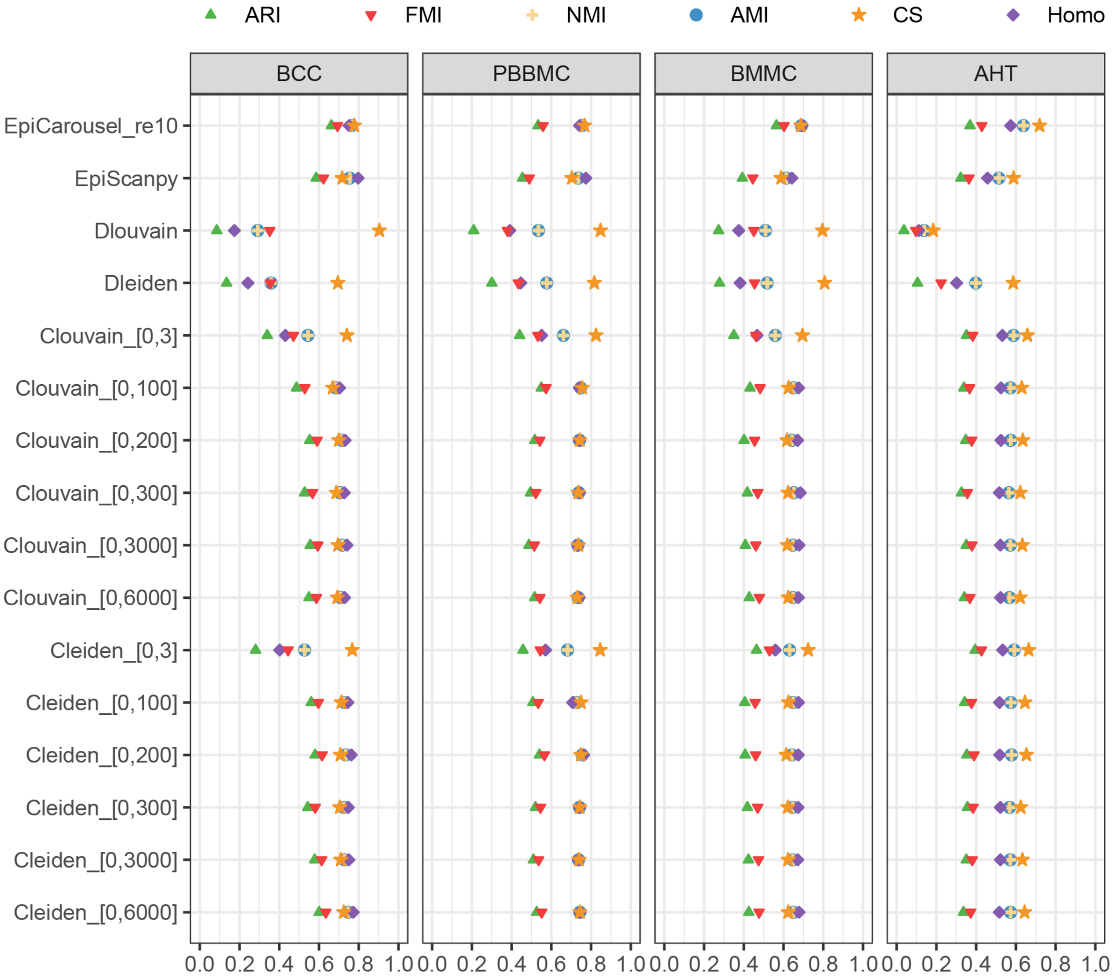

Fig. S51. Dot plots showing the clustering results of various methods using Dlouvain on individual datasets. The scale of the dataset increases from left to right.

Fig. S52

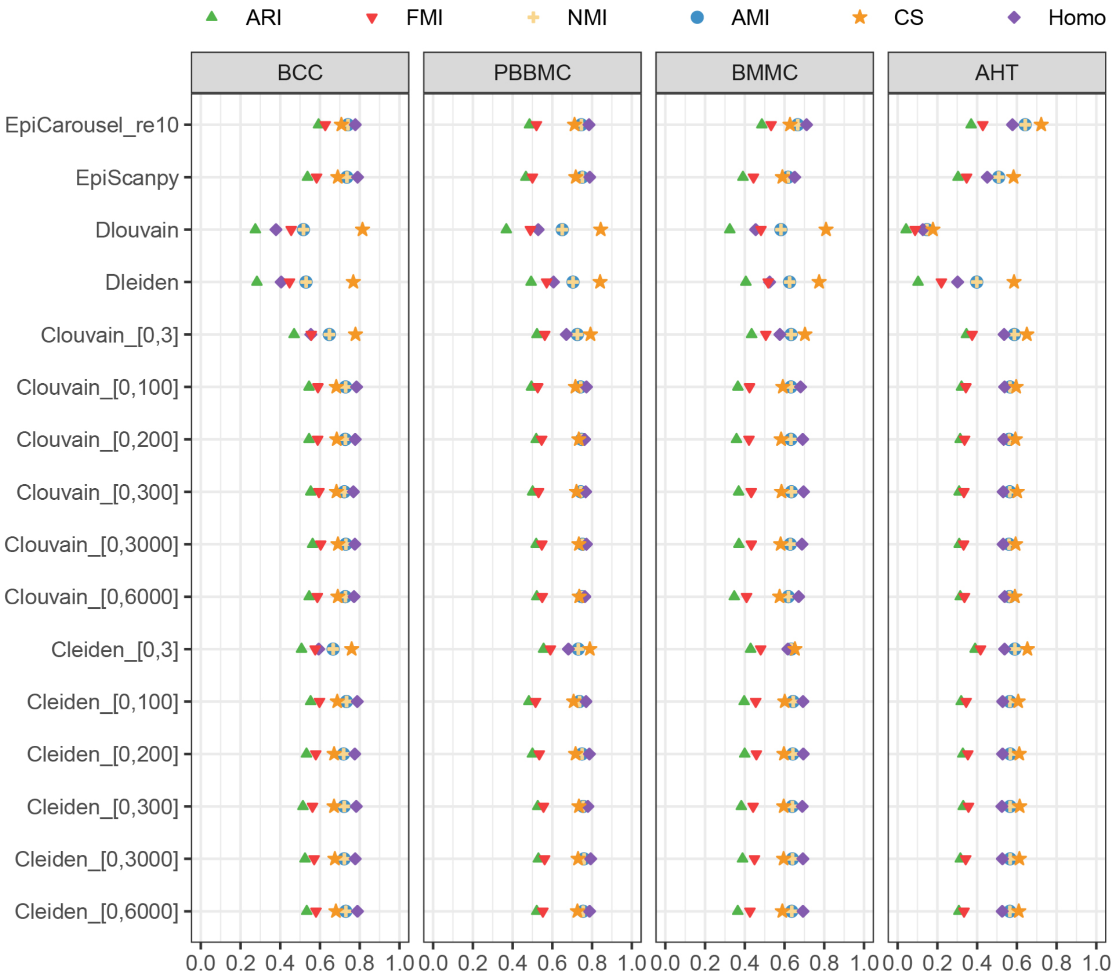

Fig. S52. Dot plots showing the clustering results of various methods using Dleiden on individual datasets. The scale of the dataset increases from left to right.

Fig. S53

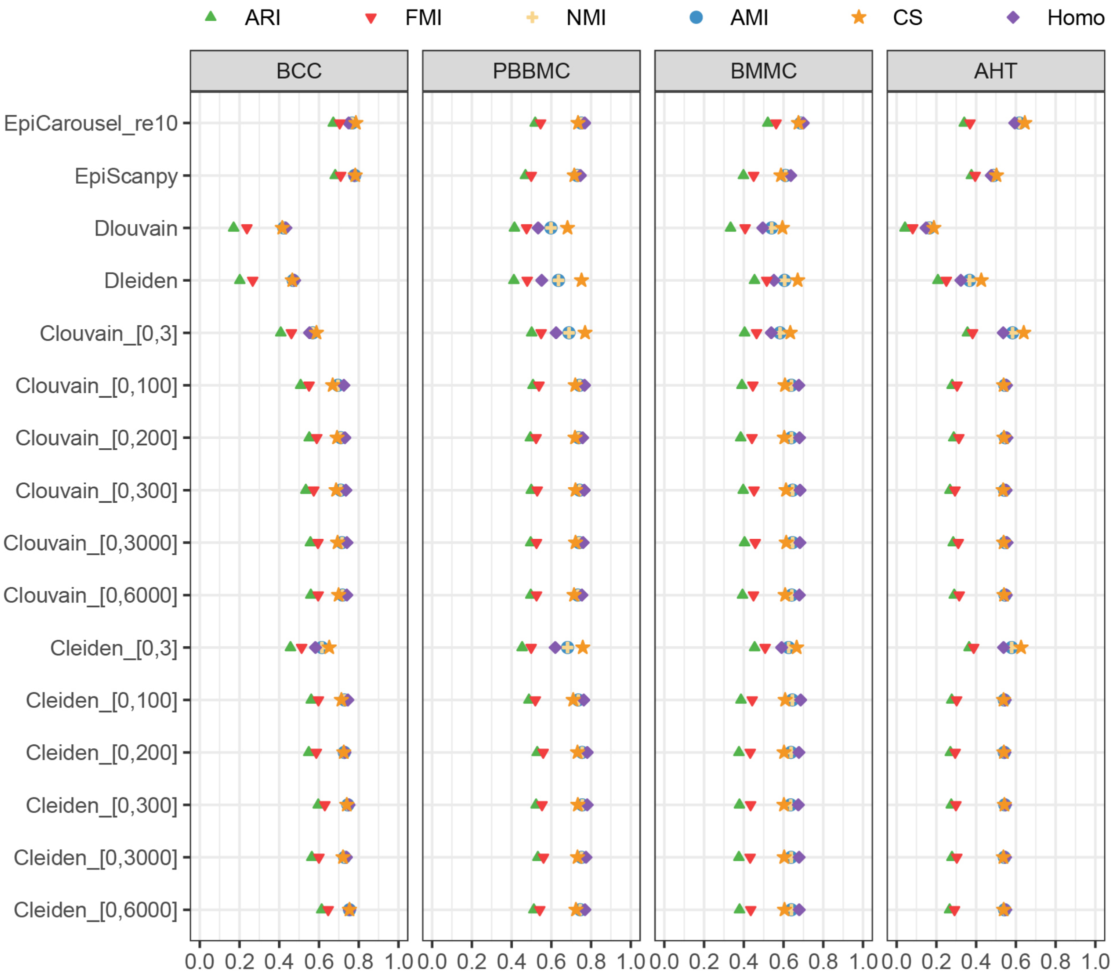

Fig. S53. Dot plots showing the clustering results of various methods using Clouvain on individual datasets. The scale of the dataset increases from left to right.

Fig. S54

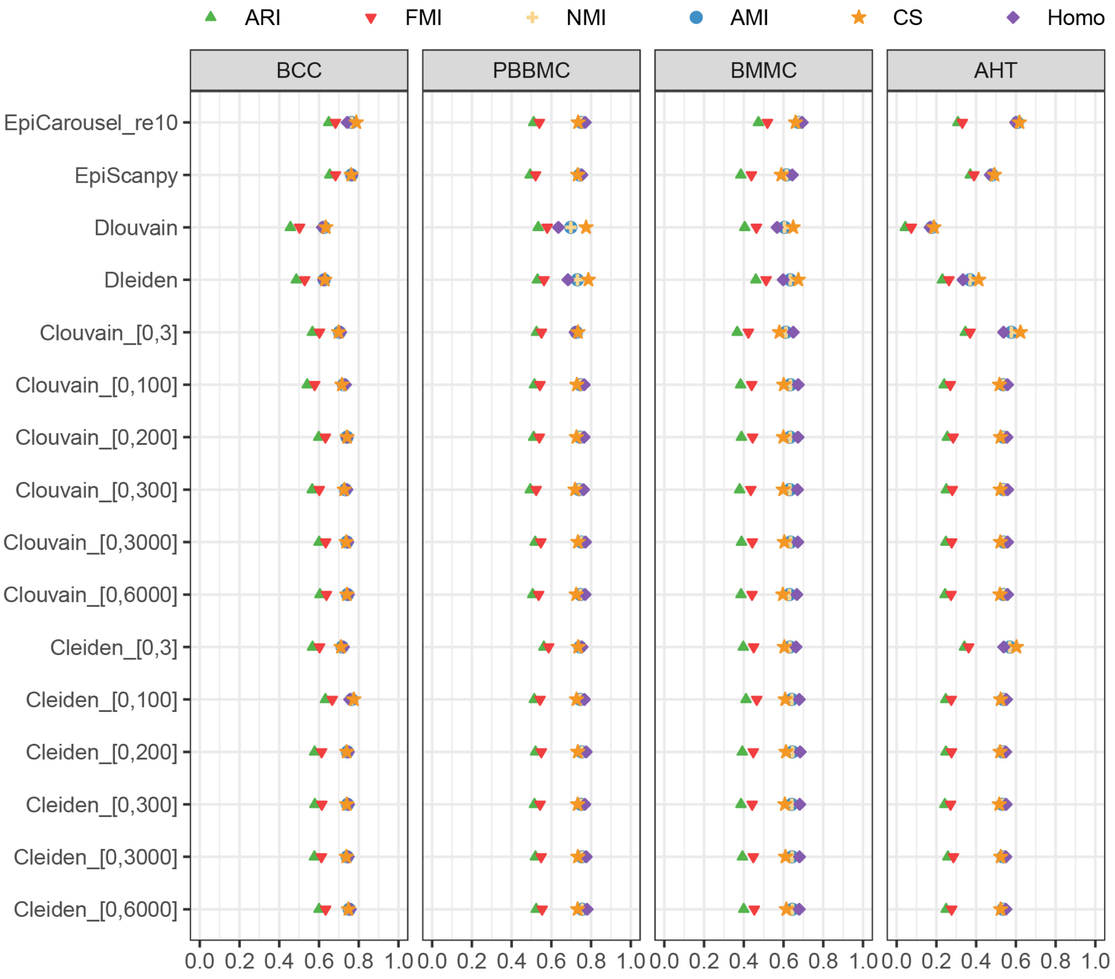

Fig. S54. Dot plots showing the clustering results of various methods using Cleiden on individual datasets. The scale of the dataset increases from left to right.

Supplementary Tables

Table S1. Summary of the 5 scCAS datasets for benchmarking

Dataset	Species	Number of cells	Number of peaks	Number of cell types	Proportion of the major type	Imbalance degree	Sparsity	Protocol	Reference
BCC	*Homo sapiens*	37,818	580,789	20	13.4%	74.7%	98.5%	scATAC-seq	(Satpathy, et al., 2019)
PBBMC	*Homo sapiens*	63,882	571,400	31	11.7%	71.5%	98.8%	scATAC-seq	(Satpathy, et al., 2019)
BMMC	*Homo sapiens*	69,249	116,490	22	16.7%	76.0%	97.0%	10X-Multiome	(Luecken, et al., 2021)
AHT	*Homo sapiens*	615,998	1,154,611	111	8.6%	70.4%	99.8%	sci-ATAC-seq	(Zhang, et al., 2021)
HFO	*Homo sapiens*	707,043	1,154,611	111	8.6%	73.7%	99.6%	sci-ATAC-seq3	(Domcke, et al., 2020)

Table S2. The number of metacells identified by each method in the comparison on five datasets

For EpiCarousel_re10 and EpiCarousel_re75, ‘No. of cells’ indicates the number of single cells contained in the input h5ad file used. For SEACells_D_re10, SEACells_A_re10, SEACells_D_re75, and SEACells_A_re75, ‘No. of cells’ represents the number of cells obtained after preprocessing the fragments file using ArchR, following the tutorials provided by SEACells (https://github.com/dpeerlab/SEACells/blob/main/notebooks/ArchR/ArchR-preprocessing-nfr-peaks.R).

Dataset	Methods	No. of cells	No. of metacells
BCC	SEACells_D_re10	36,320	3,612
BCC	SEACells_A_re10	36,375	3,638
BCC	EpiCarousel_re10	37,818	3,781
BCC	SEACells_D_re75	36,320	485
BCC	SEACells_A_re75	36,375	485
BCC	EpiCarousel_re75	37,818	503
PBBMC	SEACells_D_re75	58,771	784
PBBMC	SEACells_A_re75	58,947	786
PBBMC	EpiCarousel_re75	63,882	849
PBBMC	EpiCarousel_re10	63,882	6,388
BMMC	EpiCarousel_re10	69,249	6,924
BMMC	EpiCarousel_re75	69,249	921
AHT	EpiCarousel_re10	615,998	61,599
AHT	EpiCarousel_re75	615,998	8,192
HFO	EpiCarousel_re10	707,043	70,704
HFO	EpiCarousel_re75	707,043	9,403

Table S3. The number and proportion of cells of each cell type in the BCC dataset

Cell type	No. of cells	Proportion
Plasma B	336	0.89%
Th1	341	0.90%
B	411	1.09%
NK1	444	1.17%
Endothelial	562	1.49%
Myeloid	732	1.94%
Tumor 1	760	2.01%
Tumor 2	880	2.33%
Effector CD8 T	1,178	3.11%
Tumor 4	1,191	3.15%
NK2	1,207	3.19%
Fibroblasts	1,333	3.52%
Tumor 3	1,688	4.46%
Th17	1,892	5.00%
Naive CD8 T	2,902	7.67%
Treg	4,115	10.88%
Naive CD4 T	4,154	10.98%
Tfh	4,292	11.35%
CD8 TEx	4,333	11.46%
Memory CD8 T	5,067	13.40%

Table S4. Identified significant pathways from classical dendritic metacells via the GREAT analysis

Identified significant pathways in the GREAT analysis by the type-specific peaks obtained from classical dendritic metacells in the Immune dataset from full PBBMC dataset with EpiCarousel.

**Term Name**	**Binom raw *P*-Val**	**Binom FDR *Q*-Val**
regulation of immune system process	2.2725e-32	1.4936e-28
positive regulation of immune system process	7.4251e-32	3.2534e-28
regulation of immune response	6.3206e-30	2.0771e-26
immune response	6.7365e-30	1.7710e-26
defense response	4.6812e-23	8.7906e-20
regulation of cytokine production	9.3021e-22	1.1116e-18
leukocyte activation	1.2234e-21	1.3401e-18
positive regulation of immune response	2.9997e-21	2.8165e-18
immune response-activating signal transduction	8.4346e-21	7.3915e-18
cell activation	1.5923e-20	1.3081e-17
immune effector process	2.0221e-20	1.5636e-17
myeloid leukocyte activation	8.8721e-20	6.4791e-17
immune response-regulating signaling pathway	9.9552e-20	6.8875e-17
activation of immune response	3.1302e-19	1.9593e-16
leukocyte activation involved in immune response	1.2340e-17	6.2391e-15
regulation of leukocyte activation	1.6092e-17	7.8344e-15
cell activation involved in immune response	3.7487e-17	1.7599e-14
regulation of lymphocyte activation	4.3086e-17	1.9530e-14
myeloid cell activation involved in immune response	1.5079e-16	6.3938e-14
regulation of cell activation	7.8404e-16	2.7855e-13

Table S5. Identified significant pathways from megakaryocyte-erythroid progenitor (MEP) metacells via the GREAT analysis

Identified significant pathways in the GREAT analysis by the type-specific peaks obtained from megakaryocyte-erythroid progenitor (MEP) metacells in the Immune dataset from full PBBMC dataset with EpiCarousel.

Term Name	Binom raw *P*-Val	Binom FDR *Q*-Val
hemostasis	2.0629e-10	5.4235e-7
blood coagulation	3.8154e-10	6.2692e-7
coagulation	3.9706e-10	5.7993e-7
regulation of erythrocyte differentiation	1.1696e-7	6.1497e-5
heme biosynthetic process	1.4826e-6	4.6403e-4
regulation of myeloid cell differentiation	2.1281e-6	6.2164e-4
platelet activation	5.2169e-6	1.0550e-3
tetrapyrrole biosynthetic process	1.8555e-5	2.5948e-3
regulation of mRNA catabolic process	5.4480e-5	5.7291e-3
acyl-CoA biosynthetic process	7.1193e-5	7.2545e-3
positive regulation of nuclear-transcribed mRNA catabolic process, deadenylation-dependent decay	2.0819e-4	1.5728e-2
positive regulation of erythrocyte differentiation	2.8682e-4	2.0270e-2
definitive hemopoiesis	3.5724e-4	2.3363e-2
positive regulation of myeloid cell differentiation	4.9575e-4	3.0170e-2

Table S6. The number of metacells identified by various clustering methods on four datasets, respectively

Data	No. of cells	Method	Range of clustering resolution	No. of metacells
BCC	37,818	Walktrap	-	3,781
BCC	37,818	Dlouvain	-	81
BCC	37,818	Dleiden	-	97
BCC	37,818	Clouvain	[0,3]	158
BCC	37,818	Clouvain	[0,100]	3,126
BCC	37,818	Clouvain	[0,200]	3,775
BCC	37,818	Clouvain	[0,300]	3,768
BCC	37,818	Clouvain	[0,3000]	3,773
BCC	37,818	Clouvain	[0,6000]	3,766
BCC	37,818	Cleiden	[0,3]	182
BCC	37,818	Cleiden	[0,100]	3,272
BCC	37,818	Cleiden	[0,200]	3,777
BCC	37,818	Cleiden	[0,300]	3,787
BCC	37,818	Cleiden	[0,3000]	3,777
BCC	37,818	Cleiden	[0,6000]	3,780
PBBMC	63,882	Walktrap	-	6,388
PBBMC	63,882	Dlouvain	-	163
PBBMC	63,882	Dleiden	-	205
PBBMC	63,882	Clouvain	[0,3]	323
PBBMC	63,882	Clouvain	[0,100]	4,033
PBBMC	63,882	Clouvain	[0,200]	6,380
PBBMC	63,882	Clouvain	[0,300]	6,381
PBBMC	63,882	Clouvain	[0,3000]	6,389
PBBMC	63,882	Clouvain	[0,6000]	6,381
PBBMC	63,882	Cleiden	[0,3]	400
PBBMC	63,882	Cleiden	[0,100]	4,605
PBBMC	63,882	Cleiden	[0,200]	6,388
PBBMC	63,882	Cleiden	[0,300]	6,380
PBBMC	63,882	Cleiden	[0,3000]	6,399
PBBMC	63,882	Cleiden	[0,6000]	6,389
BMMC	69,249	Walktrap	-	6,924
BMMC	69,249	Dlouvain	-	126

Table S6 (continue). The number of metacells identified by various clustering methods on four datasets, respectively

Data	No. of cells	Method	Range of clustering resolution	No. of metacells
BMMC	69,249	Dleiden	-	148
BMMC	69,249	Clouvain	[0,3]	234
BMMC	69,249	Clouvain	[0,100]	6,768
BMMC	69,249	Clouvain	[0,200]	6,929
BMMC	69,249	Clouvain	[0,300]	6,927
BMMC	69,249	Clouvain	[0,3000]	6,925
BMMC	69,249	Clouvain	[0,6000]	6,923
BMMC	69,249	Cleiden	[0,3]	282
BMMC	69,249	Cleiden	[0,100]	6,645
BMMC	69,249	Cleiden	[0,200]	6,926
BMMC	69,249	Cleiden	[0,300]	6,912
BMMC	69,249	Cleiden	[0,3000]	6,928
BMMC	69,249	Cleiden	[0,6000]	6,906
AHT	615,998	Walktrap	-	61,599
AHT	615,998	Dlouvain	-	1,100
AHT	615,998	Dleiden	-	1,182
AHT	615,998	Clouvain	[0,3]	1,834
AHT	615,998	Clouvain	[0,100]	61,621
AHT	615,998	Clouvain	[0,200]	61,595
AHT	615,998	Clouvain	[0,300]	61,647
AHT	615,998	Clouvain	[0,3000]	61,623
AHT	615,998	Clouvain	[0,6000]	61,610
AHT	615,998	Cleiden	[0,3]	2,097
AHT	615,998	Cleiden	[0,100]	61,647
AHT	615,998	Cleiden	[0,200]	61,606
AHT	615,998	Cleiden	[0,300]	61,607
AHT	615,998	Cleiden	[0,3000]	61,639
AHT	615,998	Cleiden	[0,6000]	61,573

References

Blondel, V.D., *et al.* Fast unfolding of communities in large networks. *Journal of Statistical Mechanics: Theory and Experiment* 2008;2008(10):P10008.

Chen, S., *et al.* RA3 is a reference-guided approach for epigenetic characterization of single cells. *Nat. Commun.* 2021;12(1):2177.

Domcke, S., *et al.* A human cell atlas of fetal chromatin accessibility. *Science* 2020;370(6518).

Dominguez-Sola, D., *et al.* The FOXO1 Transcription Factor Instructs the Germinal Center Dark Zone Program. *Immunity* 2015;43(6):1064-1074.

Ferreira, M.A.R., *et al.* Genetic Architectures of Childhood- and Adult-Onset Asthma Are Partly Distinct. *Am. J. Hum. Genet.* 2019;104(4):665-684.

Finucane, H.K., *et al.* Partitioning heritability by functional annotation using genome-wide association summary statistics. *Nat. Genet.* 2015;47(11):1228-1235.

Gates, K.M., *et al.* A Monte Carlo Evaluation of Weighted Community Detection Algorithms. *Front Neuroinform* 2016;10:45.

Hao, Y., *et al.* Dictionary learning for integrative, multimodal and scalable single-cell analysis. *Nat. Biotechnol.* 2024;42(2):293-304.

Hess Michelini, R., *et al.* Differentiation of CD8 memory T cells depends on Foxo1. *J. Exp. Med.* 2013;210(6):1189-1200.

Ichii, H., *et al.* Bcl6 acts as an amplifier for the generation and proliferative capacity of central memory CD8+ T cells. *J. Immunol.* 2004;173(2):883-891.

Jeannet, G., *et al.* Essential role of the Wnt pathway effector Tcf-1 for the establishment of functional CD8 T cell memory. *Proc. Natl. Acad. Sci. U. S. A.* 2010;107(21):9777-9782.

Kim, M.V., *et al.* The transcription factor Foxo1 controls central-memory CD8+ T cell responses to infection. *Immunity* 2013;39(2):286-297.

Levine, J.H., *et al.* Data-Driven Phenotypic Dissection of AML Reveals Progenitor-like Cells that Correlate with Prognosis. *Cell* 2015;162(1):184-197.

Li, H., *et al.* Inferring transcription factor regulatory networks from single-cell ATAC-seq data based on graph neural networks. *Nat. Mach. Intell.* 2022;4(4):389-400.

Li, Z., *et al.* Chromatin-accessibility estimation from single-cell ATAC-seq data with scOpen. *Nat. Commun.* 2021;12(1):6386.

Lu, Y.C., *et al.* The Molecular Signature of Megakaryocyte-Erythroid Progenitors Reveals a Role for the Cell Cycle in Fate Specification. *Cell Rep* 2018;25(8):2083-2093 e2084.

Luckheeram, R.V., *et al.* CD4(+)T cells: differentiation and functions. *Clin. Dev. Immunol.* 2012;2012:925135.

Luecken, M., *et al.* A sandbox for prediction and integration of DNA, RNA, and proteins in single cells. In: Vanschoren, J. and Yeung, S., editors, *Proceedings of the Neural Information Processing Systems Track on Datasets and Benchmarks*. Curran; 2021.

McLean, C.Y., *et al.* GREAT improves functional interpretation of cis-regulatory regions. *Nat. Biotechnol.* 2010;28(5):495-501.

Mussbacher, M., *et al.* Mechanisms of hemostasis: Contributions of platelets, coagulation factors, and the vessel wall. *Fundamentals of Vascular Biology* 2019:145-169.

Revilla, I.D.R., *et al.* The B-cell identity factor Pax5 regulates distinct transcriptional programmes in early and late B lymphopoiesis. *EMBO J.* 2012;31(14):3130-3146.

Romano, S., *et al.* Adjusting for chance clustering comparison measures. *The Journal of Machine Learning Research* 2016;17(1):4635-4666.

Rosvall, M. and Bergstrom, C.T. Maps of random walks on complex networks reveal community structure. *Proceedings of the national academy of sciences* 2008;105(4):1118-1123.

Rubinov, M. and Sporns, O. Weight-conserving characterization of complex functional brain networks. *Neuroimage* 2011;56(4):2068-2079.

Rutishauser, R.L., *et al.* Transcriptional repressor Blimp-1 promotes CD8(+) T cell terminal differentiation and represses the acquisition of central memory T cell properties. *Immunity* 2009;31(2):296-308.

Satpathy, A.T., *et al.* Massively parallel single-cell chromatin landscapes of human immune cell development and intratumoral T cell exhaustion. *Nat. Biotechnol.* 2019;37(8):925-936.

Slowikowski, K., Hu, X. and Raychaudhuri, S. SNPsea: an algorithm to identify cell types, tissues and pathways affected by risk loci. *Bioinformatics* 2014;30(17):2496-2497.

Street, K., *et al.* Slingshot: cell lineage and pseudotime inference for single-cell transcriptomics. *BMC Genomics* 2018;19(1):477.

Stuart, T., *et al.* Single-cell chromatin state analysis with Signac. *Nat. Methods* 2021;18(11):1333-1341.

Su, A.I., *et al.* A gene atlas of the mouse and human protein-encoding transcriptomes. *Proceedings of the National Academy of Sciences* 2004;101(16):6062-6067.

Tang, Y., *et al.* A preliminary study of KAT2A on cGAS-related immunity in inflammation amplification of systemic lupus erythematosus. *Cell Death Dis.*

2021;12(11):1036.

Traag, V.A., Waltman, L. and van Eck, N.J. From Louvain to Leiden: guaranteeing well-connected communities. *Scientific Reports* 2019;9(1).

Ubieta, K.*, et al.* Fra-2 regulates B cell development by enhancing IRF4 and Foxo1 transcription. *J. Exp. Med.* 2017;214(7):2059-2071.

Zhang, K.*, et al.* A single-cell atlas of chromatin accessibility in the human genome. *Cell* 2021;184(24):5985-6001 e5919.

Zhao, D.M.*, et al.* Constitutive activation of Wnt signaling favors generation of memory CD8 T cells. *J. Immunol.* 2010;184(3):1191-1199.
